# Association between maternal iron deficiency and delayed neonatal auditory maturation and altered cochlear synaptic energy metabolism: analysis from a mother–infant observational study, mouse models, and cochlear explants

**DOI:** 10.3389/fnut.2026.1842147

**Published:** 2026-06-19

**Authors:** Yi Zhao, Rui Hou, Wei Song, Wei Shao, Lifeng Zhang, Fei Yu, Shuai Hao

**Affiliations:** 1 Department of Gynaecology and Obstetrics, First Affiliated Hospital of China Medical University, Shenyang, China; 2 Department of Gynaecology and Obstetrics, Shengjing Hospital of China Medical University, Shenyang, China; 3 Liaoning Institute of Basic Medical Sciences, Shenyang, China; 4 Department of Maternal, Child and Adolescent Health, School of Public Health, Shenyang Medical College, Shenyang, China; 5 Department of Otolaryngology, First Affiliated Hospital of China Medical University, Shenyang, China

**Keywords:** cochlea, energy metabolism, hearing loss, iron deficiency, ribbon synapses

## Abstract

**Background:**

Iron is a key nutrient for the development of the fetal auditory system. However, the potential impact of non-anemic prenatal iron deficiency (ID) on neonatal auditory function remains unclear. This study aimed to systematically explore the potential mechanisms by which maternal ID may affect auditory maturation of offspring.

**Methods:**

We analyzed population data from 696 mother–infant pairs, established ID mouse models (C57BL/6 J) during pregnancy, and conducted cellular experiments.

**Results:**

In the human cohort, maternal serum ferritin (SF) and hemoglobin (Hb) were significantly negatively associated with the latency (ms) of auditory brainstem response (ABR) waves I, III, and V, as well as intervals (ms) of waves I–III, III–V, and I–V and summating potential/action potential ratios (%). Neonatal SF partially mediated the association between maternal iron status and auditory function, with mediation effects ranging from 28.57 to 76.32%. In mouse models, prenatal ID was associated with decreased wave I amplitude and extended latency in offspring, along with reduced ribbon synapses in inner hair cells, mitochondrial damage, and decreased enzyme activity in supporting cells. A metabolomics analysis revealed significant downregulation of pyruvate levels in the ID group, and exogenous supplementation with sodium pyruvate partially restored ribbon synaptic function. Collectively, prenatal ID may reduce fetal iron reserves, impair energy metabolism of cochlear supporting cells, inhibit ribbon synaptic maturation, and potentially contribute to auditory dysfunction.

**Conclusion:**

Our findings suggest that non-anemic maternal ID may be associated with delayed neonatal auditory maturation, highlighting the potential importance of iron intervention during pregnancy for improving neonatal auditory outcomes; however, causal relationships cannot be established from the observational human data, and the animal/cellular findings should be interpreted as supportive evidence requiring further validation.

## 1 Introduction

Iron is an essential micronutrient critical for early life cognitive, motor, immune function, and auditory system development (1). The fetus obtains iron primarily from the mother through the placenta, with approximately 80% of placental iron transport occurring during late pregnancy, which is a critical period for fetal iron reserve accumulation (2). Iron deficiency (ID) affects 30–60% of pregnant women globally (3, 4) and is a particular concern in resource-limited settings (5). While iron deficiency anemia (IDA) has been associated with an increased risk of hearing loss across the lifespan (6–8), whether non-anemic maternal ID affects neonatal auditory development remains poorly understood.

Iron plays key roles in neuronal differentiation, axonal growth, and neurotransmitter synthesis (9–11). In the cochlea, iron is essential for mitochondrial function and energy metabolism in supporting cells (8). The large epithelial ridge, a transient structure in early cochlear development, provides metabolic and mechanical support for ribbon synapse formation in inner hair cells (IHCs) (12). This synaptic refinement process depends heavily on adenosine triphosphate (ATP) supply (13); however, the relationship between iron metabolism and cochlear energy homeostasis in developing auditory synapses remains unclear.

Although prior studies have established associations between IDA and auditory impairment (14–17), evidence on non-anemic ID and neonatal hearing outcomes is limited.

In particular, the mechanistic pathway linking maternal iron status to offspring auditory function, including the potential mediating role of neonatal iron stores and the contribution of cochlear energy metabolism, has not been systematically investigated.

This study aimed to examine the association between non-anemic maternal ID and neonatal auditory function in a human cohort and to explore the underlying mechanisms using gestational ID mouse models and *in vitro* experiments. We hypothesized that maternal ID may affect neonatal auditory maturation through pathways involving fetal iron reserves, cochlear supporting cell energy metabolism, and ribbon synaptic development.

## 2 Methods

### 2.1 Human mother–infant observational study

#### 2.1.1 Study design, setting, and participants

This survey adopted a cluster sampling method to continuously include 696 healthy pregnant women (18–40 years old) and their newborns who underwent pregnancy examinations at the Obstetrics Clinic of the First Affiliated Hospital of China Medical University from November 2013 to May 2017. The research protocol was reviewed by the Ethics Committee of China Medical University [Approval No. CMU20142927], and all participants provided informed consent. The enrolled pregnant women signed an informed consent form.

#### 2.1.2 Inclusion and exclusion criteria

The inclusion criteria were as follows: (1) pregnant women who were permanent residents of Liaoning Province and Chinese residents who had resided in the surveyed area for more than 6 months within the 12 months prior to the survey and (2) women with a singleton pregnancy between 32 and 36 weeks of gestation. The exclusion criteria were as follows: (1) gestational disorders, including anemic pregnant women; premature or expired birth, low birth weight or macrosomia, perinatal birth injury, and difficult delivery; history of suffocation, hypoxia, epilepsy, febrile seizures, and other central nervous system diseases; organic diseases such as congenital heart disease, repeated chronic or acute diseases such as diarrhea and infection during the examination period, or acute or chronic blood loss, and (2) infant diseases, nutritional diseases such as abnormally high-sensitivity C-reactive protein, hypothyroidism, high lead or zinc deficiency, and active rickets; genetic metabolic diseases; children with birth defects, and a lack of physical examination information for newborns.

#### 2.1.3 Maternal and neonatal clinical data collection

The trained team conducted a questionnaire survey at enrollment and subsequently supplemented basic information of the offspring after delivery. Through face-to-face survey of the mother, her demographic data and lifestyle were collected, which were as follows: (1) sociodemographic data (age, occupation, annual family income, educational level, etc.); (2) birth history and basic information (gestational age, pre-pregnancy weight, pre-pregnancy waist circumference, height, live birth history, history of abnormal pregnancy and childbirth, pregnancy-related vomiting, and use of assisted reproductive technology has been received, etc.); (3) disease and surgical history (history of diabetes, hypertension, cardiovascular disease, pregnancy diabetes, etc.) and family history (family history of diabetes during pregnancy, family history of hypertension during pregnancy, family history of tumor or cancer, etc.); and (4) personal lifestyle habits (extra exercise during pregnancy, smoking before pregnancy, drinking before pregnancy, passive smoking during pregnancy, etc.). The physical examination of newborns included Apgar scores and body weight, which were obtained from clinical data obtained from the Department of Obstetrics and Gynecology of First Affiliated Hospital of China Medical University. The clinical data of pregnant women included Hb, blood pressure, fasting blood glucose (FBG), and delivery mode.

#### 2.1.4 Assessment of maternal and neonatal iron status

Both maternal and umbilical cord samples were sent to the institutional laboratory of the First Affiliated Hospital of China Medical University for serum ferritin (SF) measurement using a chemiluminescent immunoassay (10). Umbilical cord blood was collected and stored at 4 °C. Because severe hemolysis of samples erroneously elevated SF levels, cord SF samples with severe hemolysis were not considered for measurement.

Based on the 2014 Diagnosis and Treatment Guidelines for ID and IDA in Pregnancy in China, the study subjects with SF < 20 μg/L were diagnosed as ID and those with Hb < 110 g/L were diagnosed as IDA (5). Neonatal SF was measured using umbilical cord blood collected immediately after delivery. The medial and lateral aspects of the newborn’s heel were disinfected with 75% alcohol over an area greater than 3 cm in diameter, and the procedure was initiated after allowing the area to dry. The phlebotomist stabilized the newborn’s heel with the left hand while holding the lancet in the right hand to puncture the medial or lateral aspect of the heel at a depth of approximately 2–3 mm. After the puncture, the area surrounding the puncture site was gently squeezed, and the first drop of freely flowing blood was wiped away with a sterile cotton swab. When a sufficient blood drop had formed, blood was collected to fill three consecutive blood spots, and the collected samples were then sent to the laboratory for analysis.”

#### 2.1.5 Neonatal auditory assessment: auditory brainstem response (ABR) and electrocochleography (ECochG)

Newborn hearing assessments included the auditory brainstem response (ABR) test and electrocochleography (ECochG) within 48–72 h of birth, which were based on a previous study (12). ABR testing was performed on participants using a great nordic (GN) ear force tester under natural sleep conditions in a sound-insulated and -shielded room with the intelligent hearing systems (IHS) Smart Ethanol Protocol (EP) auditory evoked potential instrument. The stimulation signal was a short sound with alternating polarity, with a pulse width of 0.1 ms and a repetition rate of 19.3 times/s. The recording electrode was placed on the forehead hairline, the reference electrode was placed on the ipsilateral mastoid, and the contralateral mastoid was grounded. Before placing the electrode, the local skin was degreased with an alcohol cotton swab. The test was primarily used to assess peripheral auditory sensitivity and nerve conduction function in the brainstem auditory pathway. The inter-electrode resistance is less than 10 KΩ. The filter band-pass range was set at 100–1500 Hz. The ABR results from the ear with the shorter V-wave latency were selected to measure the absolute latencies of waves I, III, and V and the wave intervals (I–III, III–V, and I–V waves).

ECochG was performed to record three distinct potentials, including action potential (AP) and summating potential (SP). Measurements were acquired using alternating-polarity short-tone bursts with a tympanic membrane electrode. Following cleaning of the external auditory canal and tympanic membrane with acetone solution, electrodes were positioned on the posterior-inferior surface of the tympanic membrane for signal recording. Click stimuli (0.1 ms, 70 dB nHL) were delivered to the test ear via an insert earphone (ER-3A) at a rate of 7.1/s. Waveforms were collected and filtered through a bandpass filter (5–2000 Hz) before averaging. The SP/AP ratio was calculated for each recording. These parameters served as indicators of auditory nerve maturation across multiple levels of the auditory pathway. Impaired auditory neural maturation was defined as values exceeding the 50th percentile for absolute wave V latency, IPL III–V, and the SP/AP ratio.

### 2.2 Mouse model and *in vivo* assessments

#### 2.2.1 Animals, dietary intervention, and mating protocol

C57BL/6 J mice (female, *n* = 12; male, *n* = 6) aged 10 weeks were selected with sensitive bilateral ear reflexes. All protocols have been approved by the Animal Ethics Committee of China Medical University [Approval No. CMU20241929]. Female mice were randomly assigned to one of two dietary groups as follows. The control group was fed a normal diet containing iron content of > 150 mg/kg diet, as measured by atomic absorption spectrometry. The iron content was prepared in the laboratory by adding ferrous sulfate heptahydrate to the AIN-93G purified rodent formulation (18). The ID group was fed an iron-deficient diet containing 5 mg iron/kg diet. The composition of iron-deficient diet was based on previous studies (Supplementary Table S1) (19, 20). After acclimation for 2 weeks, two female mice were placed with one proven male for mating. The day on which a vaginal plug was found was considered gestational day (GD) 0. Each litter was culled to 8 pups (4 male and 4 female) on postnatal day (PND) 4.

#### 2.2.2 Assessment of maternal and offspring iron status

The whole blood of one mouse from each litter was collected via the enucleation method, and hemoglobin (Hb), hematocrit (HCT), and other indicators were measured using a fully automated blood cell analyzer. Serum ferritin (SF) levels were determined using ELISA, and serum iron (SI) concentrations were measured using the ferrous cyanide colorimetric method. At each time point of monitoring the mother and offspring mice, animals were anesthesized under general anesthesia before blood collection. Blood was collected vertically from the orbit into the centrifugal tubes containing anticoagulants for whole blood analysis and into centrifugal tubes without anticoagulant for serum detection. Blood from the orbit flowed vertically into the centrifugal tube, which was coagulated at room temperature for 1–2 h. After collection of blood samples, serum was separated (3,000 rpm, 5 min) and stored at −70 °C. Serum samples were analyzed for SI concentrations by atomic absorption spectrophotometry. Hb was measured using the colorimetric cyanmethemoglobin method. Hct was measured by blood centrifugation in micro-capillary tubes and read using a microhematocrit reader. SF was measured using the Ferritin ELISA kit and read with a microtiter plate reader.

#### 2.2.3 Selection of developmental time points

Based on the postnatal developmental trajectory of the murine cochlea, PND2, PND7, and PND14 were selected to represent early, intermediate, and relatively mature stages of cochlear development, respectively (21). PND2 was used for analyses of GER-associated supporting cells; PND7 for the assessment of energy-metabolism–related parameters; and PND14 for the evaluation of synaptic and morphological maturation. PND 7, as an important time point in the moderate maturation stage of the auditory system, not only detects the dynamic changes of its energy metabolism but also evaluates the potential impact of maternal ID on iron reserves of offspring through SF detection. Due to the feasibility of cochlear perfusion technology in this period, the tetranitroblue tetrazolium chloride (NBT) staining method was used to analyze the succinate dehydrogenase (SDH) activity characteristics simultaneously. Considering that the auditory function of PND 14 mice approaches maturity (similar to the hearing level of human newborns), we further systematically analyzed the effects of gestational ID on auditory nerve development, myelination, and auditory physiological function using immunofluorescence and transmission electron microscopy. We also comprehensively evaluated its effects on the SF level, cochlear iron homeostasis, and auditory functional maturity of the offspring and analyzed the morphological and ultrastructural changes of cochlea. On PND 2, the cochlear basement membranes of three from each litter were observed for the morphology, including HE staining, immunofluorescence (Myosin VIIa, CtBP2, and GluR2 labeling), and scanning electron microscopy (SEM). These methods were performed as previously described (22). On PND 7, the fresh cochlear tissue of two from each litter was observed for cochlear energy metabolism through NB staining, NAD^+^ level measurement, and NAD^+^/NADH ratio analysis, which were based on a previously described study (23). The remaining offspring were weaned on PND 21 and tested for ABR based on a previous study (22).

#### 2.2.4 ABR recording in offspring

ABRs were recorded using subcutaneous silver wire electrodes being inserted ventrolateral to the right ear (active) and at the vertex (negative), with a ground electrode placed at the lower back. The sound delivery tube of an insert earphone was tightly fitted into the external auditory canal. A subcutaneous needle electrode active lead was positioned at the vertex and referenced to a second electrode at the tip of the nose. The ground electrodes were placed over the neck muscles. Tone burst stimuli, with a 0.2-ms rise/fall time (cosine gate) and 1-ms flat segment at frequencies of 4, 8, 16, and 32 kHz, were generated, and the amplitude was specified by a sound generator, an attenuation real-time processor, and a programmable attenuator (Tucker-Davis Technology, Alachua, FL, USA). Sound-level calibrations were performed using a Sound Level Meter (Rion, Tokyo, Japan). ABR waveforms were recorded for 12.8 ms at a sampling rate of 40,000 Hz using 50- to 5,000-Hz band-pass filter settings; waveforms from 256 stimuli at a frequency of 9 Hz were averaged. ABR waveforms were recorded in 10-dB sound pressure level (SPL) intervals down from a maximum amplitude until no waveform could be visualized. The ABR threshold was defined as the lowest intensity capable of eliciting a replicable and visually detectable response with at least two peaks and a minimum amplitude of 0.5 μV. Wave amplitude was analyzed. Each wave at high levels (70–90 dB SPL) consisted of one negative (n) deflection and one positive (p) deflection. This positive peak is followed by a negative deflection. ABR wave I amplitude analysis consists of three parts: time of peak (from beginning to Ip), SPL of click, and amplitude of wave I (Ip-In) (latency, 1.2–1.9 ms). An algorithm for an automated determination of ABR amplitudes was programmed in MATLAB (MathWorks).

#### 2.2.5 Cochlear histology and ultrastructural observation

The temporal bone was removed, and the cochlea was quickly separated. The round and oval windows were opened, and they were perfused with 2.5% glutaraldehyde for SEM or 4% paraformaldehyde for immunostaining at 4 °C overnight. The cochlear shell was separated from the basal turn under a dissecting microscope in 0.1 M phosphate-buffered saline (PBS, pH 7.2). The parietal gyrus of the basilar membrane was also separated. In addition, the vestibular membrane and cover membrane were removed. The auditory nerves were processed for light microscopy using the standard method consisting of fixation in formalin, decalcification using EDTA, embedding in celloidin, serial sectioning along the cochlear modiolus direction at a section thickness of 20 μm, and H&E staining of every sixth section. Spiral ganglion cell (SGC) counts were performed for the basal, middle, and apical turns of the cochleae on the HE-stained sections. The areas of the Rosenthal canal and the cochlear turn were quantified by measuring their cut surfaces using ImageJ software. All neurons meeting the size and shape criteria to be considered type 1 SGCs within each profile of Rosenthal canal were counted for the basal, middle, and apical turns of the cochleae. The SGC density was determined as the number of cell nuclei per 10,000 μm^2^ Rosenthal canal.

The cochlea was perfused with 2.5% glutaraldehyde and fixed at 4 °C for 24 h. The organ of Corti was dissected using an anatomy microscope. The tissues were post-fixed with 1% osmium tetroxide in 0.1 M PBS for 2 h at room temperature and dehydrated in graded ethanol solutions from 50 to 100% (each for 30 min). Then, the specimens were dried in an HCP-2 critical point dryer and sputter-coated with platinum for 4 min in an E-102 ion sputter. The specimens were examined by a JEOL JSM-35C SEM (Hitachi 7,100, Tokyo, Japan). The images were recorded digitally and photographed. Cell density was defined as the number of counted cells from a distance along the basilar membrane divided by that distance. The average densities of IHCs and OHCs were calculated for each cochlea. The average cell counter per 100 μm in the cochleae was analyzed to obtain values of cell densities of IHCs and OHCs using a log-linear model (Poisson regression) for count data with log (Distance) as an offset and a random effect for variation between preparations.

#### 2.2.6 Immunofluorescence and ribbon synapse quantification

The separated basilar membranes were fixed in 4% paraformaldehyde and dissolved in 0.1 M PBS with 30% sucrose for 1 h at room temperature. Next, these samples were washed three times in 0.01 M PBS and preincubated for 30 min at room temperature in a blocking solution of 5% normal goat serum in 0.01 M PBS with 0.3% Triton X-100 and then incubated with anti-CtBP2, myosin VIIa, GluR2, and Sox2 at 4 °C for 24 h. Then, the incubated samples were washed out in 0.01 M PBS three times, and incubated with the secondary antibody Alexa Fluor 488 goat anti-mouse IgG, Alexa Fluor 494 goat anti-rabbit IgG at 37 °C for 2 h. After incubation, the samples were washed in PBS twice. After dropping a small amount of DAPI onto the slide, basement membranes were tiled under a dissecting microscope; the coverslip covered the slide. The samples were imaged directly with a confocal laser scanning microscope (FV1000, Olympus; emission wavelengths 488 and 494 nm) to test the specificity of the primary antibody. The number of RIBEYE/CtBP2 positive spots was counted in each IHC. To determine the average number of labeled spots in each IHC, total values of labeled spots from 7 to 10 IHCs per location (the basal, middle, or apical turn) in each cochlea (8 cochleae per group) were counted, and subsequently, the average numbers of labeled spots in each IHC were calculated.

### 2.3 Cochlear explant and supporting-cell experiments

#### 2.3.1 Cochlear explant preparation and DFO-induced iron depletion

On PND 2, mouse cochlear explants were isolated. To deplete the intracellular iron inventory and induce an iron-deficient environment, 100 𝜇mol/L, 300 𝜇mol/L, and 500 𝜇mol/L desferrioxamine (DFO) were added to the DFO group explants. After 24 h of cultivation, subsequent experiments were conducted.

RhoNox-1 probe was used to label Fe^2+^, while Fluo-4 a.m. was used to detect intracellular Ca^2+^ fluctuations, and cell viability was assessed. Intracellular and extracellular ATP levels were quantified as indicators of cellular energy status. These methods were based on previously described Ref. [24].

Targeted energy metabolomics analysis was performed based on a previous study (25). In total, 100 μmol/L DFO was added to the explants of the DFO group, and an equal amount of PBS was added to the Control group. After 24 h of drug treatment, energy metabolomics analysis was performed using liquid chromatography tandem mass spectrometry (LC–MS/MS).

#### 2.3.2 Fe^2+^ fluorescence detection

Cochlear explants were digested in d-Hanks’ solution containing 0.5 mg/mL thermolysin and 5 μg/ml DNase for 30 min. When the tissue structure becomes loose, the cell clamp was used to lift and separate the CES under the microscope; then, it was transferred to the Petri dish containing PBS, and the digestion was terminated. Under the microscope, the inner hair cells and outer hair cells were clearly visible, and there was a clear boundary between the large epithelial ridge and hair cells. The large epithelial ridge was carefully separated along the demarcation line with a cell clamp. An appropriate amount of 4% PFA solution was added dropwise and fixed for 15 min after moistening with PBS, then permeabilized and blocked with PBT-1 containing 0.3% Triton X-100 and 5% Donkey Serum for 30 min at room temperature. Then, 100 μl of 1:500 rhonox-1 labeled Fe^2+^ was added and incubated at room temperature for 30 min. Samples were gently soaked with PBS three times for 5 min. An anti-fluorescence quenching blocking agent containing DAPI was added to seal the film, and then it was observed, and photographs were taken under a fluorescence microscope. The protein fluorescence intensity was calculated.

#### 2.3.3 ATP measurement

The isolated large epithelial ridge supporting cells were seeded in 96-well culture plates for extracellular ATP and intracellular ATP detection experiments. For extracellular ATP detection, first, 100 μl fresh culture medium was changed per well, then corresponding treatment reagents were added according to different experimental groups, and finally, the cell culture supernatant was carefully collected after 24 h of continuous culture in standard culture conditions. An equal volume of ATP detection buffer was added to the collected supernatant sample, and a multifunctional microplate reader was immediately used to conduct quantitative determination of ATP level at a preset wavelength. In order to eliminate the influence of the difference in the number of cells among the groups on the experimental results, the total protein content of cells was measured synchronously, and the final measured ATP level was standardized and corrected according to the protein content.

For intracellular ATP detection, after 24-h culture under different treatment conditions, the culture medium in each well was discarded first, and the cells were gently washed three times with precooled PBS to remove the residual medium. We transferred the cell lysate to a centrifuge tube, centrifuged at 12,000 rpm for 5 min at 4 °C, and carefully aspirated the supernatant as the sample to be tested. Subsequently, ATP detection buffer was also added, and the ATP level was determined using a microplate reader. Finally, it was also standardized according to the protein content to eliminate potential systematic errors and ensure the reliability and comparability of the experimental data.

#### 2.3.4 NAD^+^/NADH assay

Two explants were pooled as one sample, with three samples included in each group. After washing with PBS, samples were added to a 200 μl NAD^+^/NADH extraction buffer for homogenization and centrifuged at 16,000 rpm for 10 min.

#### 2.3.5 Mitochondrial membrane potential assay

The fluorescent probe JC-1 was used to detect the mitochondrial membrane potential of supporting cells in the large epithelial ridge. In a 12-well plate, JC-1 (10 *μ* m) was added to each well to incubate for 30 min, and the cells were observed and photographed using a fluorescence microscope.

#### 2.3.6 Lactate and enzyme activity assays

The lactic acid content was determined using the lactic acid content detection kit. The quantitative analysis of the activities of cochlear basement membrane explants was carried out using the activity detection kits.

#### 2.3.7 Targeted energy metabolomics

In total, 100 μmol/L DFO was added to the cochlear explants of the DFO group, and the same amount of PBS was added to the control group. After 24 h of drug treatment, 500 μL supernatant was taken, and three samples in each group were analyzed in parallel, and energy metabolomics was detected. Liquid chromatography tandem mass spectrometry (LC–MS/MS) was used. Sciex QTRAP® 6,500 + tandem mass spectrometer was used for data collection. The multiple reaction monitoring (MRM) mode of triple quadrupole mass spectrometry was used for quantitative analysis, and Analyst 1.6.3 and Multiquant 3.0.3 software were used for data analysis.

#### 2.3.8 Intracellular Ca^2+^ imaging and rescue experiments

The cochlear explants were removed from the incubator, and the original culture medium was discarded. After washing three times with PBS at room temperature, they were immersed in a 5 μm fluo-4 a.m. fluorescent dye (avoid light throughout). Following immersion, the explants were returned to the incubator for incubation for 15 min and washed three times with PBS. Subsequently, 2 mL PBS was added to immerse the basement membrane and returned back to the incubator for 15 min. The stock solution was discarded, and samples were fixed with 4% PFA, fixed for 30 min at room temperature, and finally washed three times with PBS. After the anti-fluorescence quenching sealing agent containing DAPI was added dropwise, the film was sealed, and the results were observed by a laser confocal microscope. The optical density of positive products in each group was determined by ImageJ image analysis software, and the value represents the concentration of Ca^2+^.

### 2.4 Statistical analysis

Descriptive statistics were used to present the demographic characteristics of pregnant women, basic information of newborns, and distribution characteristics of auditory physiological indicators. Continuous variables were expressed as mean ± standard deviation (SD), while categorical variables were expressed as frequency and percentage (*n*, %). A univariate analysis was used by independent sample t-test or an analysis of variance (ANOVA) to compare differences between or among groups, and Pearson’s correlation analysis was used to assess the linear relationships between continuous variables. For the correlation analysis between categorical variables and other indicators, the chi-square test or Fisher’s exact test was used.

For the univariate analysis, a generalized linear model (GLM) was further used for the multivariate analysis to adjust for potential confounding variables and evaluate the independent effects of maternal and neonatal iron metabolism indicators (SF and Hb) on various auditory physiological indicators. To avoid multicollinearity, independent models containing different iron metabolism indicators were constructed separately, and standardized regression coefficients (*β*) and 95% confidence intervals (CI) were calculated. The model fitting adopts maximum likelihood estimation, with a significance level of *α* = 0.05.

The mediation effect analysis was used by Hayes PROCESS macro Model 4 and Bootstrap method (1,000 repeated samples) to test the mediating effect of neonatal SF between maternal SF/Hb and auditory indicators. The direct effect, indirect effect, and their proportion (effect size) were calculated. Animal experimental data were compared between groups using an independent sample *t*-test or the Mann–Whitney U test, and a *p-*value of < 0.05 was considered statistically significant. All analyses were conducted using SPSS 20.0 and GraphPad Prism 8.0.

## 3 Results

### 3.1 Population

#### 3.1.1 Baseline characteristics of the population

Due to the exclusion of anemia patients (Hb < 110 g/L), the average Hb of pregnant women was relatively high (126.66 ± 10.49 g/L), and the mean SF was (27.72 ± 11.94) μg/L, ranging from 6.00 to 105.76 μg/L. Among them, ID pregnant women (SF < 20 μg/L) accounted for 34.30%. The proportion of female infants was 40.4% (*n* = 281). The average SF in newborns was (92.28 ± 23.97) μg/L, ranging from 23 to 208 μg/L. There was no statistical difference in other indicators between ID and control (Table 1).

**Table 1:** Basiline characteristics of pregnant women and offspring.

Baseline characteristics	All (*n* = 696)	Control (*n* = 576)	ID (*n* = 120)	*p*-value
Age (years)				0.884
<35	592 (85.1)	493 (85.5)	99 (82.5)	
35–44	104 (14.9)	83 (14.5)	21 (17.5)	
Education				0.382
Junior school	161 (23.1)	139 (24.1)	22 (18.3)	
High school	338 (48.6)	277 (48.1)	61 (50.8)	
University and college	197 (28.3)	160 (27.8)	37 (30.8)	
Occupation				0.336
Farmers, workers, unemployed	262 (37.6)	223 (38.7)	39 (32.5)	
Businessmen or employees	267 (38.4)	220 (38.2)	47 (39.2)	
Leaders, government employees	167 (24.0)	133 (23.1)	34 (28.3)	
Household income (RMB/month)				0.536
<4,000	238 (34.2)	199 (34.5)	39 (32.5)	
4,000–10,000	294 (42.2)	246 (42.7)	48 (40.0)	
>10,000	164 (23.6)	131 (22.7)	33 (27.5)	
Hb (g/L)	126.66 ± 10.49	129.12 ± 9.81	114.87 ± 2.83	0.000
SF (μg/L)	27.72 ± 11.94	32.25 ± 12.16	16.03 ± 9.29	0.000
Pre-pregnancy BMI (kg/m^2^)				0.239
<24	540 (77.6)	442 (76.7)	98 (81.7)	
≥24	156 (22.4)	134 (23.3)	22 (18.3)	
Fasting blood glucose				0.066
<5.1 mmol/L	548 (78.7)	461 (80.0)	87 (72.5)	
≥5.1 mmol/L	148 (21.3)	115 (20.0)	33 (27.5)	
Hypertension				0.603
No	670 (96.3)	553 (96.0)	117 (97.5)	
Yes	26 (3.7)	23 (4.0)	3 (2.5)	
Extra-sport activities				0.878
No	393 (56.5)	326 (56.6)	67 (55.8)	
Yes	303 (43.5)	250 (43.4)	53 (44.2)	
Pre-pregnancy smoking		()	()	0.207
No	691 (99.3)	573 (99.5)	118 (98.3)	
Yes	5 (0.7)	3 (0.5)	2 (1.7)	
Passive smoking during pregnancy				0.203
No	541 (77.7)	453 (78.6)	88 (73.3)	
Yes	155 (22.3)	123 (21.4)	32 (26.7)	
Pre-pregnancy drinking				0.926
No	628 (90.2)	520 (90.3)	108 (90.0)	
Yes	68 (9.8)	56 (9.7)	12 (10.0)	
Dietary pattern				0.685
Vegetarian diet	153 (22.0)	130 (22.6)	23 (19.2)	
Meat diet	228 (32.8)	186 (32.3)	42 (35.0)	
Mixed diet	315 (45.3)	260 (45.1)	55 (45.8)	
Conception				0.863
Natural conception	653 (93.8)	540 (93.8)	113 (94.2)	
Assisted reproduction	43 (6.2)	36 (6.2)	7 (5.8)	
Delivery				0.356
Vaginal birth	299 (43.0)	252 (43.8)	47 (39.2)	
Cesarean	397 (57.0)	324 (56.2)	73 (60.8)	
Offspring				
Gender				0.910
Boys	415 (59.6)	344 (59.7)	71 (59.2)	
Girls	281 (40.4)	232 (40.3)	49 (40.8)	
Gestational age (week)	39.44 ± 1.95	39.44 ± 1.94	39.41 ± 2.02	0.868
Birth weight (kg)	3.50 ± 0.27	3.50 ± 0.27	3.48 ± 0.27	0.515
Neonatal SF (μg/L)	92.28 ± 23.97	94.42 ± 17.74	87.65 ± 27.27	0.001
Apgar score	9.99 ± 0.10	9.99 ± 0.11	10.00 ± 0.01	0.388
Wave I absolute latency (ms)	1.75 ± 0.32	1.74 ± 0.33	1.79 ± 0.28	0.092
Wave III absolute latency (ms)	4.20 ± 0.57	4.21 ± 0.59	4.21 ± 0.51	0.998
Wave V absolute latency (ms)	5.87 ± 0.79	5.90 ± 0.80	5.76 ± 0.70	0.056
I-III interpeak latency (ms)	2.44 ± 0.02	2.43 ± 0.41	2.47 ± 0.45	0.274
III-V interpeak latency (ms)	2.19 ± 0.46	2.20 ± 0.47	2.14 ± 0.38	0.152
I-V interpeak latency (ms)	4.62 ± 0.76	4.62 ± 0.78	4.61 ± 0.70	0.894

Data are shown as *n* (%) or mean ± SD. ID, Iron deficiency; BMI, Body mass index; Hb, Hemoglobin; SF, Serum ferritin.

#### 3.1.2 Auditory physiological indicators in newborns between sexes

To verify whether gender was a confounding factor, we described the auditory physiological characteristics of newborns between sexes (Table 2). No statistical difference in auditory physiological indicators were observed between male and female newborns (Table 2).

**Table 2:** . Auditory physiological indicators in newborns between different genders.

Characteristics	All	Boys	Girls	*p*
Wave I absolute latency (ms)	1.75 ± 0.01	1.76 ± 0.32	1.75 ± 0.31	0.370
Wave III absolute latency (ms)	4.20 ± 0.22	4.19 ± 0.59	4.22 ± 0.56	0.432
Wave V absolute latency (ms)	5.87 ± 0.30	5.85 ± 0.82	5.91 ± 0.72	0.140
I-III interpeak latency (ms)	2.44 ± 0.02	2.44 ± 0.42	2.43 ± 0.41	0.617
III-V interpeak latency (ms)	2.19 ± 0.02	2.18 ± 0.46	2.19 ± 0.45	0.983
I-V interpeak latency (ms)	4.62 ± 0.03	4.62 ± 0.76	4.62 ± 0.76	0.929
SP/AP (%)	26.59 ± 3.84	26.47 ± 3.82	26.78 ± 3.87	0.858

Data are shown as mean ± SD. AP, Action potential; SP, Summating potential.

#### 3.1.3 Univariate analysis of maternal factors on auditory physiological indicators

A significant correlation was observed between pre-pregnancy alcohol consumption and multiple interpeak latencies in newborns. Compared with newborns of pregnant women without a history of alcohol consumption before pregnancy, newborns of those with a history of alcohol consumption before pregnancy exhibited prolonged auditory wave intervals: wave I–III interpeak latency (2.42 ± 0.40 ms vs. 2.64 ± 0.48 ms, *p* < 0.001), wave III–V interpeak latency (2.18 ± 0.45 ms vs. 2.31 ± 0.50 ms, *p* < 0.05), and wave I–V interpeak latency (4.59 ± 0.74 ms vs. 4.93 ± 0.88 ms, *p* < 0.01) (Table 3).

**Table 3:** Univariate analysis of maternal factors on neonatal auditory physiological indicators.

Characteristics	Absolute latency (ms) (*x̄* ± *s*, *p*)			Interpeak latency (ms) (*x̄* ± *s*, *p*)			SP/AP(%, *p*)
	Wave I	Wave III	Wave V	I-III	III-V	I-V	
Age (years)							
<35	1.74 ± 0.32, 0.553	4.20 ± 0.58, 0.300	5.86 ± 0.80, 0.347	2.43 ± 0.41, 0.167	2.19 ± 0.46, 0.717	4.62 ± 0.77, 0.575	26.54 ± 3.90, 0.444
35–44	1.77 ± 0.31	4.26 ± 0.54	5.94 ± 0.72	2.49 ± 0.42	2.17 ± 0.44	4.66 ± 0.72	26.86 ± 3.49
Education							
Junior school	1.75 ± 0.34, 0.988	4.18 ± 0.62, 0.797	5.85 ± 0.76, 0.951	2.42 ± 0.41, 0.757	2.16 ± 0.44, 0.637	4.58 ± 0.74, 0.624	26.26 ± 3.63, 0.316
High school	1.75 ± 0.31	4.21 ± 0.56	5.88 ± 0.78	2.44 ± 0.41	2.19 ± 0.45	4.62 ± 0.76	26.80 ± 3.92
University and college	1.75 ± 0.32	4.22 ± 0.57	5.87 ± 0.81	2.45 ± 0.43	2.21 ± 0.47	4.66 ± 0.79	26.51 ± 3.85
Occupation							
Farmers, workers, unemployed	1.78 ± 0.33, 0.356	4.26 ± 0.58, 0.138	5.93 ± 0.80, 0.235	2.47 ± 0.44, 0.239	2.20 ± 0.50, 0.642	4.67 ± 0.83, 0.313	26.96 ± 3.95, 0.091
Businessmen, employees	1.74 ± 0.32	4.16 ± 0.58	5.81 ± 0.78	2.41 ± 0.40	2.17 ± 0.41	4.57 ± 0.70	26.50 ± 3.54
Leaders, government employees	1.74 ± 0.31	4.19 ± 0.57	5.88 ± 0.77	2.44 ± 0.39	2.20 ± 0.46	4.64 ± 0.74	26.15 ± 4.08
Household income (RMB/month)							
<4,000	1.77 ± 0.33, 0.422	4.25 ± 0.60, 0.173	5.87 ± 0.81, 0.173	2.45 ± 0.43, 0.194	2.20 ± 0.50, 0.568	4.65 ± 0.82, 0.249	26.71 ± 4.15, 0.617
4,000–10,000	1.74 ± 0.31	4.16 ± 0.53	5.86 ± 0.73	2.41 ± 0.39	2.17 ± 0.42	4.60 ± 0.70	26.63 ± 3.30
>10,000	1.74 ± 0.32	4.22 ± 0.61	5.91 ± 0.85	2.48 ± 0.43	2.21 ± 0.46	4.69 ± 0.78	26.59 ± 3.84
Pre-pregnancy BMI (kg/m^2^)							
<24	1.75 ± 0.31, 0.890	4.21 ± 0.56, 0.956	5.88 ± 0.79, 0.604	2.43 ± 0.40, 0.532	2.19 ± 0.45, 0.944	4.62 ± 0.74, 0.833	26.61 ± 3.80, 0.779
≥24	1.76 ± 0.34	4.20 ± 0.62	5.85 ± 0.76	2.46 ± 0.45	2.19 ± 0.47	4.63 ± 0.82	26.51 ± 3.96
Fasting blood glucose							
<5.1 mmol/L	1.76 ± 0.32, 0.525	4.22 ± 0.59, 0.319	5.88 ± 0.81, 0.597	2.45 ± 0.41, 0.302	2.19 ± 0.45, 0.445	4.64 ± 0.76, 0.335	26.59 ± 3.80, 0.971
≥5.1 mmol/L	1.74 ± 0.30	4.16 ± 0.53	5.84 ± 0.71	2.41 ± 0.42	2.16 ± 0.49	4.57 ± 0.81	26.60 ± 3.98
Hypertension							
No	1.75 ± 0.32, 0.871	4.21 ± 0.57, 0.967	5.87 ± 0.79, 0.809	2.44 ± 0.41, 0.851	2.19 ± 0.46, 0.816	4.62 ± 0.76, 0.946	26.59 ± 3.83, 0.859
Yes	1.74 ± 0.34	4.20 ± 0.76	5.91 ± 0.77	2.42 ± 0.41	2.21 ± 0.51	4.63 ± 0.85	26.71 ± 4.13
Extra-sport activities							
No	1.76 ± 0.32, 0.671	4.19 ± 0.59, 0.543	5.84 ± 0.79, 0.243	2.44 ± 0.42, 0.794	2.18 ± 0.44, 0.692	4.62 ± 0.75, 0.956	26.62 ± 3.89, 0.814
Yes	1.75 ± 0.32	4.22 ± 0.56	5.91 ± 0.78	2.43 ± 0.40	2.20 ± 0.48	4.62 ± 0.77	26.55 ± 3.77
Pre-pregnancy smoking							
No	1.75 ± 0.32, 0.678	4.21 ± 0.58, 0.763	5.87 ± 0.78, 0.387	2.44 ± 0.41, 0.504	2.18 ± 0.44, 0.401	4.60 ± 0.72, 0.576	26.60 ± 3.85, 0.430
Yes	1.81 ± 0.29	4.13 ± 0.55	6.18 ± 0.99	2.56 ± 0.60	2.22 ± 0.52	4.70 ± 0.89	25.24 ± 2.06
Passive smoking during pregnancy							
No	1.75 ± 0.32, 0.957	4.20 ± 0.58, 0.424	5.88 ± 0.80, 0.936	2.42 ± 0.39, 0.076	2.18 ± 0.44, 0.401	4.60 ± 0.72, 0.576	26.60 ± 3.85, 0.430
Yes	1.75 ± 0.33	4.24 ± 0.57	5.87 ± 0.73	2.49 ± 0.47	2.22 ± 0.52	4.70 ± 0.89	25.24 ± 2.06
Pre-pregnancy drinking							
No	1.75 ± 0.32, 0.741	4.19 ± 0.57, 0.058	5.87 ± 0.79, 0.387	2.42 ± 0.40, 0.001	2.18 ± 0.45, 0.025	4.59 ± 0.74, 0.003	26.56 ± 3.79, 0.577
Yes	1.77 ± 0.31	4.34 ± 0.59	5.95 ± 0.80	2.64 ± 0.48	2.31 ± 0.50	4.93 ± 0.88	26.84 ± 4.31
Dietary pattern							
Vegetarian diet	1.76 ± 0.33, 0.063	4.24 ± 0.54, 0.412	5.85 ± 0.85, 0.820	2.42 ± 0.42, 0.124	2.18 ± 0.41, 0.889	4.59 ± 0.73, 0.429	26.97 ± 4.20, 0.036
Meat diet	1.71 ± 0.31	4.16 ± 0.53	5.87 ± 0.73	2.40 ± 0.37	2.18 ± 0.45	4.59 ± 0.72	26.02 ± 3.99
Mixed diet	1.78 ± 0.32	4.22 ± 0.62	5.89 ± 0.80	2.47 ± 0.44	2.20 ± 0.48	4.66 ± 0.80	26.79 ± 3.50
Conception							
Natural conception	1.75 ± 0.32, 0.438	4.20 ± 0.58, 0.536	5.86 ± 0.79, 0.042	2.44 ± 0.42, 0.929	2.19 ± 0.46, 0.790	4.62 ± 0.76, 0.821	26.52 ± 3.77, 0.075
Assisted reproduction	1.79 ± 0.31	4.25 ± 0.56	6.10 ± 0.73	2.44 ± 0.39	2.20 ± 0.50	4.65 ± 0.76	27.60 ± 4.64
Delivery							
Vaginal birth	1.77 ± 0.31, 0.201	4.23 ± 0.58, 0.416	5.88 ± 0.79, 0.797	2.43 ± 0.42, 0.899	2.16 ± 0.44, 0.236	4.59 ± 0.74, 0.392	26.38 ± 3.64, 0.208
Cesarean	1.74 ± 0.32	4.19 ± 0.57	5.87 ± 0.78	2.44 ± 0.41	2.21 ± 0.47	4.64 ± 0.77	26.75 ± 3.98

In terms of dietary patterns, significant differences (26.97 ± 4.20%, 26.02 ± 3.99%, 26.79 ± 3.50%) in the SP/AP ratio of newborns were observed among three groups: vegetarian, meat, and mixed diets (*p* < 0.05). There was no statistically significant difference between the vegetarian diet group and the mixed diet group (*p* > 0.05), but there were significant differences between the other groups (*p* < 0.05). For conception methods, wave V absolute latency (5.86 ± 0.79 ms) in natural conception was significantly lower than that (6.10 ± 0.73 ms) in assisted reproduction (*p* = 0.042), which indicated that assisted reproduction may prolong the auditory conduction function of the fetus (Table 3).

#### 3.1.4 Association of maternal SF and Hb, neonatal characteristics with auditory physiological indicators

The maternal SF (*r* = −0.272 to −0.103, *p* < 0.01), Hb (*r* = −0.304 to −0.229, *p* < 0.01), and neonatal SF (*r* = −0.471 to −0.344, *p* < 0.01) were significantly negatively correlated with auditory physiological indicators in newborns, indicating a close negative correlation between maternal iron nutrition status and the development of neonatal auditory function (Table 4). There was a weak negative correlation between gestational age and wave I latency (*r* = −0.101, *p* < 0.01).

**Table 4:** Association of maternal SF and Hb, neonatal characteristics with auditory physiological indicators.

Characteristics	Absolute latency (ms) (*r*, *p*)			Interpeak latency (ms) (*r*, *p*)			SP/AP (%) (*r*, *p*)
	Wave I	Wave III	Wave V	I-III	III-V	I-V	
Maternal SF (ms)	−0.272, 0.001	−0.112, 0.003	−0.129, 0.001	−0.110, 0.004	−0.106, 0.005	−0.124, 0.001	−0.103, 0.007
Maternal Hb (ms)	−0.304, 0.001	−0.253, 0.001	−0.264, 0.001	−0.229, 0.001	−0.259, 0.001	−0.278, 0.001	−0.244, 0.001
Gestational age (week)	−0.101, 0.008	−0.050, 0.184	−0.030, 0.426	−0.025, 0.518	0.025, 0.515	0.028, 0.465	−0.009, 0.807
Birth weight (kg)	−0.041, 0.280	−0.055, 0.144	0.042, 0.272	−0.007, 0.862	−0.063, 0.095	−0.042, 0.264	0.012·, 0.753
Apgar score	−0.016, 0.665	−0.033, 0.383	−0.033, 0.384	0.003, 0.945	−0.029, 0.446	0.019, 0.617	−0.021, 0.579
Neonatal SF (ms)	−0.446, <0.001	−0.344, <0.001	−0.467, <0.001	−0.384, <0.001	−0.438, <0.001	−0.471, <0.001	−0.426, <0.001

Hb, Hemoglobin; SF, Serum ferritin.

#### 3.1.5 Correlation analysis between characteristics, including maternal and neonatal factors, for auditory physiological indicators in newborns

To further control the influence of confounding factors and avoid collinearity, the correlation analysis was conducted on potential covariates before constructing the GLM. There was a positive correlation between birth weight and extra-sport activities during pregnancy (*r* = 0.446, *p* < 0.05), as well as a positive correlation between maternal Hb and SF (*r* = 0.441, *p* < 0.001). Maternal SF was significantly positively correlated with the modes of conception (*r* = 0.306, *p* < 0.05) and delivery (*r* = 0.288, *p* < 0.05). Maternal Hb was positively correlated with the mode of conception (*r* = 0.267, *p* < 0.05). Neonatal SF was positively correlated with maternal SF (*r* = 0.184, *p* < 0.001) and Hb (*r* = 0.279, *p* < 0.001) (Table 5).

**Table 5:** Correlation coefficient matrix of factors related to auditory physiological indicators in newborns.

Factors	Age	Occupation	Income	Extra-sport	Smoking	Drinking	Dietary	Maternal SF	Maternal Hb	Conception	Delivery	Gestational age	Birth weight	Neonatal SF
Age	1.000													
Occupation	0.075	1.000												
Income	0.093	−0.095^*^	1.000											
Extra-sport	0.018	0.014	0.064	1.000										
Passive smoking	0.028	−0.095^*^	−0.057	0.038	1.000									
Drinking	0.011	0.045	0.047	0.033	0.126^**^	1.000								
Dietary pattern	0.077	0.011	0.054	0.042	0.077	0.034	1.000							
Maternal SF	0.265	−0.019	−0.034	0.292	0.270	0.228	0.290	1.000						
Maternal Hb	0.278	−0.073	−0.036	0.216	0.263	0.240	0.242	0.441^***^	1.000					
Conception	0.060	0.048	0.043	0.064	0.020	0.036	0.027	0.306^*^	0.267^*^	1.000				
Delivery	0.217^***^	0.036	0.051	0.022	0.003	0.017	0.085	0.288^*^	0.278	0.066	1.000			
Gestational age	0.110	0.088	0.085	0.148	0.064	0.083	0.089	0.324	0.279	0.132	0.075	1.000		
Birth weight	0.190	−0.089^**^	−0.134^***^	0.446^*^	0.059	0.005	0.072	0.038	−0.093^*^	0.474	−0.014	0.030	1.000	
Neonatal SF	0.005	0.030	0.013	0.055^*^	0.060^***^	0.034^***^	0.043	0.184^***^	0.279^***^	0.050	0.024	−0.006	0.048	1.000

*p < 0.05, **p < 0.01, ***p < 0.001.

#### 3.1.6 Multivariate analysis of maternal factors on absolute latency (waves I, III, and V)

Based on the univariate analysis, a multiple linear regression model was constructed for wave I latency. Variables with a *p-*value < 0.25 in univariate analysis were included in the multivariate model, which included dietary pattern (*p* = 0.063), mode of delivery (*p* = 0.201), and statistically significant indicators: gestational age (*r* = −0.101, *p* = 0.008), maternal SF (*r* = −0.272, *p* < 0.001), and Hb (*r* = −0.304, *p* < 0.001). Considering the significant correlation between SF and Hb (*p* < 0.001), to avoid collinearity issues, this study adopted a stepwise modeling strategy to construct three regression models: Model 1 included maternal SF, Model 2 included maternal Hb, and Model 3 included neonatal SF (Table 6). In Model 1 (including maternal SF), the meat diet (*β* = −0.060, *p* = 0.023), gestational age (*β* = −0.015, *p* = 0.011), and SF (*β* = −0.007, *p* < 0.001) showed significant negative effects on wave I latency. In Model 2 (including maternal Hb), the meat diet (*β* = −0.069, *p* = 0.010), gestational age (*β* = −0.018, *p* = 0.003), and Hb (*β* = −0.007, *p* < 0.001) also showed significant negative effects. In Model 3 (including neonatal SF), the meat diet (*β* = −0.054, *p* = 0.027), gestational age (*β* = −0.017, *p* = 0.002), and SF (*β* = −0.006, *p* < 0.001) also showed significant negative effects. These results indicated that, whether SF and Hb were examined separately, dietary patterns, gestational age, and iron metabolism indicators had independent predictive effects on wave I latency (Table 6).

**Table 6:** Univariate analysis of related factors on wave I latency by multiple generalized linear models.

Factors	*β* (95% CI)	Wald Chi-Square	*p*
Model 1			
Dietary pattern			
Vegetarian diet	−0.005 (−0.064, 0.053)	0.030	0.862
Meat diet	−0.060 (−0.112, −0.008)	5.167	0.023
Mixed diet			
Conception	0.037 (−0.009, 0.083)	2.511	0.113
Gestational age	−0.015 (−0.027, −0.003)	6.426	0.011
Maternal SF	−0.007 (−0.009, −0.005)	54.556	<0.001
Model 2			
Dietary pattern			
Vegetarian diet	−0.006 (−0.065, 0.053)	0.040	0.841
Meat diet	−0.069 (−0.121, −0.016)	6.596	0.010
Mixed diet			
Conception	0.038 (−0.008, 0.084)	2.605	0.107
Gestational age	−0.018 (−0.030, −0.006)	8.991	0.003
Maternal Hb	−0.007 (−0.009, −0.005)	42.850	<0.001
Model 3			
Dietary pattern			
Vegetarian diet	−0.004 (−0.058, 0.051)	0.020	0.886
Meat diet	−0.054 (−0.103, −0.006)	4.885	0.027
Mixed diet			
Conception	0.025 (−0.017, 0.068)	1.371	0.242
Gestational age	−0.017 (−0.028, −0.006)	9.657	0.002
Neonatal SF	−0.006 (−0.007, −0.005)	173.956	<0.001

The results were consistent with the analysis results of the wave I latency model mentioned, indicating that there was an independent and consistent negative correlation between iron metabolism indicators and the latency of auditory evoked potentials (Table 7).

**Table 7:** Univariate analysis of related factors on wave III latency by multiple generalized linear models.

Factors	*β* (95%CI)	Wald Chi-Square	*P*
Model 1			
Occupation			
Farmers, workers, unemployed	0.081 (−0.029, 0.192)	2.085	0.149
Businessmen, employees	−0.031 (−0.140, 0.179)	0.297	0.586
Leaders, government employees			
Pre-pregnancy drinking	−0.139 (−0.281, 0.003)	3.674	0.055
Gestational age	−0.012 (−0.034, 0.009)	1.240	0.266
Birth weight	0.000 (0.000, 0.000)	2.889	0.089
Maternal SF	−0.005 (−0.008, −0.002)	8.190	0.004
Model 2			
Occupation			
Farmers, workers, unemployed	0.098 (−0.011, 0.208)	3.088	0.079
Businessmen, employees	0.002 (−0.108, 0.112)	0.001	0.971
Leaders, government employees			
Pre-pregnancy drinking	−0.141 (−0.282, 0.000)	3.857	0.050
Gestational age	−0.015 (−0.036, 0.006)	1.865	0.172
Birth weight	0.000 (0.000, 0.000)	1.938	0.164
Maternal Hb	−0.009 (−0.013, −0.005)	18. 910	<0.001
Model 3			
Occupation			
Farmers, workers, unemployed	0.068 (−0.036, 0.173)	1.648	0.199
Businessmen, employees	−0.001 (−0.103, 0.106)	0.001	0.978
Leaders, government employees			
Pre-pregnancy drinking	−0.125 (−0.259, 0.010)	3.315	0.069
Gestational age	−0.014 (−0.035, 0.006)	1.883	0.170
Birth weight	0.000 (0.000, 0.000)	1.694	0.193
Neonatal SF	−0.008 (−0.010, −0.006)	89.388	<0.001

Assisted reproduction (*β* = 0.243, *p* = 0.047) showed significant positive effects on wave V latency in Model 1. Iron metabolism indicators showed significant negative effects (Table 8).

**Table 8:** Univariate analysis of related factors on wave V latency by multiple generalized linear models.

Factors	*β* (95%CI)	Wald Chi-square	*p*
Model 1			
Occupation			
Farmers, workers, unemployed	0.061 (−0.090, 0.211)	0.624	0.430
Businessmen, employees	−0.078 (−0.229, 0.072)	1.038	0.308
Leaders, government employees			
Extra-sport activities	−0.078 (−0.196, −0.039)	1.704	0.192
Conception	0.243 (0.003, 0.483)	3.951	0.047
Maternal SF	−0.008 (−0.013, −0.004)	11.911	0.001
Model 2			
Occupation			
Farmers, workers, unemployed	0.092 (−0.057, 0.241)	1.464	0.226
Businessmen, employees	−0.025 (−0.176, 0.125)	0.109	0.741
Leaders, government employees			
Extra-sport activities	−0.085 (−0.201, 0.031)	2.070	0.150
Conception	−0.214 (−0.451, 0.023)	3.130	0.077
Maternal Hb	−0.015 (−0.021, −0.010)	29.927	<0.001
Model 3			
Occupation			
Farmers, workers, unemployed	0.044 (−0.098, 0.185)	0.370	0.543
Businessmen, employees	−0.027 (−0.170, 0.115)	0.142	0.706
Leaders, government employees			
Extra-sport activities	−0.040 (−0.151, 0.070)	0.511	0.475
Conception	−0.186 (−0.412, 0.040)	2.605	0.107
Neonatal SF	−0.012 (−0.014, −0.010)	102.858	<0.001

#### 3.1.7 Multivariate analysis of maternal factors on interpeak latency and SP/AP

Based on the univariate analysis, the regression model for interpeak latency wave I–III included variables with potential impact: maternal age (*p* = 0.167), occupation (*p* = 0.239), household income (*p* = 0.194), passive smoking during pregnancy (*p* = 0.076), pre-pregnancy drinking (*p* < 0.001), and dietary pattern (*p* = 0.124). At the same time, iron metabolism indicators significantly correlated with interpeak latency wave I-III.

In Model 1 and Model 3, pre-pregnancy drinking (*β* = 0.206, 0.198, *p* < 0.001) was associated with longer latency wave I-III, while SF (*β* = −0.003, *p* = 0.008; *β* = −0.006, *p* < 0.001) showed a negative effect. Model 2 showed that unemployment (*β* = 0.135, *p* = 0.035) was associated with prolonged latency, whereas moderate household income (CNY¥4,000–10,000 /month) (*β* = −0.105, *p* = 0.032) had a shortening effect. Pre-pregnancy drinking (*β* = 0.198, *p* < 0.001) was positively associated with prolonged latency. Iron metabolism indicators showed significant negative effects (Table 9).

**Table 9:** Univariate analysis of maternal factors on interpeak latency wave I-III by multiple generalized linear models.

Factors	*β* (95%CI)	Wald Chi-square	*p*
Model 1			
Age	−0.055 (−0.134, 0.025)	1.836	0.175
Occupation			
Farmers, workers, unemployed	0.111 (−0.016, 0.237)	2.950	0.086
Businessmen or employees	0.028 (−0.067, 0.123)	0.338	0.561
Leaders, government employees			
Household income (RMB/month)			
<4,000	−0.112 (−0.242, 0.019)	2.822	0.093
4,000–10,000	−0.095 (−0.191, 0.002)	3.712	0.054
>10,000			
Passive smoking during pregnancy	−0.047 (−0.120, 0.026)	1.613	0.204
Pre-pregnancy drinking	0.206 (0.104, 0.307)	15.722	<0.001
Dietary pattern			
Vegetarian diet	−0.040 (−0.114, 0.033)	0.282	0.388
Meat diet	−0.052 (−0.121, 0.017)	2.159	0.142
Mixed diet			
Maternal SF	−0.003 (−0.006, −0.001)	6.931	0.008
Model 2			
Age	−0.044 (−0.128, 0.040)	1.045	0.307
Occupation			
Farmers, workers, unemployed	0.135 (0.009, 0.261)	4.427	0.035
Businessmen or employees	0.055 (−0.041, 0.150)	1.259	0.262
Leaders, government employees			
Household income (RMB/month)			
<4,000	−0.128 (−0.257, 0.002)	3.727	0.054
4,000–10,000	−0.105 (−0.200, −0.009)	4.603	0.032
>10,000			
Passive smoking during pregnancy	−0.044 (−0.116, 0.029)	1.391	0.238
Pre-pregnancy drinking	0.208 (0.107, 0.309)	16.305	<0.001
Dietary pattern			
Vegetarian diet	−0.034 (−0.112, 0.044)	0.743	0.389
Meat diet	−0.056 (−0.124, 0.013)	2.558	0.110
Mixed diet			
Maternal Hb	−0.006 (−0.009, −0.003)	15.007	<0.001
Model 3			
Age	−0.056 (−0.135, 0.023)	1.938	0.164
Occupation			
Farmers, workers, unemployed	0.092 (−0.026, 0.209)	2.347	0.126
Businessmen or employees	0.048 (−0.041, 0.137)	1.116	0.291
Leaders, government employees			
Household income (RMB/month)			
<4,000	−0.095 (−0.216, 0.207)	2.343	0.126
4,000–10,000	−0.087 (−0.177, 0.003)	3.624	0.057
>10,000			
Passive smoking during pregnancy	−0.026 (−0.095, 0.042)	0.581	0.446
Pre-pregnancy drinking	0.198 (0.104, 0.293)	16.868	<0.001
Dietary pattern			
Vegetarian diet	−0.033 (−0.106, 0.040)	0.773	0.379
Meat diet	−0.044 (−0.109, 0.020)	1.839	0.175
Mixed diet			
Neonatal SF	−0.006 (−0.008, −0.005)	115.064	<0.001

For interpeak latency wave III–V, pre-pregnancy drinking prolonged interpeak latency (*β* = 0.010, 0.125, *p* < 0.05), and iron metabolism indicators shortened interpeak intervals (Table 10). For interpeak latency wave I-V, the two models are consistent with the results of interpeak latency wave III-V (Table 11). For SP/AP, maternal SF (*β* = −0.032, *p* = 0.007) and Hb (*β* = −0.064, *p* < 0.001) showed significant negative correlation with SP/AP (Table 12).

**Table 10:** Univariate analysis of maternal factors on interpeak latency wave III-V by multiple generalized linear models.

Factors	*β* (95%CI)	Wald Chi-Square	*p*
Model 1			
Pre-pregnancy drinking	0.125 (0.012, 0.239)	4.715	0.030
Delivery	−0.043 (−0.111, 0.025)	1.561	0.211
Birth weight	0.000 (0.000, 0.000)	2.790	0.095
Maternal SF	−0.004 (−0.006, −0.001)	6.955	0.008
Model 2			
Pre-pregnancy drinking	0.125 (0.013, 0.236)	4.814	0.028
Delivery	−0.039 (−0.106, 0.028)	1.322	0.250
Birth weight	0.000 (0.000, 0.000)	1.783	0.182
Maternal Hb	−0.009 (−0.012, −0.005)	27.869	<0.001
Model 3			
Pre-pregnancy drinking	0.110 (0.007, 0.212)	4.418	0.036
Delivery	−0.054 (−0.116, 0.007)	3.008	0.083
Birth weight	0.000 (0.000, 0.000)	1.799	0.180
Neonatal SF	−0.008 (−0.010, −0.007)	164.555	<0.001

**Table 11:** Univariate analysis of maternal factors on interpeak latency wave I-V by multiple generalized linear models.

Factors	*β* (95%CI)	Wald Chi-Square	*p*
Model 1			
Household income (RMB/month)			
<4,000	−0.019 (−0.168, 0.130)	0.063	0.802
4,000–10,000	−0.101 (−0.244, 0.042)	1.902	0.168
>10,000			
Passive smoking during pregnancy	−0.081 (−0.216, 0.054)	1.388	0.239
Pre-pregnancy drinking	0.311 (0.123, 0.500)	10.459	0.001
Maternal SF	−0.007 (−0.012, −0.003)	9.810	0.002
Model 2			
Household income (RMB/month)			
<4,000	−0.015 (−0.162, 0.133)	0.038	0.846
4,000–10,000	−0.086 (−0.227, 0.055)	1.416	0.234
>10,000			
Passive smoking during pregnancy	−0.074 (−0.206, 0.059)	1.179	0.278
Pre-pregnancy drinking	0.314 (0.128, 0.500)	10.835	0.001
Maternal Hb	−0.017 (−0.020, −0.009)	29.254	<0.001
Model 3			
Household income (RMB/month)			
<4,000	−0.023 (−0.156, 0.109)	0.117	0.732
4,000–10,000	−0.069 (−0.196, 0.058)	1.136	0.287
>10,000			
Passive smoking during pregnancy	−0.027 (−0.147, 0.093)	0.198	0.657
Pre-pregnancy drinking	0.293 (0.125, 0.461)	11.705	0.001
Neonatal SF	−0.015 (−0.017, −0.013)	195.039	<0.001

**Table 12:** Univariate analysis of maternal factors on SP/AP by multiple generalized linear models.

Factors	*β* (95%CI)	Wald Chi-Square	P
Model 1			
Occupation			
Farmers, workers, unemployed	0.831 (−0.097, 1.566)	4.929	0.026
Businessmen or employees	0.352 (−0.379, 1.083)	0.891	0.345
Leaders, government employees			
Dietary pattern			
Vegetarian diet	0.206 (−0.524, 0.936)	0.306	0.580
Meat diet	−0.638 (−1.279, 0.015)	3.665	0.056
Mixed diet			
Conception	−1.057 (−2.227, 0.113)	3.136	0.077
Delivery	−0.249 (−0.819, 0.321)	0.733	0.392
Maternal SF	−0.032 (−0.055, −0.009)	7.404	0.007
Model 2			
Occupation			
Farmers, workers, unemployed	0.966 (0.236, 1.695)	6.728	0.009
Businessmen or employees	0.582 (−0.149, 1.313)	2.432	0.119
Leaders, government employees			
Dietary pattern			
Vegetarian diet	0.215 (−0.507, 0.938)	0.341	0.560
Meat diet			
Mixed diet	−0.676 (−1.316, 0.036)	4.285	0.038
Conception	−0.938 (−2.098, 0.221)	2.516	0.113
Delivery	−0.220 (−0.785, 0.344)	0.586	0.444
Maternal Hb	−0.064 (−0.091, −0.037)	21.774	<0.001
Model 3			
Occupation			
Farmers, workers, unemployed	0.741 (0.074, 1.409)	4.740	0.029
Businessmen or employees	0.612 (−0.054, 1.279)	3.244	0.072
Leaders, government employees			
Dietary pattern			
Vegetarian diet	0.255 (−0.409, 0.919)	0.567	0.451
Meat diet	−0.527 (−1.115, 0.062)	3.079	0.079
Mixed diet			
Conception	−0.684 (−1.750, 0.382)	1.581	0.209
Delivery	−0.366 (−0.884, 0.153)	1.911	0.167
Neonatal SF	−0.068 (−0.079, −0.057)	153.978	<0.001

AP, Action potential; SP, Summating potential.

#### 3.1.8 Mediation analyses of the associations between maternal SF and auditory physiological indicators by neonatal SF

To investigate the potential effects of maternal SF (X) on auditory physiological indicators (Y), we introduced neonatal SF as a mediator variable (M) and included it in the structural equation model for analysis. When evaluating various auditory physiological indicators, we comprehensively considered the influence of other relevant factors and controlled for statistically significant indicators in GLM as covariates. The mediation analy**ses** were conducted using Model 4 of the PROCESS macro program in SPSS, and based on Hayes’ Bootstrap method, the mediating effects of neonatal SF between maternal SF and auditory physiological indicators were validated and quantified.

**Figure 1:**
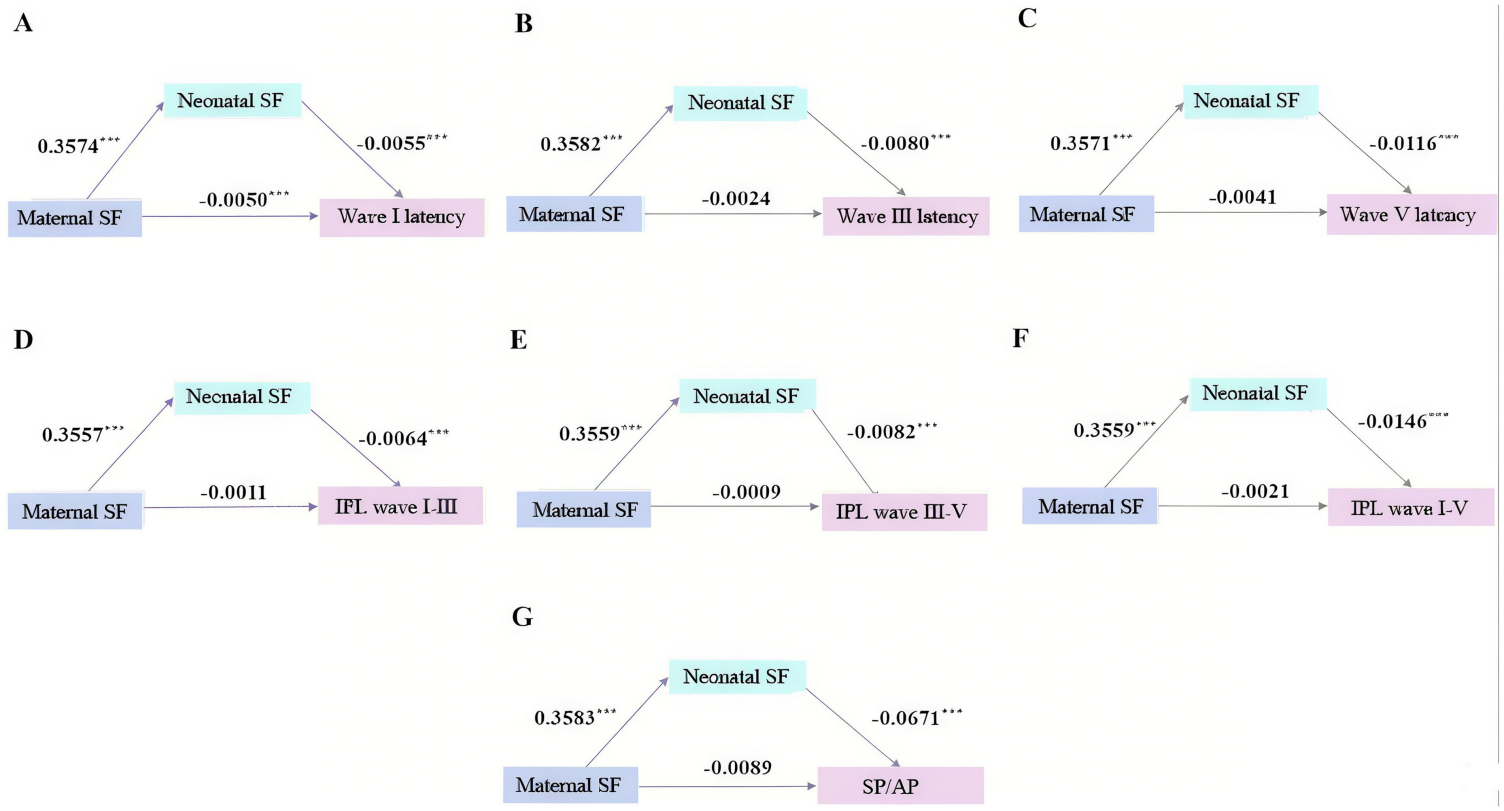
Path coefficient diagrams of maternal and neonatal serum ferritin (SF) affecting auditory physiological indicators. **(A)** for wave I latency, **(B)** for wave Ⅲ latency, **(C)** for wave Ⅴ latency, **(D)** for interpeak latency wave (IPL) wave I–Ⅲ, **(E)** for IPL wave Ⅲ–Ⅴ, **(F)** for IPL wave Ⅰ–Ⅴ, **(G)** for summating potential (SP)/action potential (AP).

The path coefficients of neonatal SF (M) between maternal SF (X) and auditory physiological indicators (Y) are shown in Figure 1. Maternal SF only had a statistically significant direct effect on wave I latency in auditory physiological indicators and had no significant direct effect on other auditory physiological indicators. Maternal SF had a significant effect on neonatal SF, with a correlation coefficient of 0.3582, and when all factors are restricted (*p* < 0.001), the correlation coefficient ranged from 0.3557 to 0.3583. The regression coefficients of neonatal SF on auditory physiological indicators of ABR ranged from −0.0055 to 0.0146, and the regression coefficient on SP/AP (%) of ECochG was −0.0671 (*p* < 0.001).

The total effect of maternal SF on neonatal auditory physiological indicators showed statistical significance (*p* < 0.01), indicating that maternal SF had an important role in the overall impact on auditory function. However, further analysis revealed that the direct effect of maternal SF on auditory physiological indicators was only significant at wave I latency (*p* < 0.001), while the direct effects on other indicators (wave III latency, wave V latency, interpeak latency wave, and SP/AP ratio) did not show statistical significances (*p* > 0.05), which indicated that the impact of maternal SF on neonatal auditory function can be primarily achieved through indirect pathways.

The Bootstrap method was used to examine the mediating effect of neonatal SF (M) between maternal SF (X) and auditory physiological indicators (Y). The results showed that the upper limits of the 95% confidence interval (ULCI) and lower limits of the 95% confidence interval (LLCI) did not include 0, indicating that neonatal SF played a significant mediating role in the associations between maternal SF and auditory physiological indicators. Specifically, for wave I latency, the direct effect and mediating effect accounted for 28.57 and 71.43% of the total effect, respectively. The mediating effects of neonatal SF on wave III and wave V latency were 54.72 and 50%, respectively. In addition, the mediating effects of neonatal SF on interpeak latency wave and SP/AP ratio were 67.65% for wave I–III, 76.32% for wave III–V, 71.23% for wave I–V, and 73.03% for SP/AP (Table 13).

**Table 13:** The total and direct effects of maternal SF on auditory physiological indicators, as well as the mediating effect of neonatal SF.

Auditory physiological indicators	Effect type	Value	SE	*p*	LLCI	ULCI	Effect size (%)
Wave I Latency	Total effect	−0.0070	0.0009	<0.001	−0.0088	−0.0051	100.00
	Direct effect	−0.0050	0.0000	<0.001	−0.0067	−0.0033	71.43
	Indirect effect	−0.0020	0.0004	<0.001	−0.0028	−0.0012	28.57
Wave III latency	Total effect	−0.0053	0.0018	0.0030	−0.0087	−0.0018	100.00
	Direct effect	−0.0024	0.0017	0.1619	−0.0057	−0.0010	45.28
	Indirect effect	−0.0029	0.0006	<0.001	−0.0043	−0.0017	54.72
Wave V latency	Total effect	−0.0082	0.0024	0.0007	−0.0129	−0.0035	100.00
	Direct effect	−0.0041	0.0023	0.0760	−0.0086	−0.0004	50.00
	Indirect effect	−0.0041	0.0010	<0.001	−0.0062	−0.0025	50.00
IPL wave I-III	Total effect	−0.0034	0.0013	0.0068	−0.0059	−0.0009	100.00
	Direct effect	−0.0011	0.0012	0.3380	−0.0035	−0.0012	32.35
	Indirect effect	−0.0023	0.0005	<0.001	−0.0033	−0.0014	67.65
IPL wave III-V	Total effect	−0.0038	0.0014	0.0069	−0.0066	−0.0010	100.00
	Direct effect	−0.0009	0.0013	0.5010	−0.0034	0.0017	23.68
	Indirect effect	−0.0029	0.0006	<0.001	−0.0043	−0.0018	76.32
IPL wave I-V	Total effect	−0.0073	0.0023	0.0016	−0.0119	−0.0028	100.00
	Direct effect	−0.0021	0.0021	0.3124	−0.0062	0.0020	28.77
	Indirect effect	−0.0052	0.0011	<0.001	−0.0075	−0.0032	71.23
SP/AP	Total effect	−0.0330	0.0118	0.0053	−0.0561	−0.0099	100.00
	Direct effect	−0.0089	0.0109	0.4135	−0.0303	0.0125	26.97
	Indirect effect	−0.0241	0.0051	<0.001	−0.0346	−0.0148	73.03

IPL, interpeak latency wave; AP, Action potential; SP, Summating potential; SF, serum ferritin; SE, standard error; LLCI, lower limit of confidence interval; ULCI, Upper limit of confidence interval.

#### 3.1.9 Mediation analyses of the associations between maternal Hb and auditory physiological indicators by neonatal SF

**Figure 2:**
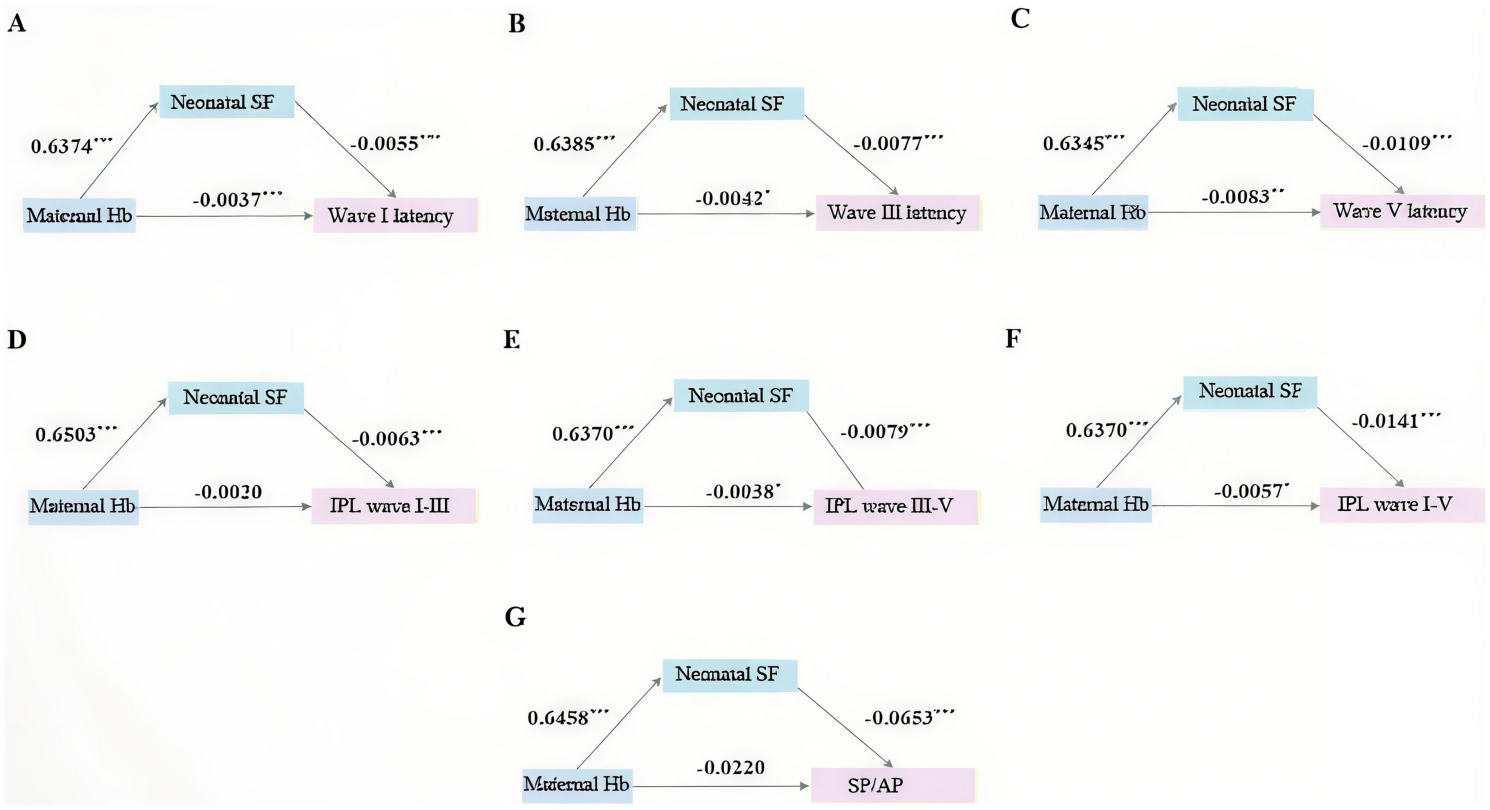
Path coefficient diagrams of maternal hemoglobin (Hb) and neonatal serum ferritin (SF) affecting auditory physiological indicators. **(A)** for wave I latency, **(B)** for wave Ⅲ latency, **(C)** for wave Ⅴ latency, **(D)** for interpeak latency wave (IPL) wave I–Ⅲ, **(E)** for IPL wave Ⅲ–Ⅴ, **(F)** for IPL wave Ⅰ–Ⅴ, **(G)** for summating potential (SP)/action potential (AP).

The path coefficients of neonatal SF between maternal Hb and auditory physiological indicators are shown in Figure 2. Maternal Hb had a statistically significant direct effect only on wave I, III, and V latency (*p* < 0.001, *p* < 0.05, *p* < 0.01), and a statistically significant direct effect on wave III–V and I–V latency (*p* < 0.05), whereas the direct effects on other auditory physiological indicators were not significant (*p* > 0.05). Maternal Hb had a significant impact on neonatal SF. When all factors are not restricted, the correlation coefficient is 0.2764. The path coefficient ranged from 0.6345 to 0.6503, with all *p-*values of < 0.001. The regression coefficients of neonatal SF on auditory physiological indicators were as follows: wave I latency (*β* = 0.0055, *p* < 0.001), wave III latency (*β* = 0.0077, *p* < 0.001), wave V latency (*β* = 0.0109, *p* < 0.001), wave I–III latency (*β* = −0.0063, *p* < 0.001), III-V (*β* = −0.0079, *p* < 0.001), and I–V (*β* = −0.0141, *p* < 0.001). The regression coefficient for SP/AP of the cochlear electroencephalogram was −0.0653 (*p* < 0.001).

ULCI and LLCI for the mediating effect of neonatal SF did not include 0, indicating that Hb not only had a direct effect on various indicators of hearing testing but also exerted a significant mediating effect through SF.

The direct effects of maternal Hb on the waves I-III latency and SP/AP were not significant (*p* > 0.05); however, the direct effects on other indicators were significant (*p* < 0.05). The mediating effect of neonatal SF between maternal Hb and neonatal auditory physiological indicators was significant, with an effect size ranging from 45.39 to 67.21% (Table 14).

**Table 14:** The total and direct effects of maternal Hb on auditory physiological indicators, as well as the mediating effect of neonatal SF.

Auditory physiological indicators	Effect type	Value	SE	*p*	LLCI	ULCI	Effect size (%)
Wave I latency	Total effect	−0.0072	0.0011	<0.001	−0.0094	−0.0050	100.00
	Direct effect	−0.0037	0.0011	<0.001	−0.0057	−0.0016	51.39
	Indirect effect	−0.0035	0.0005	<0.001	−0.0046	−0.0025	48.61
Wave III latency	Total effect	−0.0091	0.0021	<0.001	−0.0132	−0.0051	100.00
	Direct effect	−0.0042	0.0020	0.0391	−0.0082	−0.0002	46.15
	Indirect effect	−0.0049	0.0008	<0.001	−0.0067	−0.0034	53.85
Wave V latency	Total effect	−0.0152	0.0028	<0.001	−0.0207	−0.0098	100.00
	Direct effect	−0.0083	0.0027	0.0026	−0.0137	−0.0029	54.61
	Indirect effect	−0.0069	0.0013	<0.001	−0.0096	−0.0045	45.39
IPL wave I-III	Total effect	−0.0061	0.0015	<0.001	−0.0089	−0.0032	100.00
	Direct effect	−0.0020	0.0014	0.1694	−0.0048	−0.0008	32.79
	Indirect effect	−0.0041	0.0006	<0.001	−0.0054	−0.0029	67.21
IPL wave III-V	Total effect	−0.0088	0.0016	<0.001	−0.0120	−0.0056	100.00
	Direct effect	−0.0038	0.0015	0.0148	−0.0068	−0.0007	43.18
	Indirect effect	−0.0050	0.0009	<0.001	−0.0068	−0.0035	56.82
IPL wave I-V	Total effect	−0.0147	0.0027	<0.001	−0.0200	−0.0094	100.00
	Direct effect	−0.0057	0.0025	0.0232	−0.0106	−0.0008	38.78
	Indirect effect	−0.0090	0.0014	<0.001	−0.0120	−0.0064	61.22
SP/AP	Total effect	−0.0642	0.0137	<0.001	−0.0911	−0.0373	100.00
	Direct effect	−0.0220	0.0131	0.0930	−0.0478	−0.0037	34.27
	Indirect effect	−0.0422	0.0063	<0.001	−0.0551	−0.0307	65.73

IPL, interpeak latency wave; AP, Action potential; SP, Summating potential; SF, serum ferritin; SE, standard error; LLCI, lower limit of confidence interval; ULCI, Upper limit of confidence interval.

### 3.2 Animal results

#### 3.2.1 Maternal ID disturbed growth, iron status, and reduced ABR wave I peak amplitude of young mice

The ID mouse model was successfully established by feeding a low-iron diet for 3 weeks. ID dams and pups exhibited significantly lower SF than those in the control group (*p* < 0.05), while the red cell distribution width (RDW) indicator of those in the ID group was significantly higher than that of the control group (*p* < 0.05). These changes in indicators were consistent with the typical characteristics of mild ID. There were no statistical differences in body weight, Hb, SI, and Hct between the two groups (*p* > 0.05) (Supplementary Table S2; Supplementary Figure S1).

As the intervention time extended to 24 days, ID dams exhibited obvious localized hair loss characteristics in the back and the outer side of their hind legs. For 30 days, the hair loss symptoms showed a progressive worsening trend (Supplementary Figure S2B). This temporal surface change may be closely related to the abnormal nutritional metabolism of skin appendages caused by ID. Another interesting phenomenon was that the feces of ID dams were lighter in color and had a looser structure (Supplementary Figure S2C). Iron is an essential element for heme synthesis. Reduced fecal bile production causes feces to become lighter in color during ID, which indicates that ID may further affect the normal function of the intestine and lead to changes in fecal characteristics. There were no statistical differences in food intake and weight gain during pre-pregnancy, pregnancy, and postpartum stages between the two groups (Supplementary Figures S2D,E).

To evaluate peripheral and central auditory functions, we used ABR hearing thresholds in response to various stimuli. ABR wave I amplitude analysis included the amplitude of wave I, time of peak, and SPL of the click between the two groups at varying tone pip frequencies (kHz). On PND 21, the average ABR thresholds of young ID mice were not significantly different compared to control at 4, 8, 16, and 32 kHz (Supplementary Figure S3, *p* > 0.05). ID pups showed significantly reduced wave I amplitudes despite no significant differences in ABR thresholds, a pattern compatible with subclinical synaptic or neural alterations rather than the overt threshold shift (Supplementary Figure S3, *p* > 0.05), which indicated that maternal ID might induce early electrophysiological changes in offspring with hidden hearing loss (HHL).

#### 3.2.2 Maternal ID reduced IHC ribbon synapses in young mice

**Figure 3:**
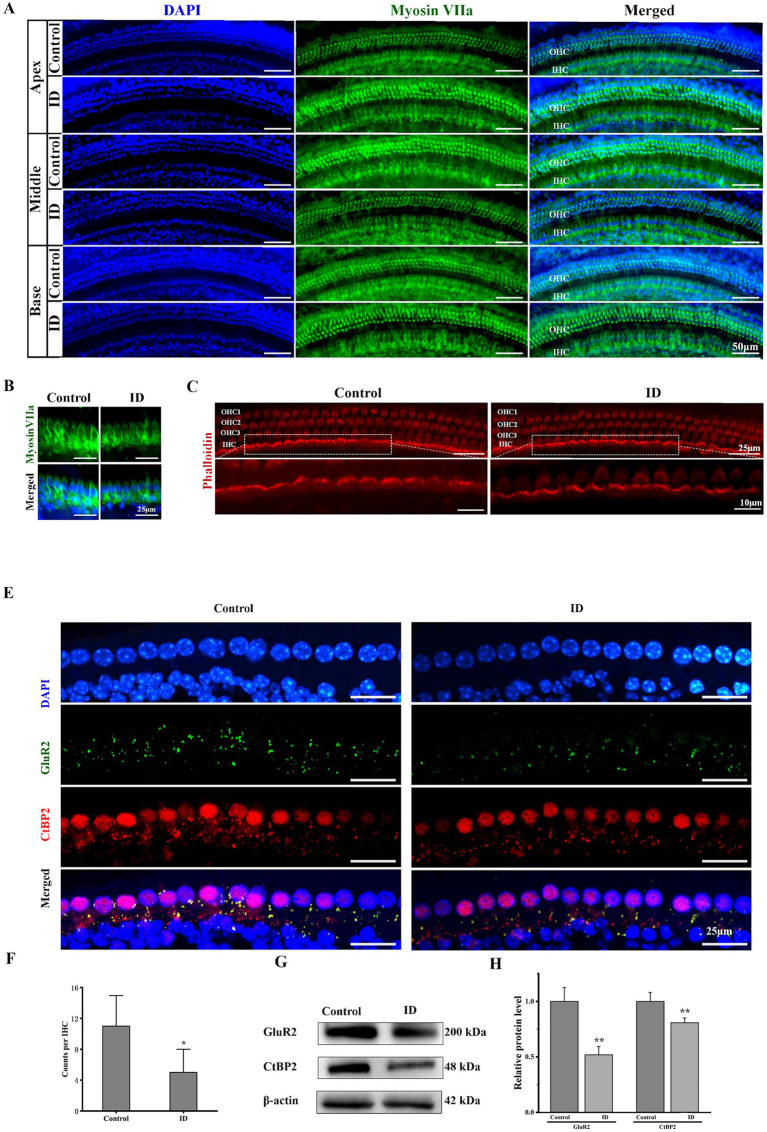
Immunofluorescence staining and protein expression of cochlear basement membrane of young mice on PND 14. **(A)** OHC and IHC immunofluorescence staining with DAPI labeling the cell nucleus (blue) and Myosin VII labeling HC (green). The apex, middle, and base areas with a magnification of 200 × and a scale bar of 50 μm. **(B)** IHC immunofluorescence staining with DAPI-labeled cell nucleus (blue), Myosin VII-labeled IHC (green), magnified 400 ×, and scale bar of 25 μm. **(C)** Phalloidin labeled HC cell cytoskeleton (red), magnified 400 × and scale bar of 25 μm. **(D)** Phalloidin labeled cilia of basement membrane (green), magnified 400 × and scale bar of 25 μm. **(E)** Anti-CtBP2 labeled presynaptic membrane (red), anti-GluR2 labeled postsynaptic membrane (green), intact synaptic (yellow), magnified 1,000 ×, and scale bar of 25 μm. **(F)** Synaptic count statistical analysis, * *p* < 0.05; **(G)** GluR2 and CtBP2 protein band diagrams; **(H)** Statistical analysis of GluR2 and CtBP2 proteins, ^**^ *p* < 0.01 (*n* = 4).

The morphology (Figures 3A–D) showed that OHCs (3 rows) and IHCs (1 row) were present in the apex, middle, and base areas of the cochlea of young mice on PND 14. The regular arrangement showed intact ciliary structure and no significant cell loss or disordered arrangement. However, the number of ribbon synapses in the IHC region of ID young mice was significantly reduced compared to control mice (*p* < 0.001), and multiple synapses exhibited structural damage characteristics. The yellow fusion signal formed by membrane pairing before and after synapses was significantly weakened (Figures 3E,F, *p* < 0.05). We further confirmed that the protein expressions of CtBP2 and GluR2 in ID young mice were significantly decreased compared to control mice (Figures 3G,H, *p* < 0.05). Overall, although the morphology of hair cells remained intact, maternal ID could impair IHC synaptic structure and downregulate synaptic-related protein expression. This result suggested that prenatal ID may mainly affect auditory function by disrupting the synaptic function of the offspring cochlea rather than directly damaging the hair cell structure.

#### 3.2.3 Maternal ID impaired the mitochondria of the supporting cells of the greater epithelial ridge (GER) in young mice

**Figure 4:**
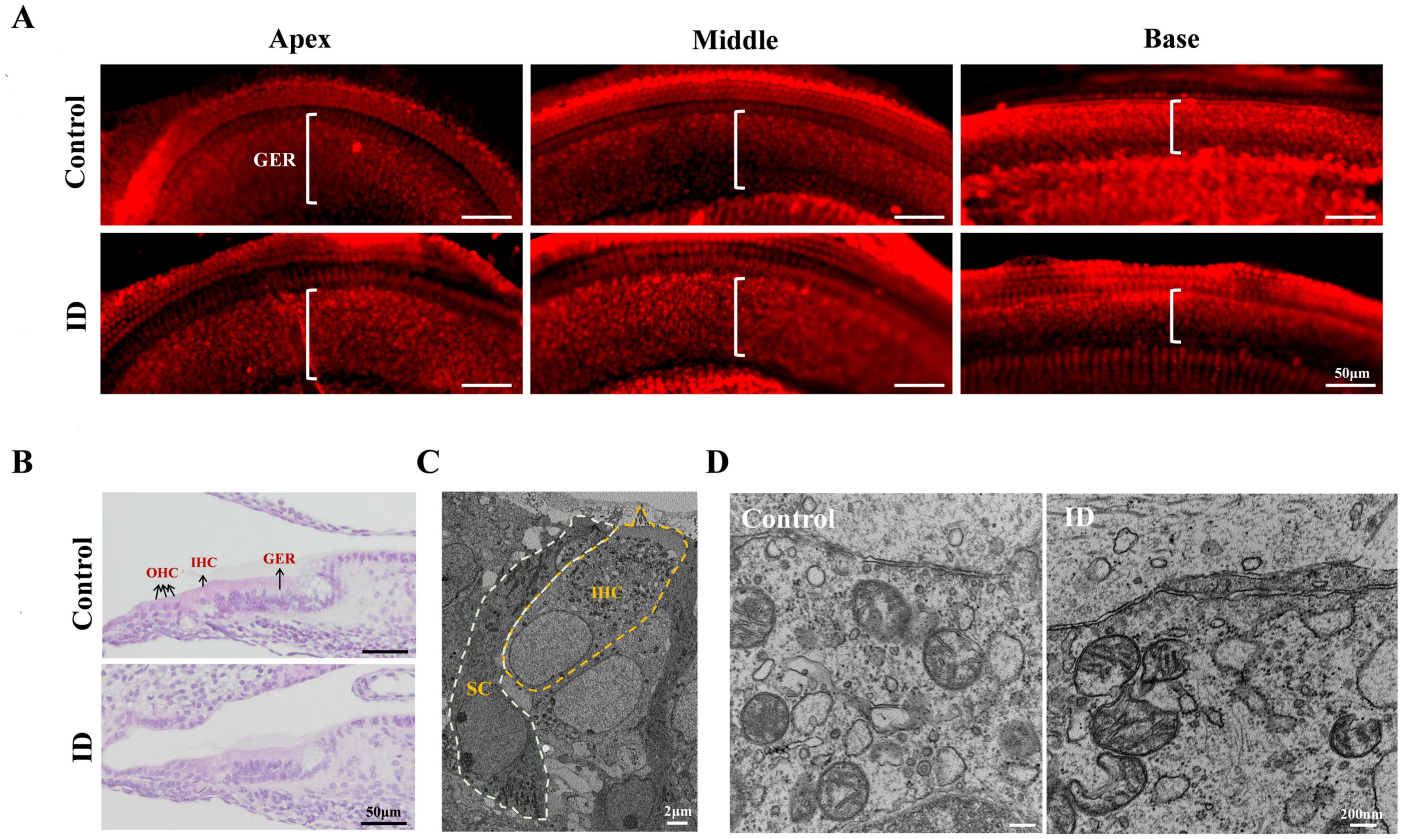
Morphological analysis of greater epithelial ridge (GER) and supporting cells in young mice on PND 2. **(A)** Immunofluorescence staining of the mouse cochlear basement membrane, Sox2 (red) labelling supporting cells within HC and GER. The white boxes represent the GER structure, magnified 200 ×, and a scale bar of 50 μm. **(B)** HE staining with arrows indicating OHC, IHC, and GER structures, magnified 200 × and scale bar of 50 μm; **(C)** Supporting cells (within the white box) and IHC (within the yellow box) under a projection electron microscope, magnified 4,000 × and scaled at a scale of 2 μm; **(D)** Representative scanning electron microscopy showing morphology of mitochondria, magnified 50,000 × and scale bar of 200 μm.

The study found that the functional activity of the large epithelial ridge was the strongest on PND 2 and then gradually degenerated and completely disappeared on PND 14. On PND 2, the number of supporting cells decreased from apex to base of the cochlear basal membrane, which was located on GER (Figure 4A). The supporting cells gradually changed from high columnar to short columnar, indicating that the cochlea of mice was still in the developmental stage after birth. There was no significant difference in the morphology and number of GER between the two groups (Figures 4A,B). SEM showed that the dependent structure of IHC and supporting cells remained intact between the two groups (Figures 4C,D); however, the mitochondria in the ID group exhibited abnormal morphology, such as vacuolization, indicating that ID primarily affected mitochondrial function rather than the GER structure.

**Figure 5:**
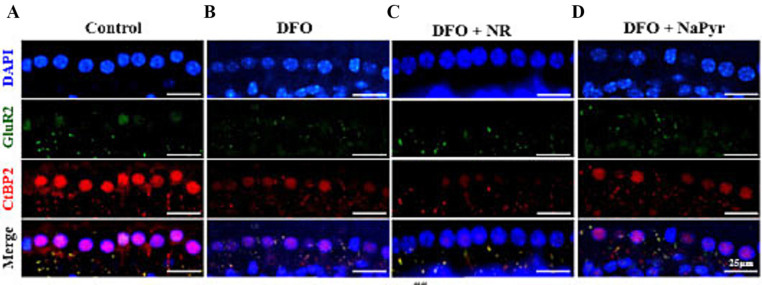
Tetranitroblue tetrazolium chloride (NBT) staining and cochlear energy metabolism in young mice on PND 7. **(A)** The cochlear basement membrane stained with NBT (blue), with a magnification of 400 × and a scale bar of 25 μm. **(B)** The blue precipitate gray value analysis (NBT staining) representing the basement membrane, IHC represents the IHC and SC dependent areas, **p < 0.01. **(C)** NAD + in the cochlea; **(D)** NAD+/NADH ratio in the cochlea, **p < 0.01.

On PND 7, ID young mice showed that the succinate dehydrogenase (SDH) activity in the cochlear HC cytoplasm and IHC-supporting cells junction was significantly reduced compared to control mice using tetranitroblue tetrazolium chloride staining (Figures 5A,B). Moreover, the levels of NAD^+^ in the cochlea and the NAD^+^/NADH ratio were significantly decreased in ID young mice on PND 2–14 (Figures 5C,D, *p* < 0.05), which collectively indicated that ID decreased SDH activity by damaging mitochondrial structure, which in turn caused NAD^+^ metabolism disorders and ultimately affected cochlear energy metabolism processes.

### 3.3 *In vitro* experiments

#### 3.3.1 DFO-induced ID in supporting cells

**Figure 6:**
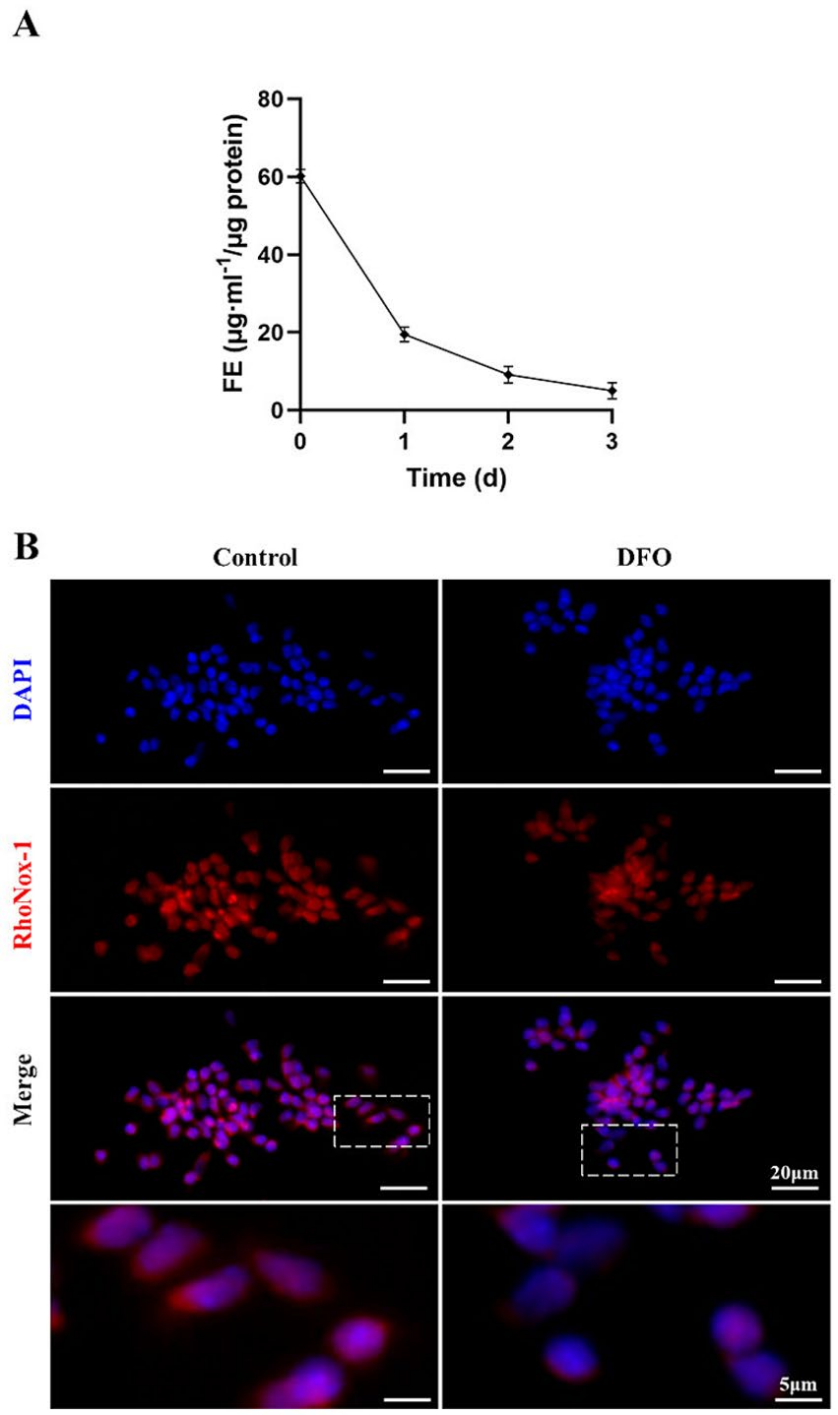
Fe2 + levels in cochlear explant supporting cells after desferrioxamine (DFO) treatment. **(A)** Changes in ferritin levels after DFO chelation. **(B)**. Fe2 + levels in the supporting cells of cochlear explants were measured using RhoNox-1 labeled Fe2 + (red) and DAPI labeled cell nuclei (blue), with a magnification of 200 × and a scale bar of 5 μm.

After enzymatic digestion to remove connective tissue (Supplementary Figure S4), the connection structure between IHCs and supporting cells was effectively dissociated, and the boundaries were clear and distinguishable, which provided good experimental conditions for independently obtaining IHCs and supporting cells. *In vitro* ID was obtained by incubation with DFO in the cochlear explants, and ferritin decreased in a time-dependent manner during DFO incubation at a concentration of 100 μmol/L. After 1 day of culture, the supporting cells were isolated. The Fe^2+^ in the DFO group decreased, which induced ID in the supporting cells (see Figure 6).

#### 3.3.2 ID disrupted energy metabolism, redox homeostasis, and mitochondrial function in supporting cells

**Figure 7:**
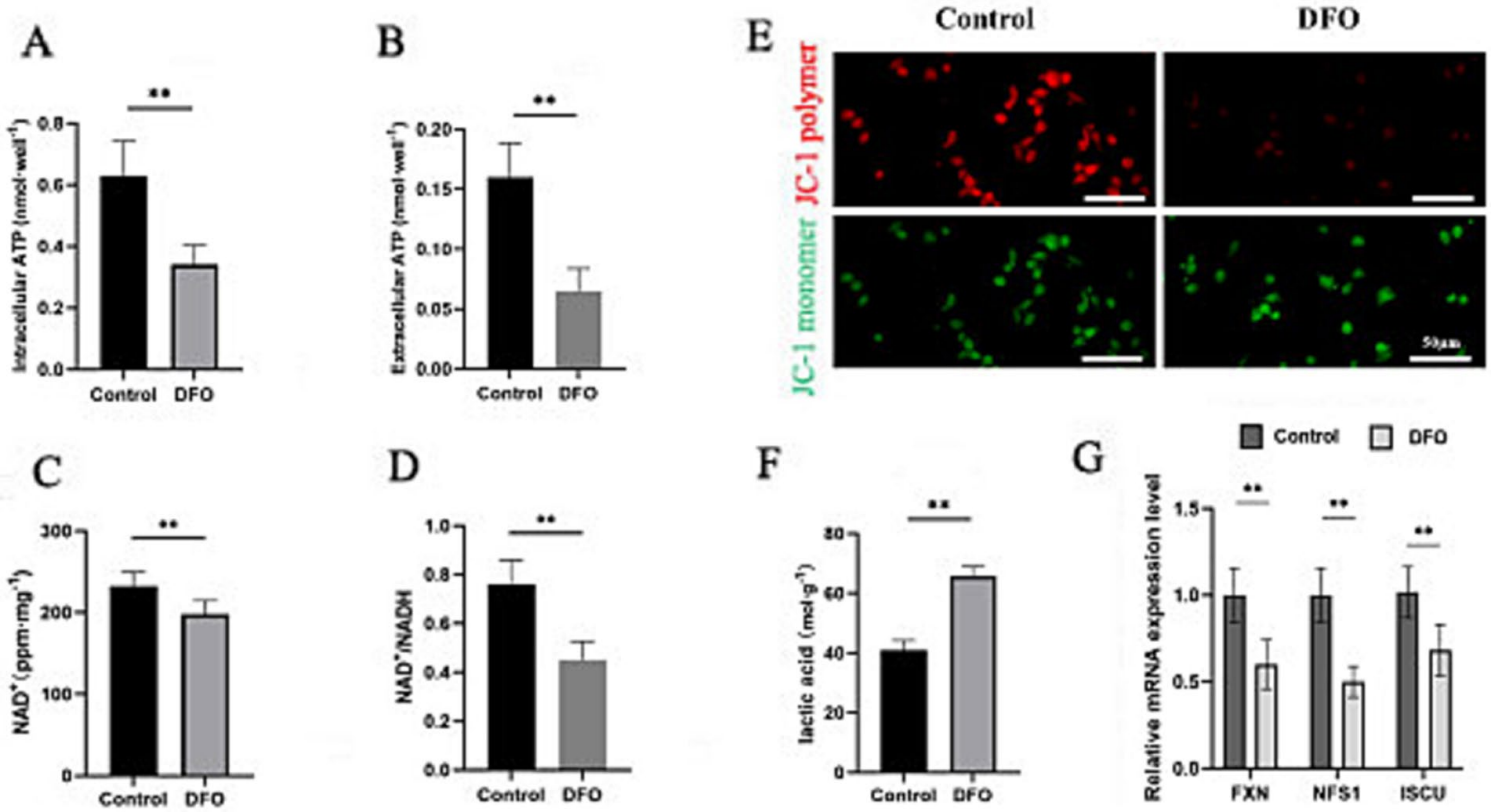
Energy metabolism in supporting cells. **(A)** Intracellular ATP content; **(B)** Extracellular ATP content, **p < 0.01 (*n* = 10). **(C)** NAD + content **(D)** NAD+/NADH ratio **p < 0.01 (*n* = 4). **(E)** JC-1 staining analysis of mitochondrial membrane potential changes, with a scale bar of 50 μm, JC-1 polymer (red) and JC-1 monomer (green). **(F)** Cellular lactate content, **p < 0.01 (*n* = 6). **(G)** mRNA expression levels of iron sulfur cluster (ISC) related molecules, FXN, frataxin; NFS1, cysteine desulfurase; ISCU, iron–sulfur cluster assembly scaffold protein. **p < 0.01 (*n* = 6).

The intracellular ATP content in the supporting cells of the DFO group was significantly decreased compared to control (Figures 7A,B, *p* < 0.01), indicating that ATP synthesis in the supporting cells was significantly inhibited under ID induced by DFO. The extracellular ATP content in the culture medium also decreased synchronously, which was consistent with the decreasing trend of intracellular ATP content. ID may decrease its own ATP production, which in turn leads to a decrease in the ATP content being released into the extracellular space. These findings were consistent with impaired energy-metabolism–related readouts in DFO-treated supporting cells, as reflected by reductions in intracellular and extracellular ATP levels. DFO chelation treatment significantly reduced the intracellular NAD^+^ content (Figures 7C,D, *p* < 0.01), while the NAD^+^/NADH ratio also showed a similar degree of decrease (*p* < 0.01), indicating that DFO treatment may alter the intracellular NAD^+^ homeostasis by directly affecting NAD^+^ biosynthesis or promoting NAD^+^ degradation. Iron is a cofactor for many functional proteins in cells, and a decrease in mitochondrial iron levels affects the normal function of the tricarboxylic acid cycle and electron transport chain. After 24 h of DFO treatment, the DFO group showed a decrease in mitochondrial polymers and an increase in monomers, indicating a significant decrease in membrane potential (Figure 7E). After 24 h of DFO treatment, the intracellular lactate content was significantly higher than that of the control (Figure 7F, *p* < 0.01), indicating that ID in supporting cells may interfere with cellular energy metabolism pathways, leading to intensified glycolysis and the production of large amounts of lactate. Frataxin (FXN), cysteine disulfide (NFS1), and iron–sulfur cluster assembly scaffold protein are key molecules involved in iron–sulfur cluster (ISC) synthesis. The mRNA expression levels of ISC synthesis-related molecules FXN, NFS1, and iron sulfur cluster assembly scaffold protein were reduced in the DFO group (Figure 7G, *p* < 0.01).

#### 3.3.3 ID reduced the activity of key enzymes of supporting cells

**Figure 8:**
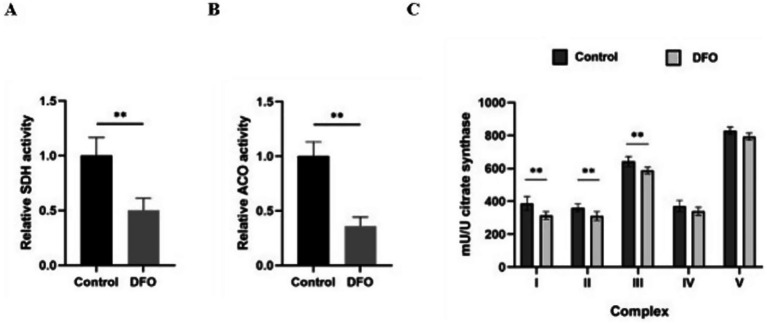
Enzyme activity detection. **(A)** Succinate dehydrogenase (SDH) activity, **p < 0.01 (*n* = 6). **(B)** Aconitinase (ACO) enzyme activity, **p < 0.01 (*n* = 6). **(C)** The enzyme activity of oxidative phosphorylation complex I-V (Complex I-V) **p < 0.01 (*n* = 6).

The activities of SDH and aconitinase (ACO) in the DFO group were significantly reduced (Figure 8, *p* < 0.01), indicating that ID not only affected enzyme function dependent on iron sulfur clusters but also interfered with the normal operation of the tricarboxylic acid (TCA) cycle. The activity of mitochondrial respiratory chain complexes was also widely inhibited, with a significant decrease in the activity of complexes I, II, and III. ID in cells can seriously damage the electron transport chain function and lead to a decrease in energy metabolism efficiency. These data revealed that DFO may interfere with energy metabolism processes, particularly inhibiting early stages of the mitochondrial electron transport chain, leading to disruptions in cellular energy metabolism and redox imbalances.

#### 3.3.4 ID induced by DFO chelation downregulated pyruvate expression through targeted energy metabolomics analysis

Supplementary Figure S5 shows the quality control analysis data of the samples. The 80% reference line further confirmed the overall data quality reliability.

**Figure 9:**
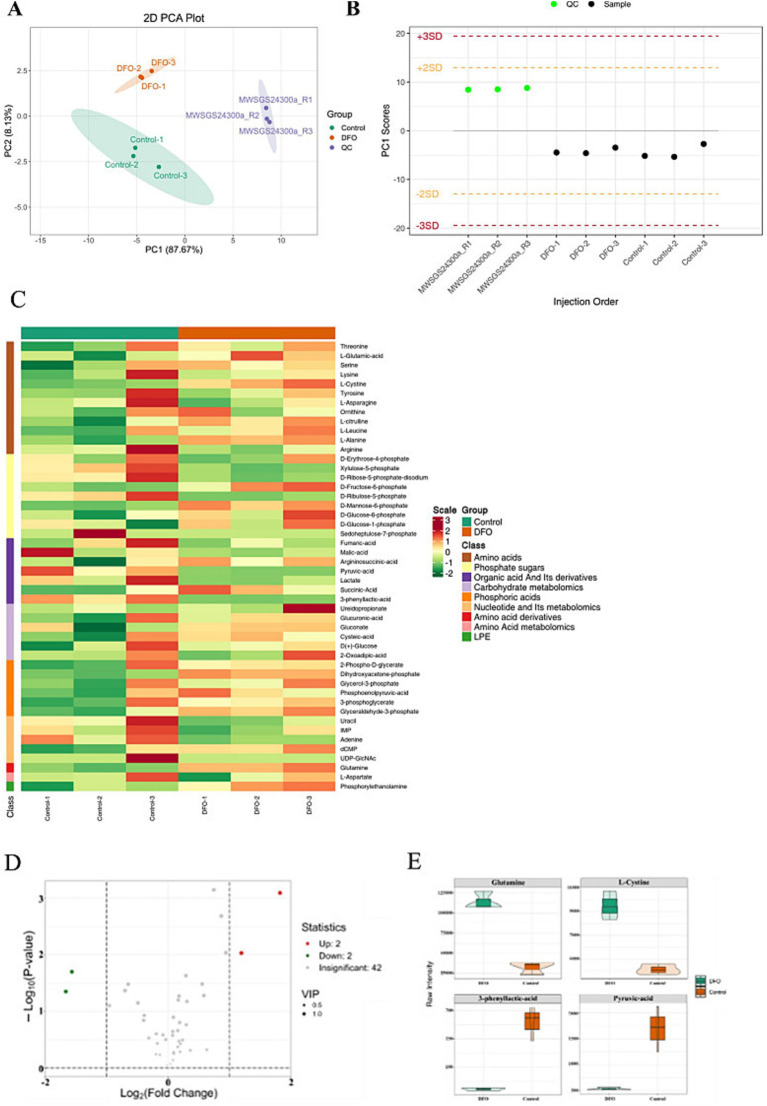
Targeted energy metabolomics analysis. **(A)** Score plot of principal component analysis of mass spectrometry data for samples, **(B)** PC1 Control chart of principal component 1 for the overall sample. **(C)** Clustering diagram for the overall sample. **(D)** Volcanic diagram of differential metabolites, **(E)** Violin plot of differential metabolites.

The principal component analysis showed that principal component 1 and principal component 2 explained 87.67 and 18.3% of the total variation of the data, respectively (Figure 9A). There was a significant separation trend in the principal component space, indicating significant differences in the metabolic profiles between the two groups. The principal component 1 scores of the quality control samples remained stable within the ± 2SD range (Figure 9B), confirming the high reliability and stability of the experimental data. The heat map analysis (Figure 9C) showed significant differences in metabolite expression profiles between the two groups. In the control group, metabolites such as threonine and serine showed high expression characteristics (red), while their expression levels decreased in the DFO group (green). The DFO group showed specific high expression of metabolites such as glycine-3-phosphate and L-leucine (red), which may reflect the disorder of metabolic regulation under ID. Further differential metabolite analysis (Figure 9D) showed a total of four significant metabolite changes (VIP > 1), among which glutamine and L-Cystine were significantly upregulated in the DFO group, while DL-3-phenyllactic acid and pyruvic acid were significantly downregulated. The violin plot analysis intuitively displayed the intergroup distribution characteristics of these four differential metabolites (Figure 9E). The expression levels of glutamine and cysteine in the DFO group were significantly higher than those in the control group, and their distribution density and median were significantly increased. However, DL-3-phenyllactic acid and pyruvic acid exhibited opposite regulatory patterns. These results collectively indicated that ID may affect the normal physiological functions of cells by interfering with pathways related to amino acid metabolism and energy metabolism.

Supplementary Table S3 shows differential metabolite screening, including the four compounds selected and their changes. The content of pyruvic acid significantly decreased (*p* = 0.045), the content of DL-3-phenyllactic acid also showed a decreasing trend (*p* = 0.020), L-Cystine increased (*p* = 0.009), and glutamine significantly increased (*p* < 0.001). Supplementary Figure 6 shows correlation analysis on metabolites with significant differences identified through screening. The Pearson correlation coefficients between pyruvic acid and DL-3-phenyllactic acid, L-cysteine, and glutamine were 0.96, −0.90, and −0.86, respectively. The differential metabolite chord plot (Supplementary Figure S6B) provides a more intuitive display of the Log_2FC values, source classification, and correlation coefficients of four metabolites, revealing the differential expression of metabolites and their interrelationships between the two groups.

#### 3.3.5 DFO chelation decreased the Ca^2+^ activity and synapses of IHC, and sodium pyruvate partially restored them

In the detection of glycolysis pathways in supporting cells chelated by DFO, the levels of alpha-D-glucose-1-phosphate, alpha-D-glucose, D-fructose-6-phosphate, glycerone phosphate, glycerate-2-phosphate, phosphoenolpyruvate, and L-lactate did not show significant changes (Supplementary Figure S7). In the detection of the tricarboxylic acid cycle and oxidative phosphorylation process in DFO-treated supporting cells through KEGG pathways, multiple enzymes were downregulated (Supplementary Figures S8, S9).

Targeted energy metabolomics analysis showed that DFO induced ID, significantly downregulating intracellular pyruvate levels, affecting the overall energy metabolism pathway. This result suggested that iron chelator treatment may specifically affect the pyruvate metabolism pathway, while other detected intermediate metabolites in the glycolysis pathway were not significantly disrupted. These data indicate that ID may affect cellular energy supply by selectively regulating the pyruvate metabolic pathway, while upstream metabolic reactions in glycolysis remain relatively stable.

Fluo-4 a.m. was used to label the changes in intracellular Ca^2+^ concentration in IHCs. The P2X receptor antagonist PPADS significantly weakened the fluorescence signal, while exogenous ATP significantly increased the fluorescence intensity, confirming that the ATP-P2X receptor pathway promoted Ca^2+^ influx into IHCs. The Ca^2+^ level in the DFO group decreased significantly compared to control, while supplementation with metabolic intermediates, sodium pyruvate (NaPyr) or nicotinamide riboside (NR) effectively increased intracellular Ca^2+^ concentration. Energy metabolism was associated with the regulation of Ca^2+^ homeostasis in IHCs by promoting ATP production (Supplementary Figure S10).

**Figure 10:**
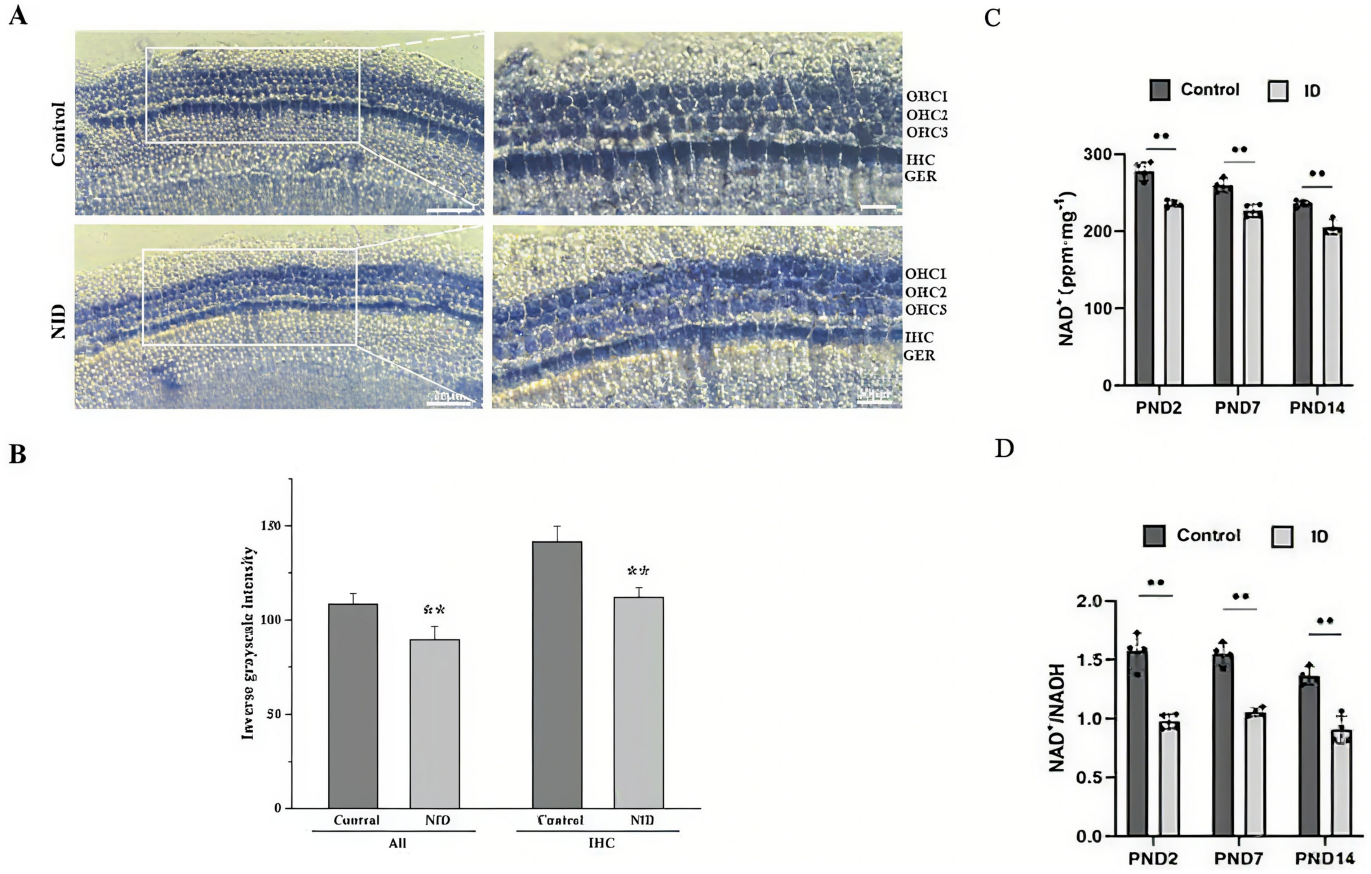
Synaptic status in different treatment groups. **(A)** Immunofluorescence staining of cochlear explants, with DAPI labeling of the nucleus (blue), anti-CtBP2 labeling of the presynaptic membrane (red), and anti-GluR2 labeling of the postsynaptic membrane (green). The intact synapse merges into a yellow color, with a magnification of 1,000 × and a scale bar of 25 μm (*n* = 3). **(B)** Band diagrams of GluR2 and CtBP2 proteins before and after synapses (*n* = 3). **(C)** GluR2 protein statistical analysis, **p < 0.01 (*n* = 3). **(D)** Pattern diagram of the effects of different treatments on synapses.

Considering that NaPyr and NR have better cellular stability, this study established the DFO + NaPyr, DFO + NR combined treatment group to supplement the pyruvate metabolism pathway (Figure 10). Compared with the control group, the number of synapses between hair cells and spiral ganglion neurons in the DFO treatment group was significantly reduced, and the change in GluR2 was more significant, indicating that DFO treatment caused significant damage to synaptic structure and reduced synaptic connections. In the DFO + NR and DFO + NaPyr treatment groups, the number of synapses significantly increased compared to the DFO group. Supplementation with the energy intermediates NR and NaPyr may restore energy metabolism and mitigate the impact of ID-induced energy metabolism obstruction on synapses, suggesting that metabolic supplementation may partially attenuate DFO-associated synaptic alterations in this experimental system.

## 4 Discussion

We used an observational mother–infant retrospective study and a novel dietary restriction model to show the effect of maternal iron nutrition status on the development of the auditory system in newborns and explored potential mechanisms through *in vitro* experiments. This study suggests that prenatal ID may impair the auditory physiological maturity of newborns. The electrophysiological testing provided experimental support for a prolongation of the latency and interphase of ABR waves, as well as an abnormal increase in the SP/AP ratio of ECochG. By establishing an animal model, we observed no significant changes in the growth and development indicators and ABR detection threshold of ID young mice. However, ID prolonged the latency and decreased the amplitude of wave I, which was consistent with the electrophysiological changes observed in human population studies. Combining morphological and molecular biology analysis, ID during pregnancy reduced the number of IHC ribbon synapses, accompanied by mitochondrial dysfunction features such as decreased succinate dehydrogenase activity and abnormal NAD^+^/NADH ratio. ID may affect the energy metabolism process of the cochlea, leading to insufficient ATP synthesis in the large epithelial crest supporting cells during development and interfering with the normal depolarization of IHC and the precise regulation of synaptic connections. In particular, the downregulation of synaptic key proteins such as CtBP2 and GluR2 may constitute an important molecular mechanism through which prenatal ID affects the maturation of neonatal auditory function. These results provided experimental support for the proposed mechanism linking nutritional factors to sensory nervous system development.

IHCs exhibit typical spontaneous calcium-dependent action potentials in the first week after birth, and the self-generating activity actively participates in and regulates the developmental process of the cochlea before the formal establishment of auditory function (26). This study found that ID decreased the pyruvate level, which in turn significantly inhibited the tricarboxylic acid cycle efficiency and ATP synthesis ability through a targeted metabolomics analysis. As a key node molecule in the energy metabolism network, the downregulation of pyruvate concentration was associated with the intracellular Ca^2+^ homeostasis and synaptic transmission efficiency (27). Furthermore, we found that exogenous addition of sodium pyruvate (10 mM) can partially restore the Ca^2+^ fluctuation frequency and synaptic density in ID cells, which provided experimental support for the proposed mechanism of auditory dysfunction caused by ID by interfering with energy metabolism pathways. At the same time, we observed a decrease of approximately 42% in NAD^+^ levels, significantly reducing oxidative phosphorylation efficiency (electron transport chain activity decreased by 35 ± 3.8%), thereby impairing the normal function of cochlear hair cells. These findings were largely in line with existing theories. Iron-dependent enzymes, including NADH dehydrogenase, a key constituent of mitochondrial complex I, may contribute substantially to the maintenance of cellular redox homeostasis (28), which suggests that ID-related mitochondrial dysfunction may serve as a potential molecular basis underlying aberrant auditory system development.

Subsequently, we further explored the potential mediating association of neonatal SF in the relationship between maternal iron nutritional status and offspring auditory function. The results preliminarily suggested that neonatal SF may exert a certain mediating effect linking maternal SF and Hb levels to neonatal auditory function. This study conducted SF testing at the time of newborn birth, which can better represent their iron metabolism level in the uterus. Using changes in wave I latency as an example, we observed that 28.57% were contributed by direct effects (maternal iron directly affecting the auditory pathway), whereas 71.43% were mediated by neonatal SF. A mediation analysis suggested that neonatal SF may statistically account for part of the association between maternal iron status and neonatal auditory indicators. Given the observational design of the human study, these findings should be interpreted as hypothesis-generating rather than evidence of a confirmed causal mediation pathway. WHO points out that the fetus develops rapidly in late pregnancy, and at this time, the demand for iron accounts for more than 80% of the total iron demand throughout pregnancy, as evidenced by the prevalence of ID in all pregnancies (29). Fetal iron nutrition relies entirely on active transmembrane transport mediated by the placenta. Transferrin (Tf) in the maternal circulation binds to transferrin receptor 1 (TFR1) located on the top surface of the syncytiotrophoblast cell layer and is absorbed by the latter (30). Subsequently, the complex formed by Tf and TFR1 undergoes endocytosis, releasing iron ions in the nucleus and ultimately exporting them to the fetal circulatory system through the basal side iron transport protein (Ferroporin) (31). Iron transport not only exists between the mother and the fetus, but also distributes within the fetus. In mammals, including humans, iron is primarily used for the synthesis of Hb in red blood cells, followed by supplying tissues such as the brain, heart, skeletal muscle, and the liver (32, 33). The appropriate gestational age for full-term newborns is approximately 75 mg iron/kg body weight, of which nearly 55 mg/kg is used for red blood cell mass (34). The average concentration of SF is 135 μg/L, and non-heme and non-storage tissues such as the brain account for 8 mg/kg (32, 35). It is speculated that the regulatory mechanism of maternal iron homeostasis may exert potential influences on iron homeostasis in the placenta and fetus, which has been preliminarily verified in mouse models.

This mechanism can protect the placenta and fetus from damage in the event of maternal iron overload and promote the absorption of iron in the placenta (via TFR1) and its transport to the fetus by Ferroporin in the presence of maternal ID, indicating strict regulation of key transport proteins (7, 36). However, when the supply and storage of iron in the diet are already very limited, the abovementioned mechanism may not be sufficient to solve the problem. When the maternal Hb concentration falls below 100 g/L, the entire maternal–fetal system may be affected, causing the fetus to be unable to accumulate enough iron for life after birth. When the maternal ferritin concentration is below 13.4 μg/dL, it is considered a critical point for impaired fetal iron reserves (37). When ferritin levels are below 76 μg/L, newborns may exhibit neurological symptoms (38). When IDA occurs, the level of ferritin in the fetus also decreases (36, 39), which may promote the transfer of iron through the placenta to the fetus and mobilize the fetus’s own iron storage. The decrease in fetal ferritin during IDA can at least be partially achieved through the action of fetal erythroferrite. The adverse effects of postnatal ID on child development have been described in detail, while relatively few studies have focused on the negative effects of prenatal ID, and evaluation of iron status in newborns has rarely been evaluated in postnatal studies. This disparity is largely attributable to the prevailing view that newborns tend to be less susceptible to ID, as fetuses can accumulate iron by consuming maternal iron and typically do not have anemia at birth. The extent to which incidence of postnatal ID is caused by insufficient iron reserves in the fetal period remains unclear, as does the extent to which the symptoms that can be completely or partially attributed to the ID during the fetal period. However, an increasing number of evidence suggests that the relationship between prenatal and postnatal iron status is closer than previously thought (40–42). This study preliminarily investigated the potential association between gestational ID and neonatal auditory development. When compared with other well-documented gestational risk factors, such as tobacco and alcohol exposure, gestational diabetes mellitus, and hypertension, this factor may present certain consistent trends as well as distinctive manifestations. It is speculated that gestational ID may interfere with fetal iron accumulation, which could further exert adverse influences on the physiological state of cochlear supporting cells and potentially induce abnormal energy metabolism and altered synaptic connectivity. Such possible adverse effects share partial similarities with those induced by gestational diabetes-related hyperglycemic microvascular changes (43) and tobacco exposure-mediated vasoconstriction and cochlear hypoxia (44). Nonetheless, their potential influencing pathways may differ. Gestational ID is presumed to be closely linked to the regulation of iron-dependent enzyme activities, while hyperglycemia may exert relevant effects mainly via oxidative stress responses (45), and nicotine may act primarily through nAChR-related pathways (46–48). Clinically, gestational ID tend to induce generalized prolongation of absolute latency and interpeak latency of ABR waveforms, alongside a possible elevation in the SP/AP ratio of ECochG. Its adverse influence on neonatal auditory development might be relatively more evident compared with that of other gestational adverse exposures, including tobacco exposure (49, 50) and gestational hypertension (51). This study preliminarily indicated that neonatal iron status may exert a mediating effect, accounting for approximately 28.57–76.32% in the process of maternal–fetal iron transmission. Such findings may offer a novel perspective to interpret the association between iron metabolism and neonatal auditory function. Relevant animal observations have further suggested that ID tend to impair inner hair cell synapses rather than hair cells, which appeared to differ noticeably from the pathological alterations seen in noise exposure and aging-related experimental models (9).

This study had several limitations. First, this mother–infant observational study was insufficient to establish a causal relationship linking maternal iron status to aberrant auditory maturation. Second, these findings from assumption-based mediation analysis may not fully correspond to biological causal mediation relationships. Third, given the limited clinical generalizability, these findings from animal and *in vitro* experiments may not be directly extrapolated to therapeutic strategies applicable to humans. Fourth, although individuals with overt inflammation were excluded based on hs-CRP detection, residual inflammatory interference with SF levels cannot be entirely ruled out. Fifth, neonatal auditory indicators only reflect short-term physiological maturation. Long-term follow-up data were not available in this study.

## 5 Conclusion

In this study, lower maternal iron-status indicators were associated with less mature neonatal auditory physiological profiles in a mother–infant cohort. Complementary animal and cochlear explant experiments provided experimental evidence linking prenatal ID to altered cochlear energy-metabolism–related readouts and synaptic maturation. These findings support a biologically plausible connection between maternal iron status and early auditory development, while further longitudinal and translational studies are required to clarify causality and clinical implications.

## Data Availability

The original contributions presented in the study are included in the article/Supplementary material, further inquiries can be directed to the corresponding authors.

## Glossary

ABR: auditory brainstem response
ANOVA: analysis of variance
CI: confidence intervals
DFO: desferrioxamine
FXN: Frataxin
GD: gestational day
GLM: generalized linear model
Hb: hemoglobin
HHL: hidden hearing loss
ID: iron deficiency
IDA: iron deficiency anemia
ISC: iron sulfur cluster
NaPyr: sodium pyruvate
NFS1: cysteine disulfide
NR: nicotinamide riboside
SD: standard deviation
SDH: succinate dehydrogenase
SEM: scanning electron microscopy
SF: serum ferritin
SI: serum iron
TCA: tricarboxylic acid
Tf: Transferrin
TFR1: transferrin receptor 1
