# Supplementary figures and images for: Association between maternal iron deficiency and delayed neonatal auditory maturation and altered cochlear synaptic energy metabolism: analysis from a mother–infant observational study, mouse models, and cochlear explants
Source: Front Nutr. 2026 Jun 19;13:1842147. doi: 10.3389/fnut.2026.1842147 (PMC13328176; doi:10.3389/fnut.2026.1842147)

**A**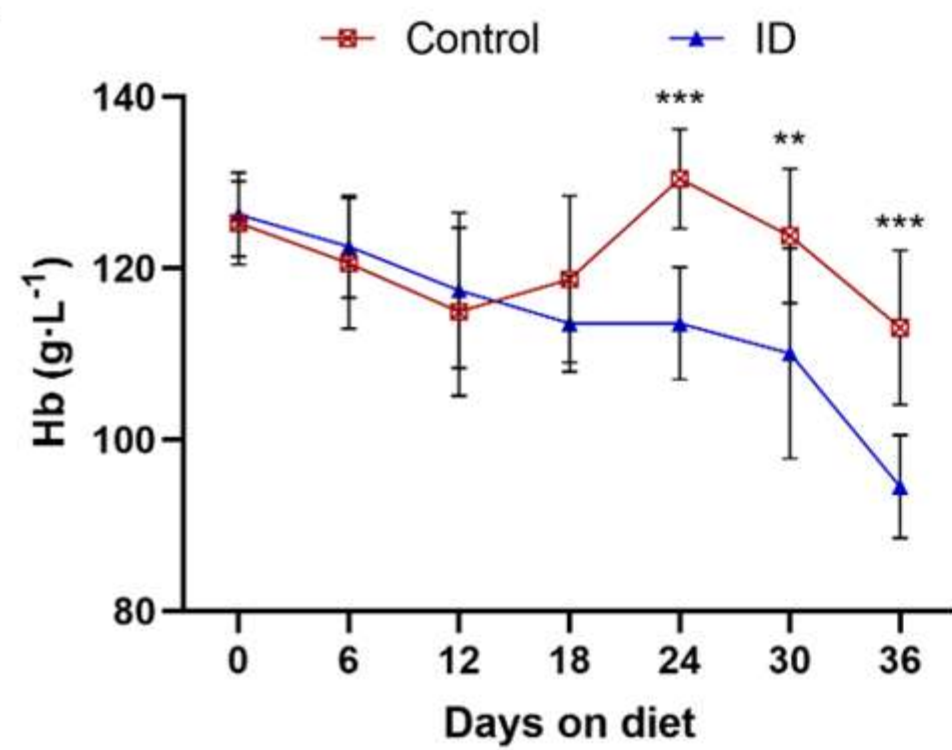**B**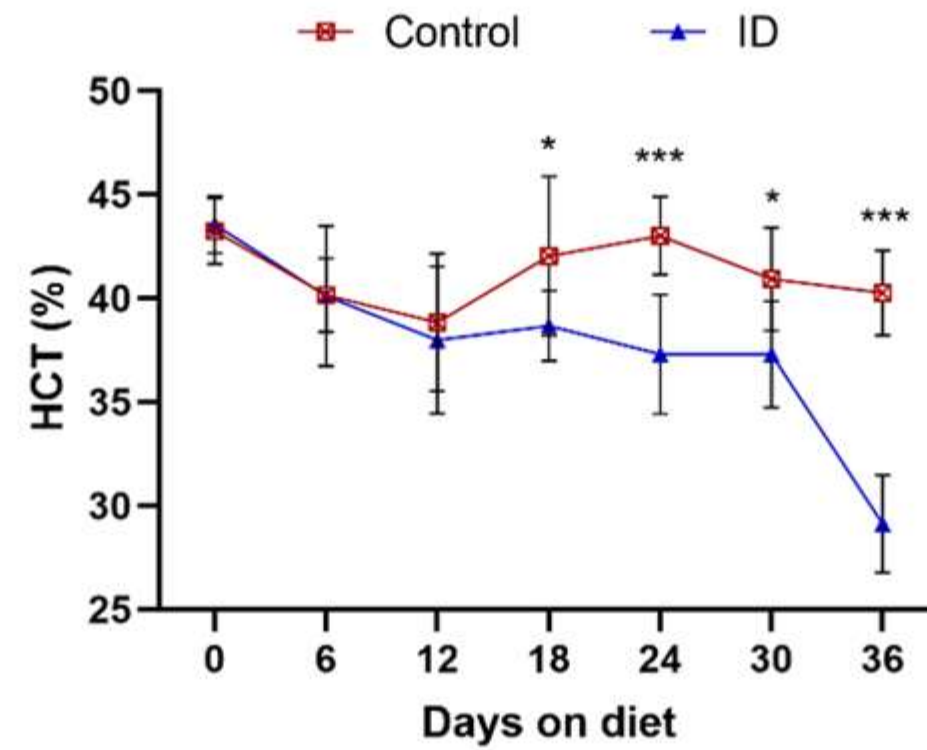**C**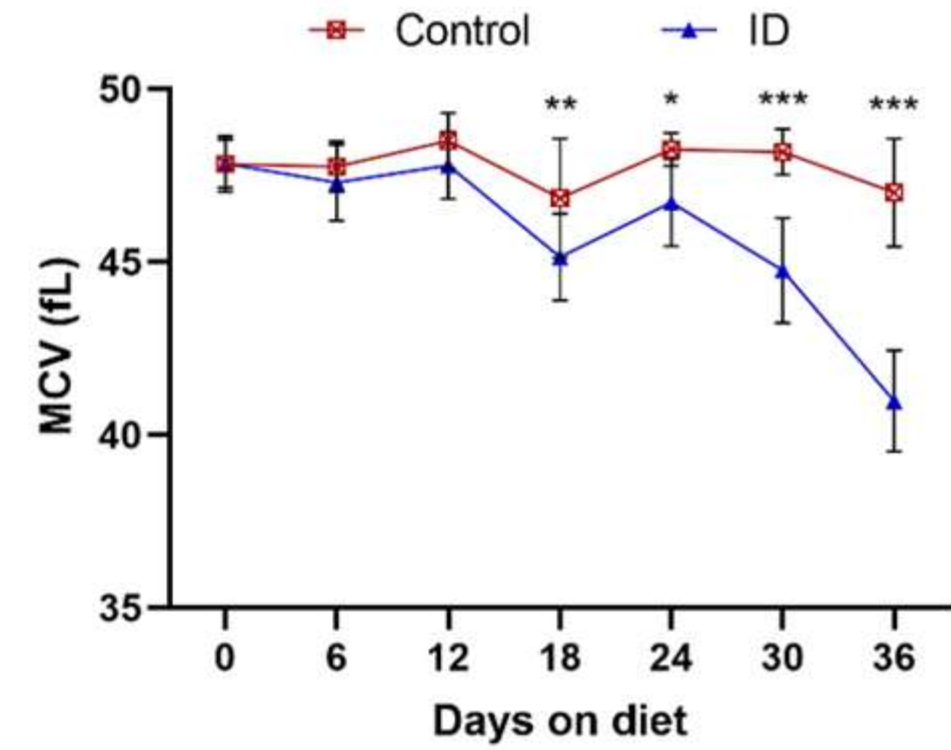**D**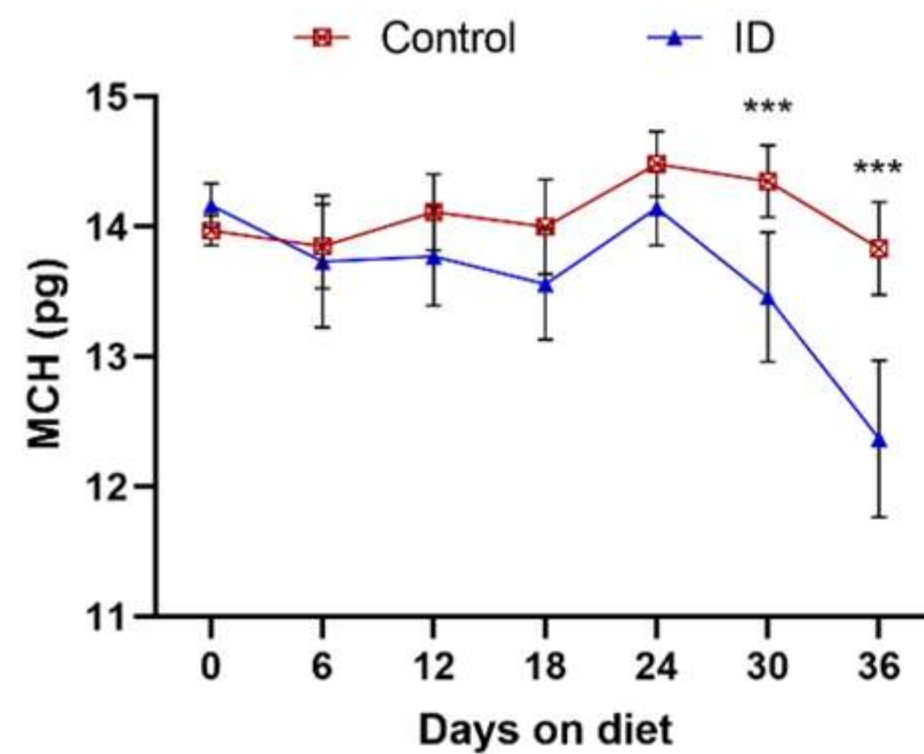**E**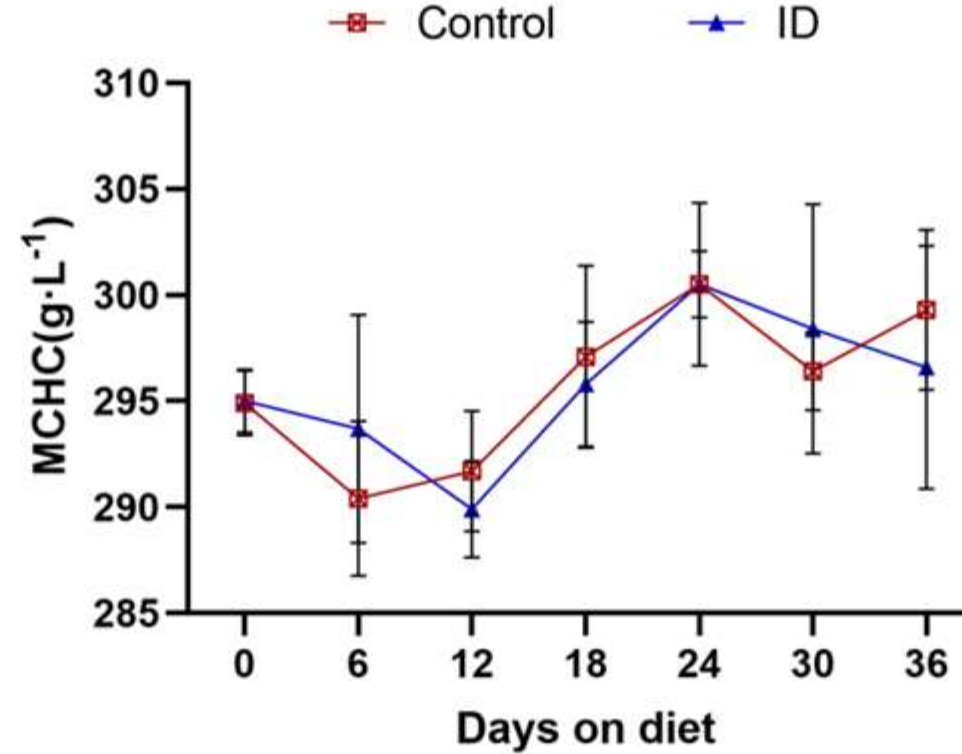**F**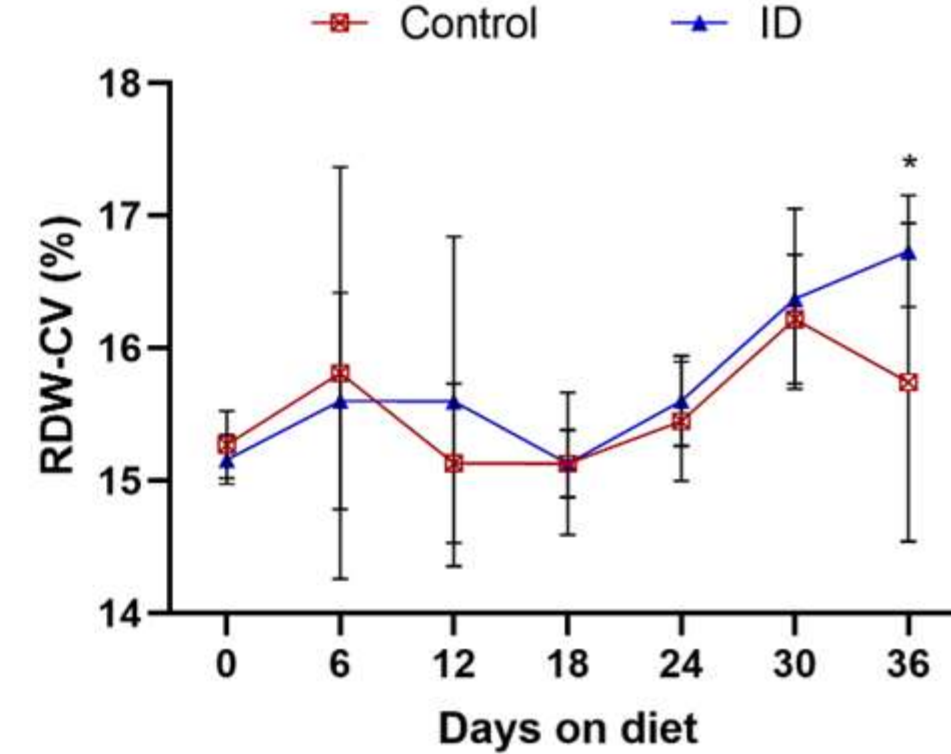**G**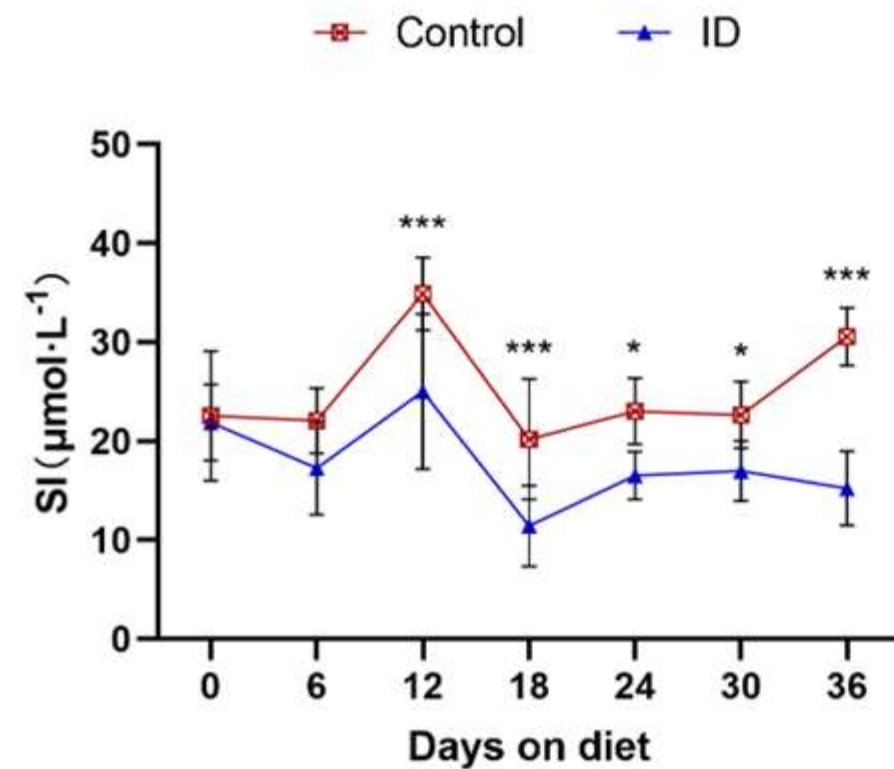**H**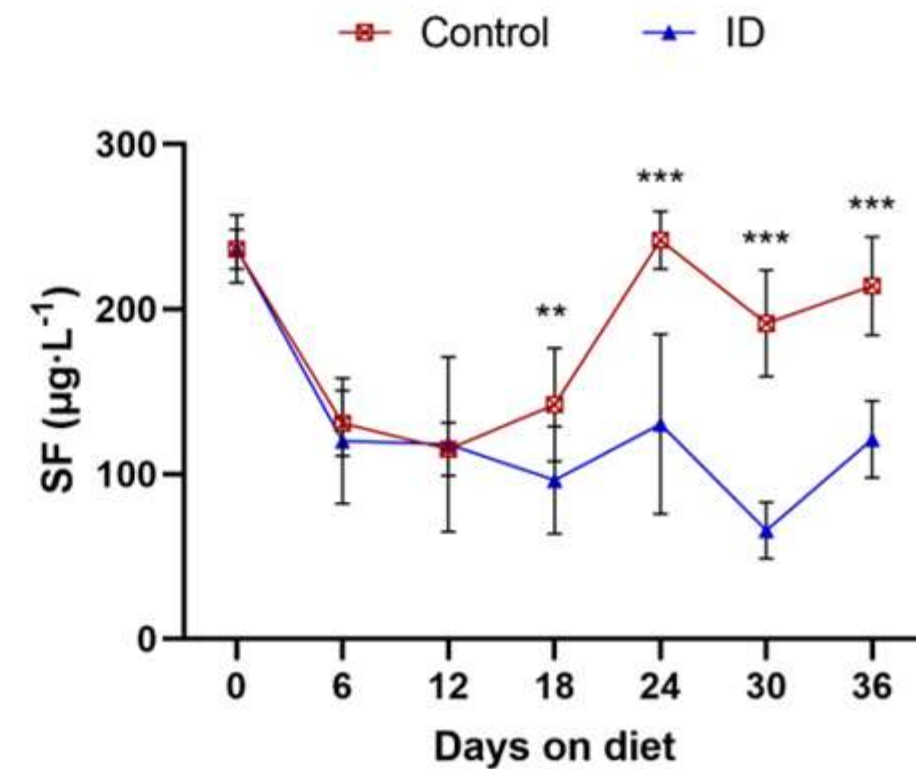

Supplement: Supplementary file 1 [file Data_Sheet_1.PDF]

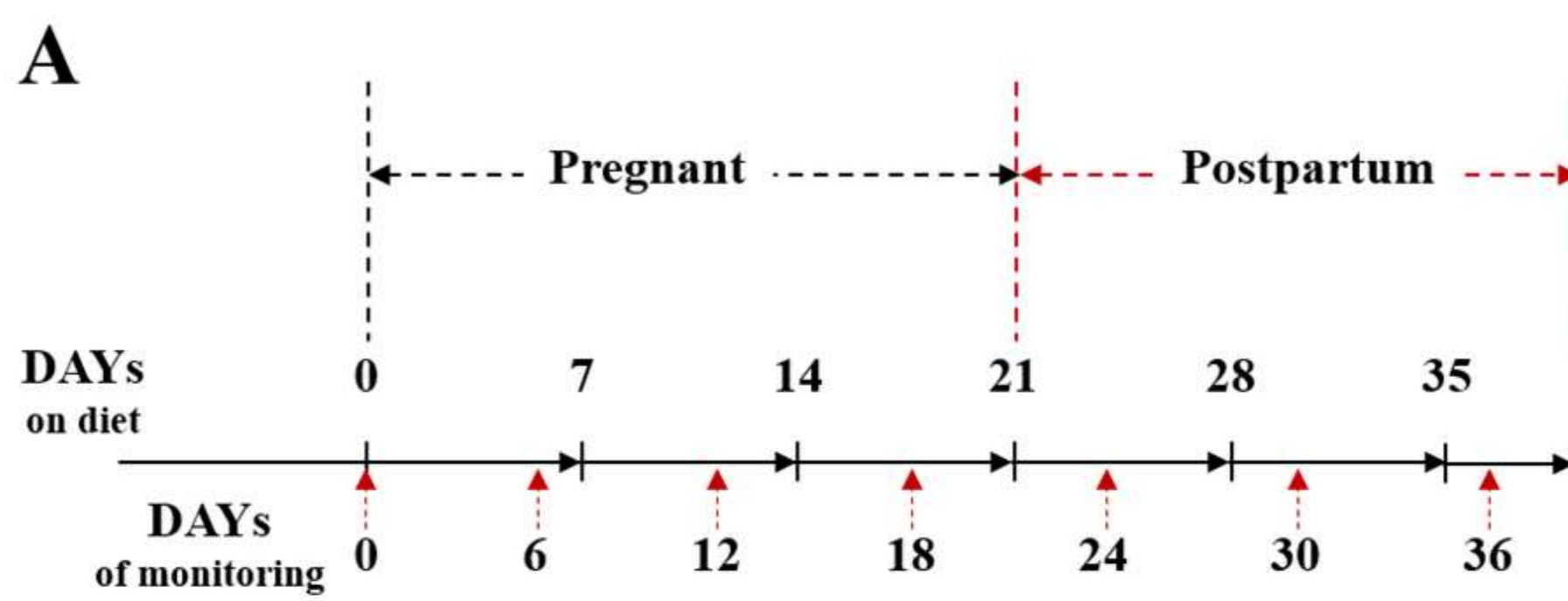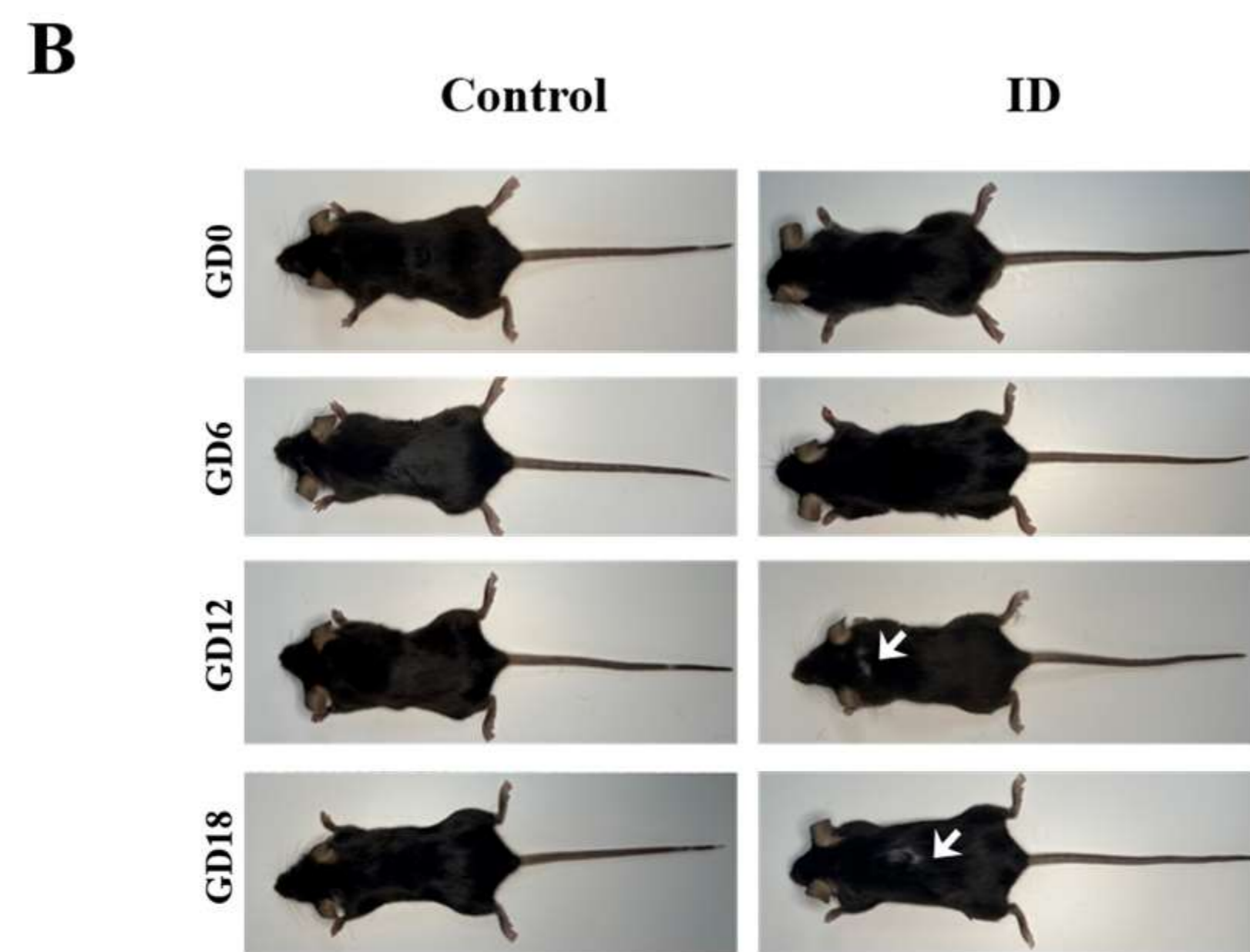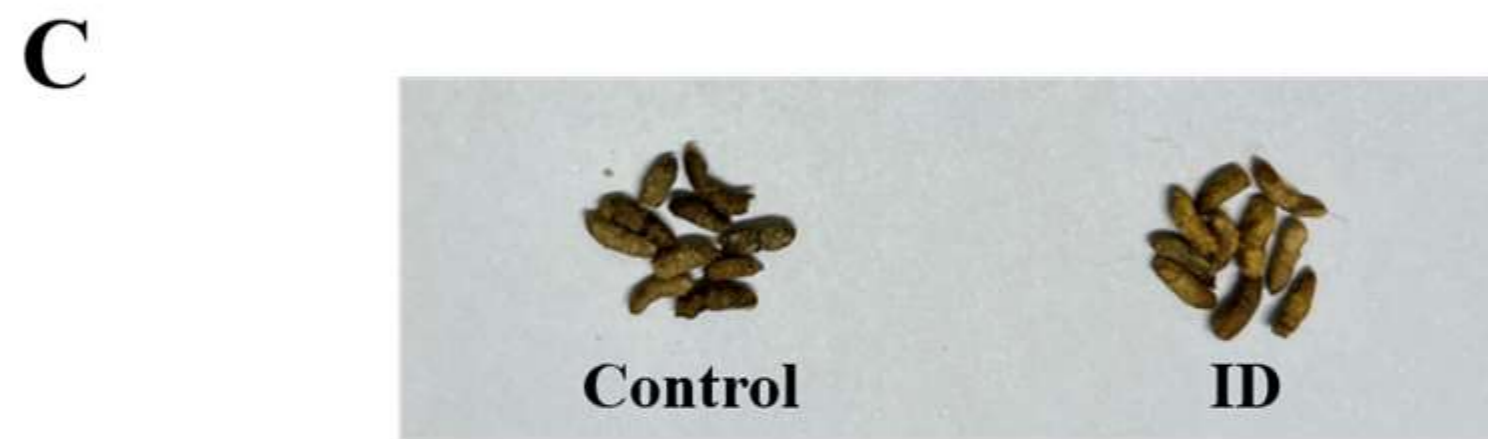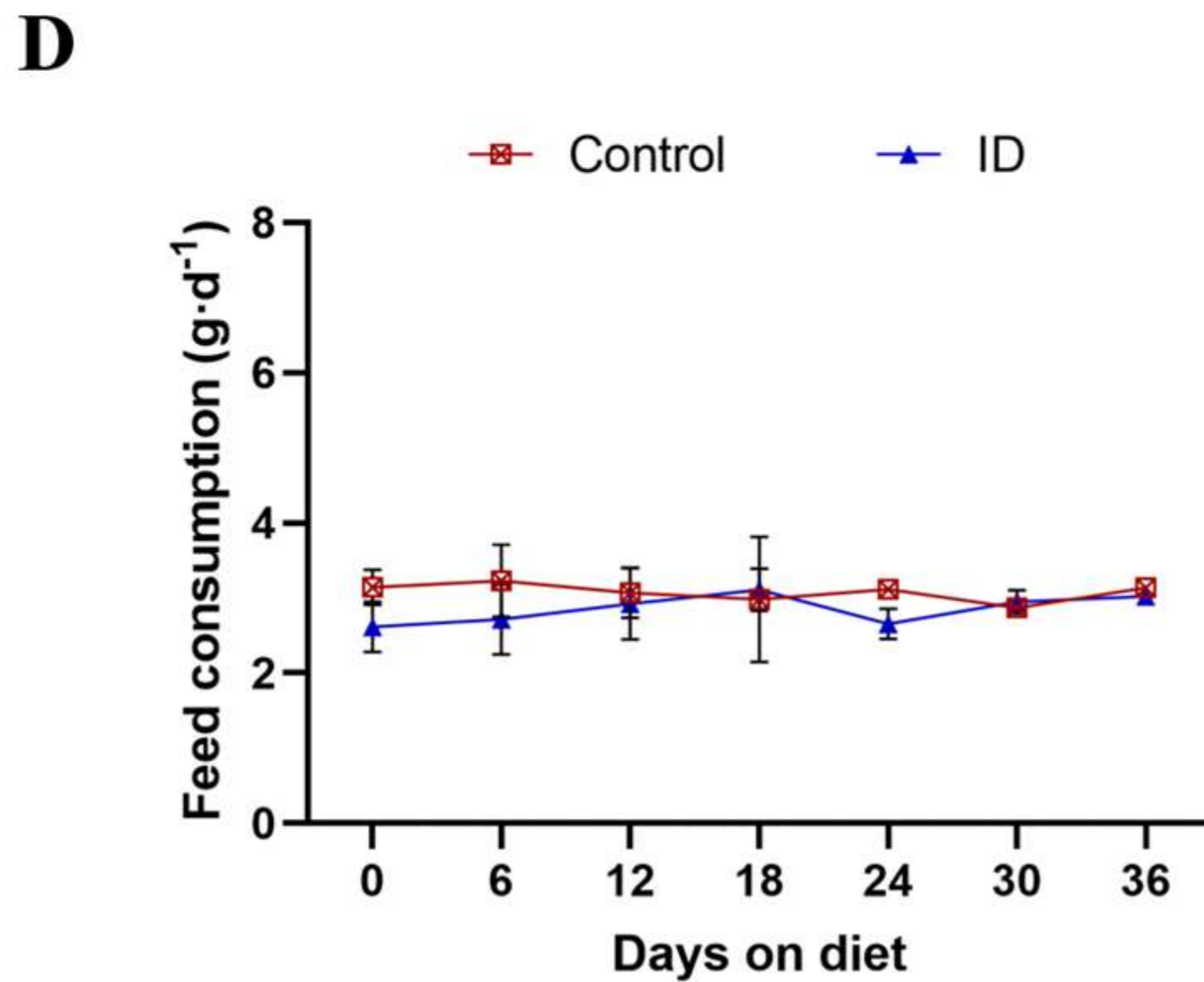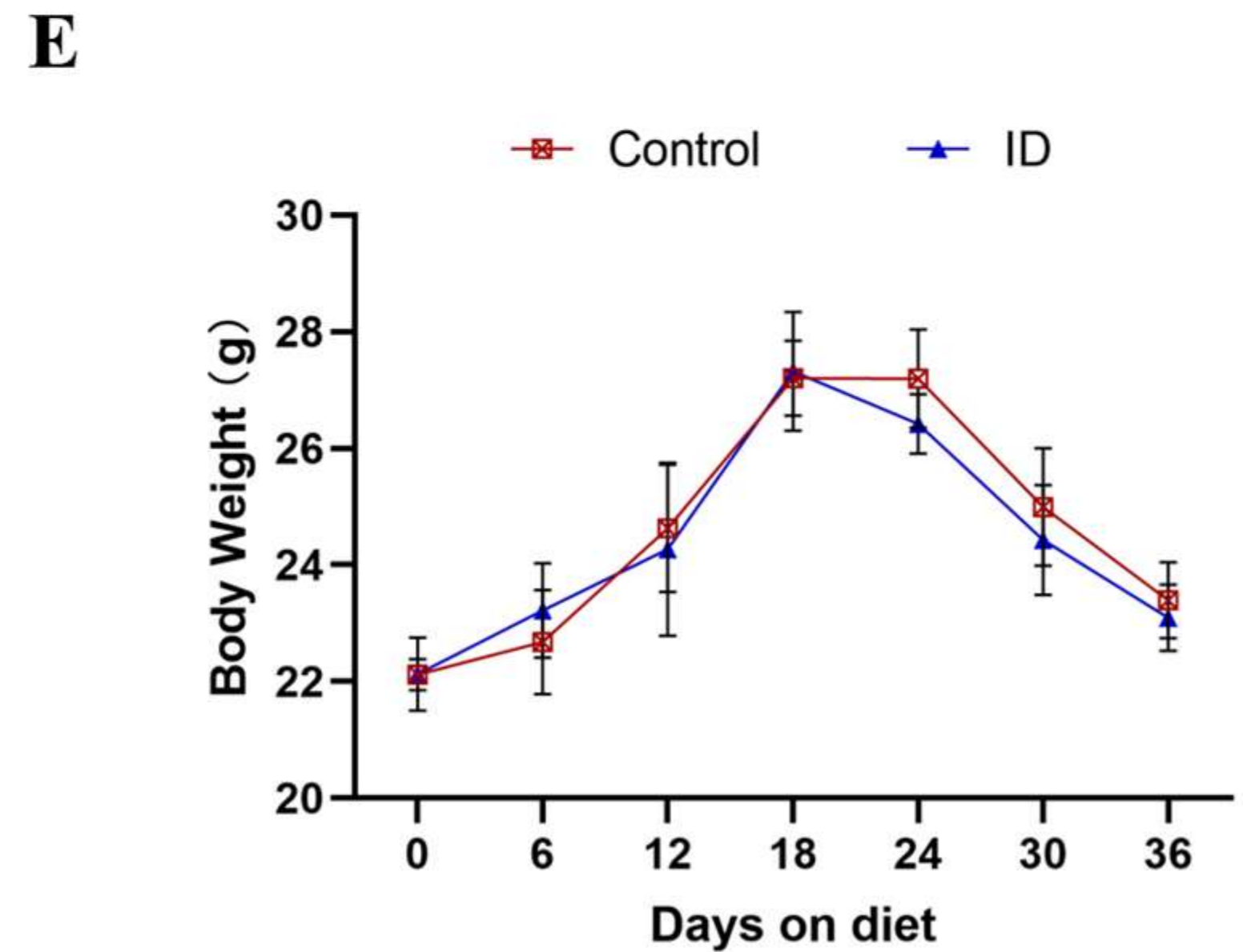

Supplement: Supplementary file 2 [file Data_Sheet_2.PDF]

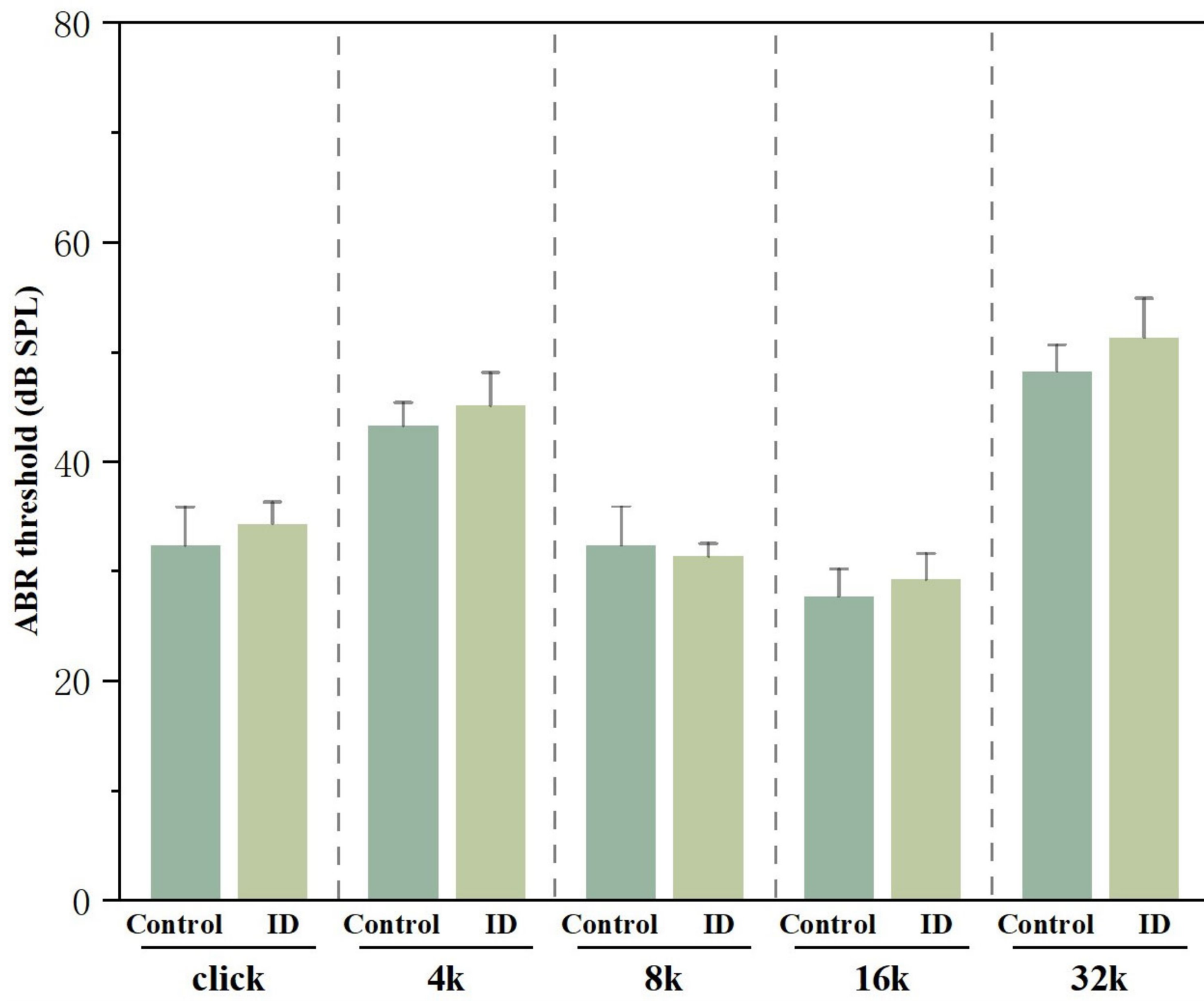

Supplement: Supplementary file 3 [file Data_Sheet_3.PDF]

**A**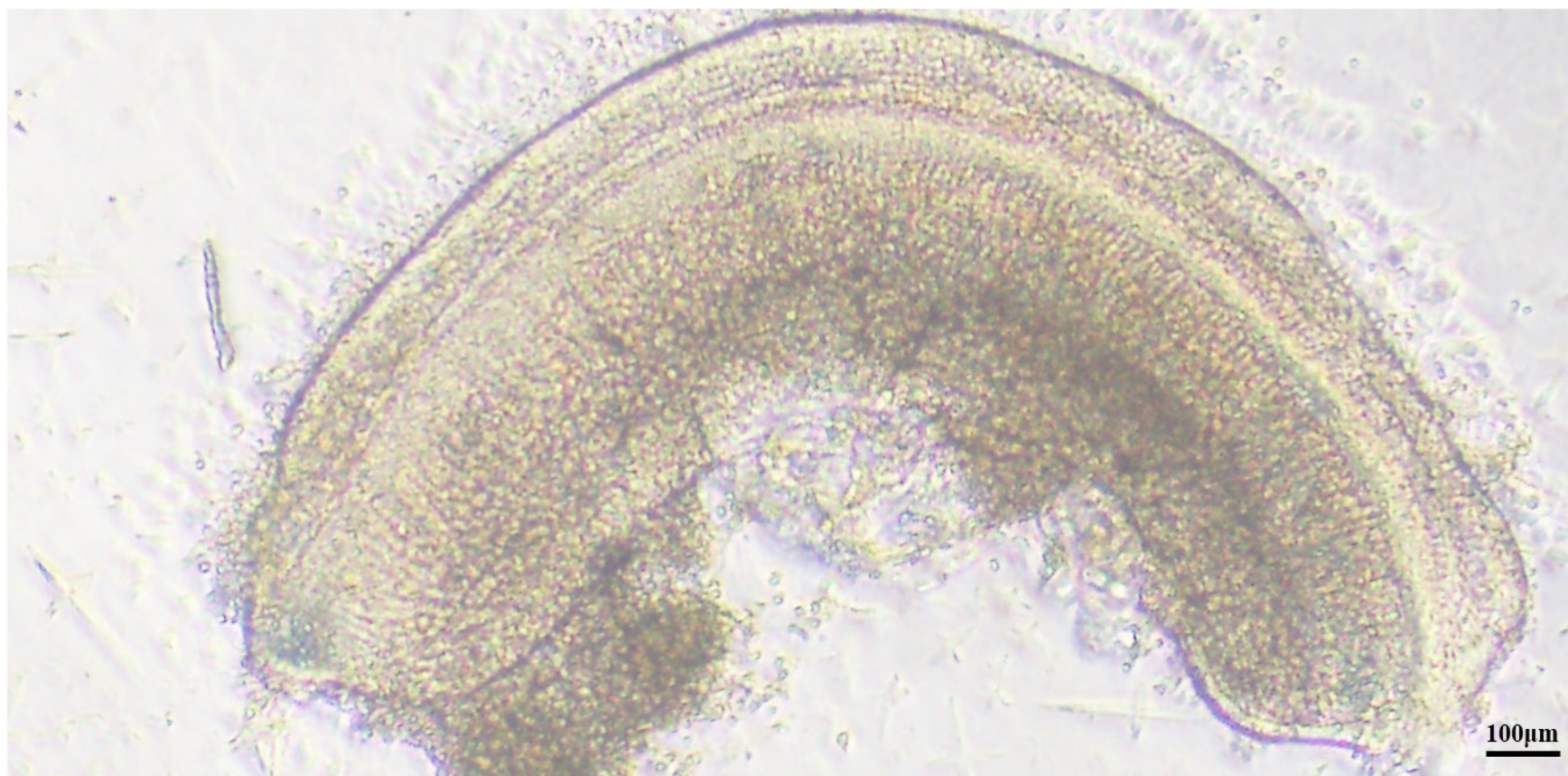**B**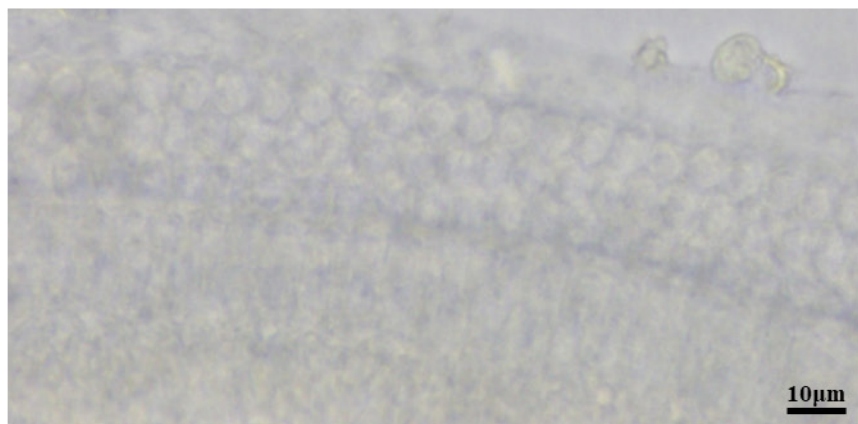**C**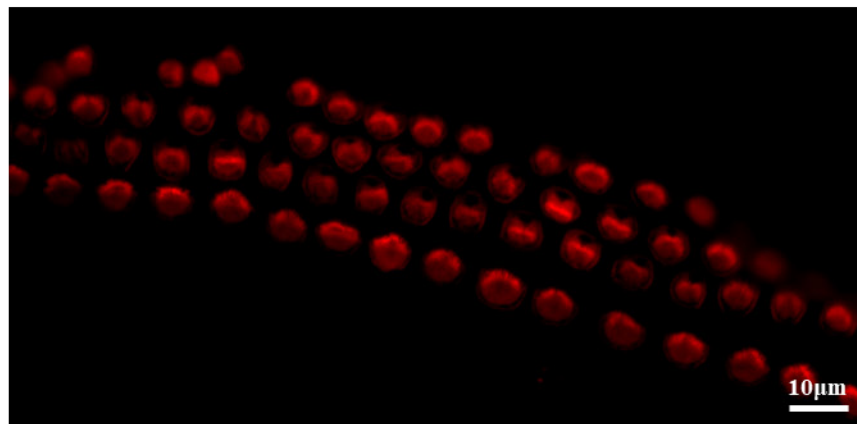**D**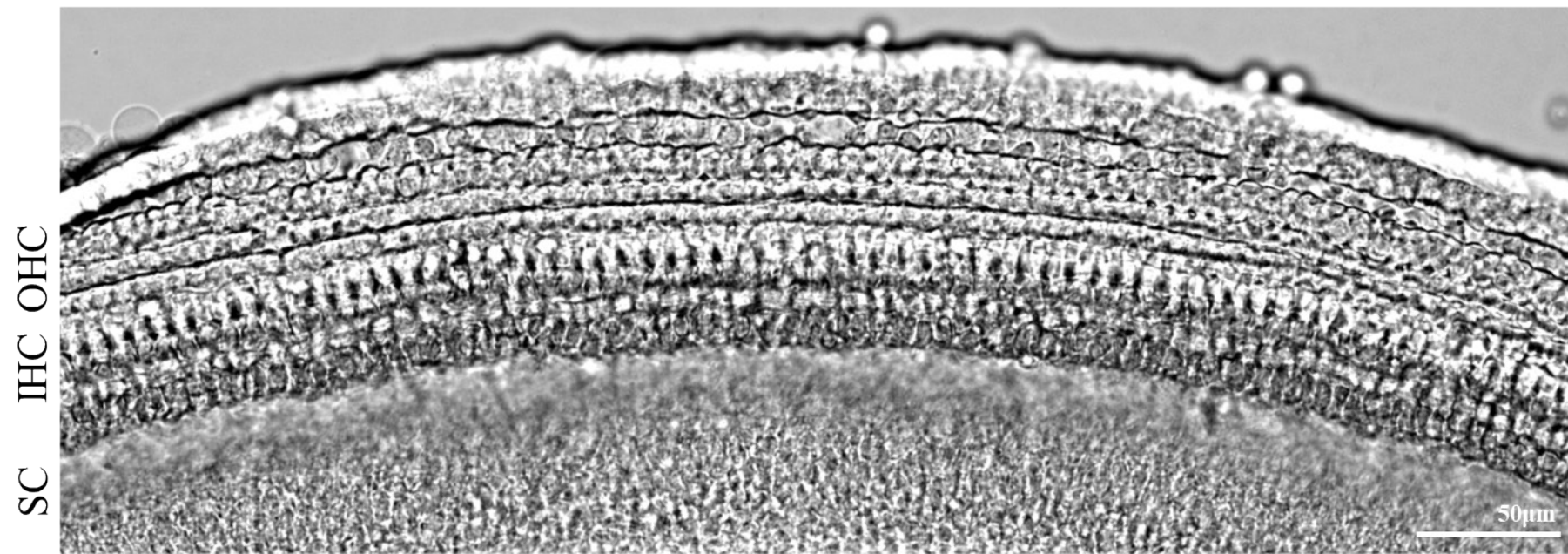

Supplement: Supplementary file 4 [file Data_Sheet_4.PDF]

**A**

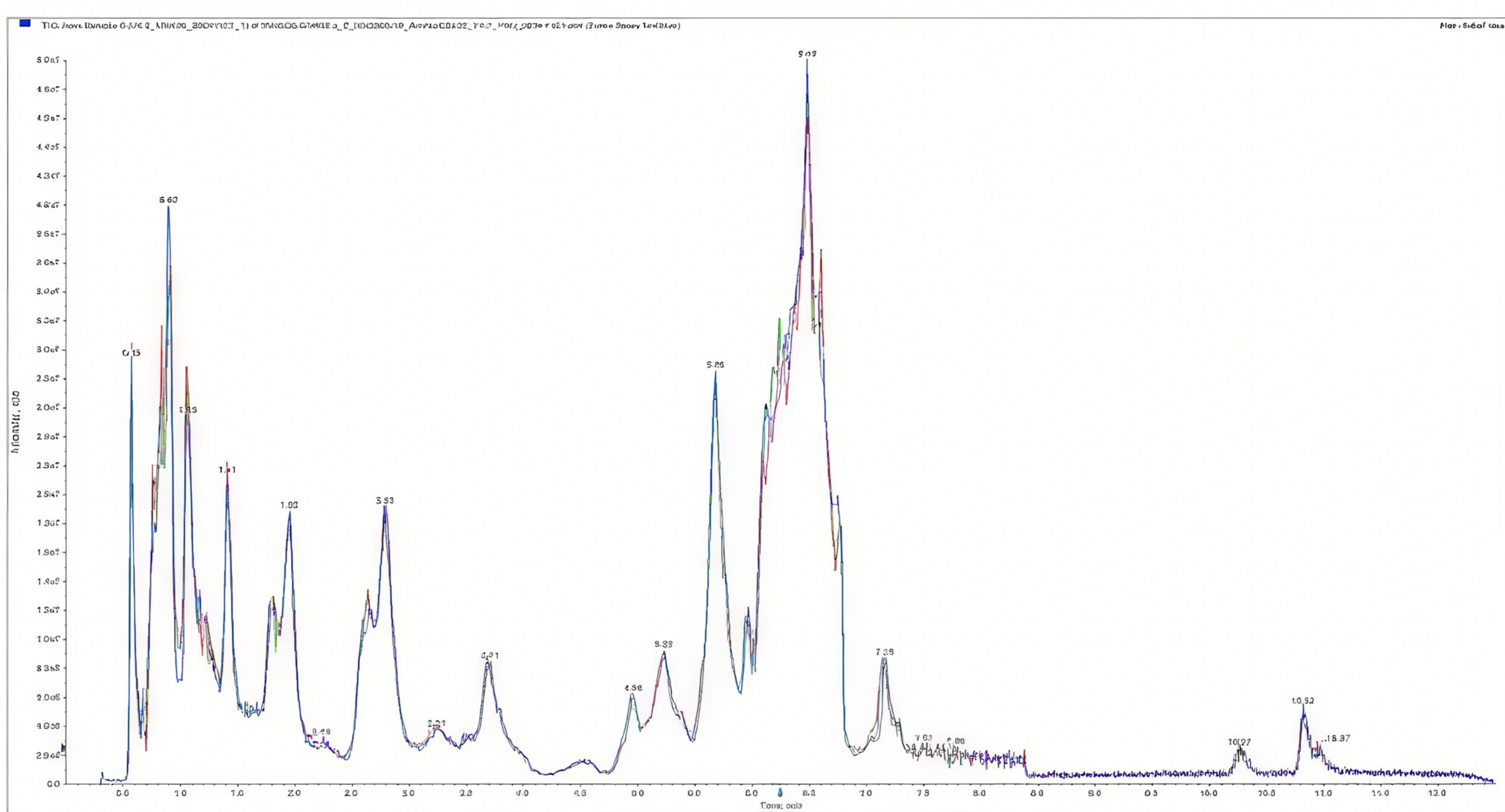

**B**

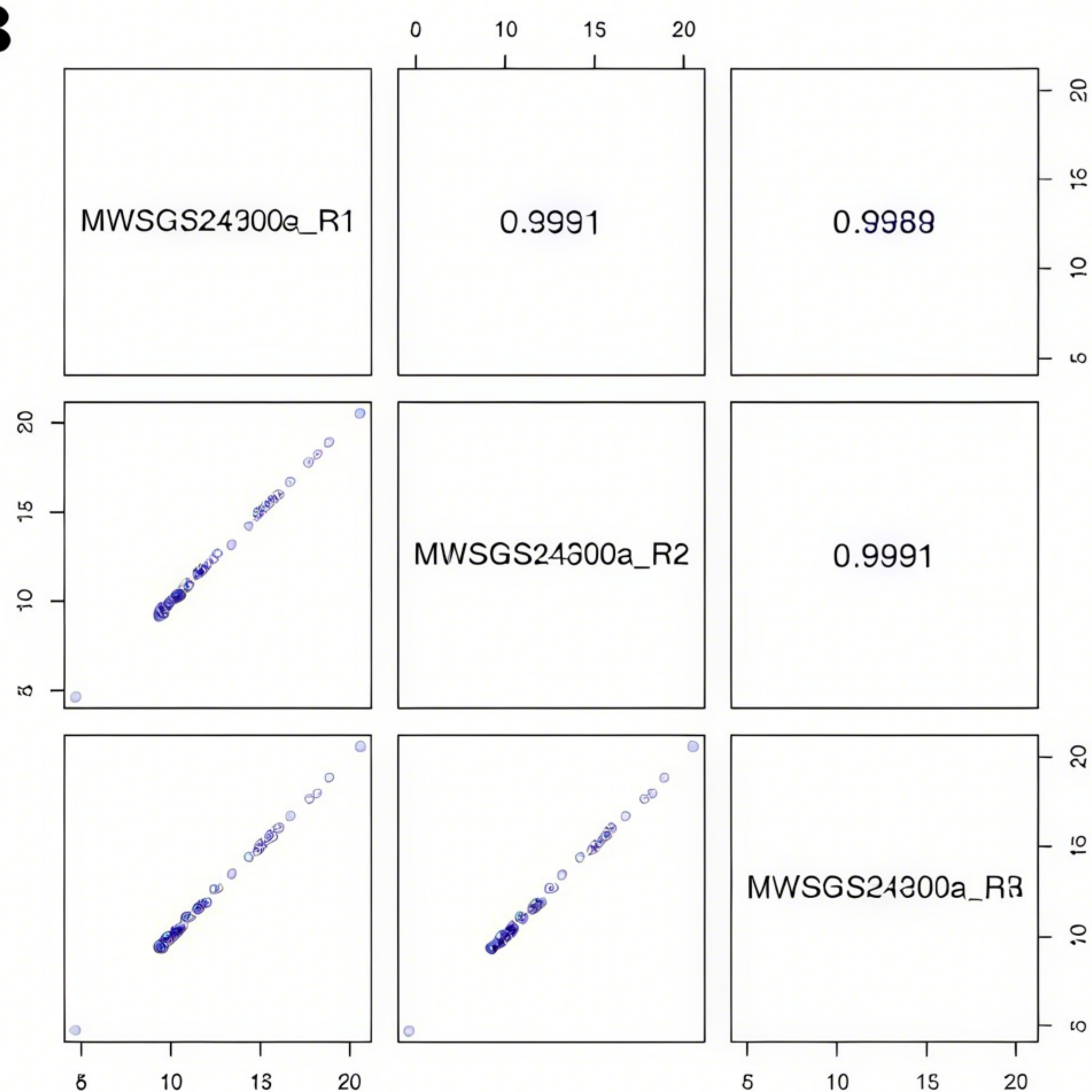

**C**

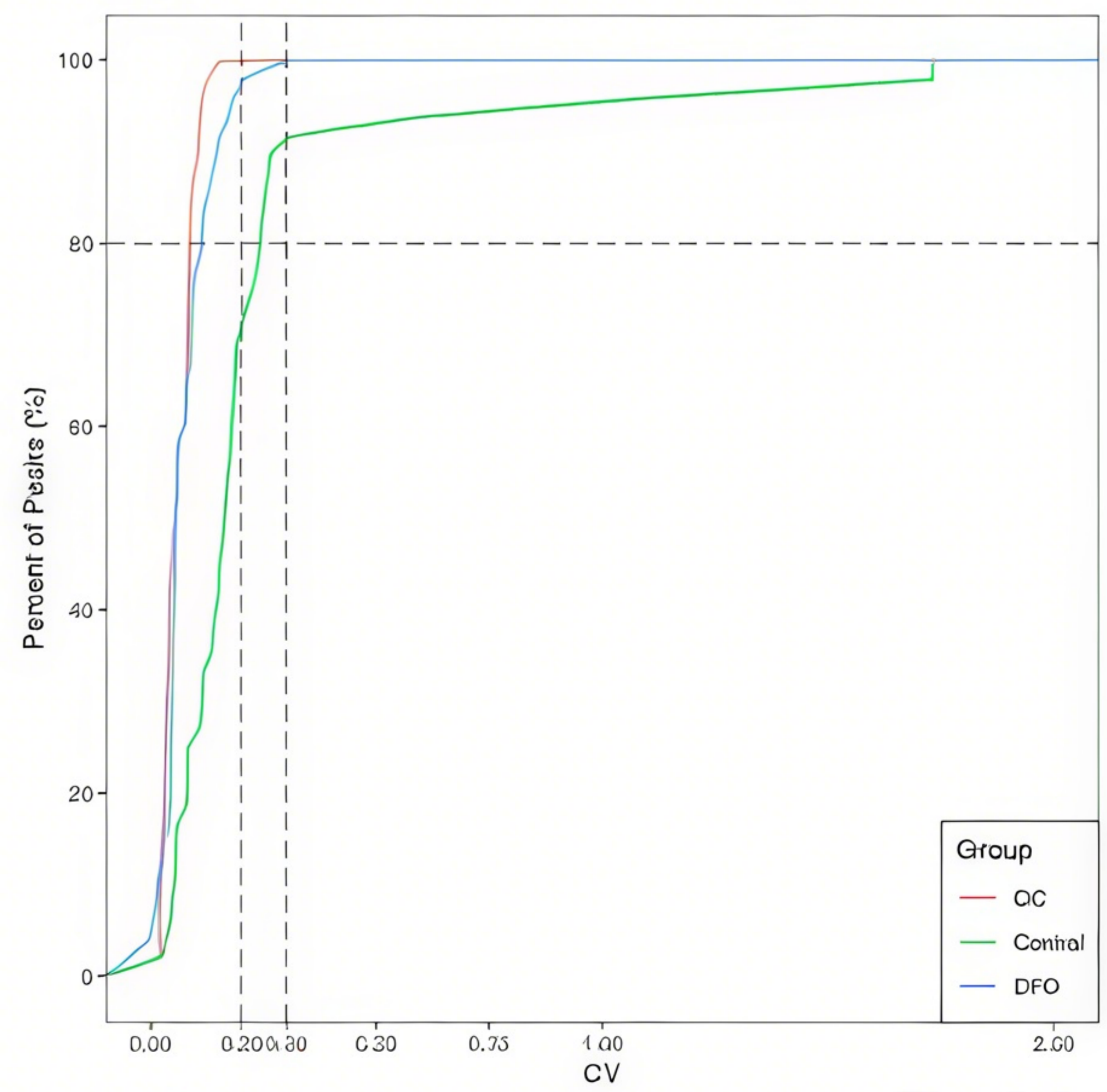

Supplement: Supplementary file 5 [file Data_Sheet_5.PDF]

**A**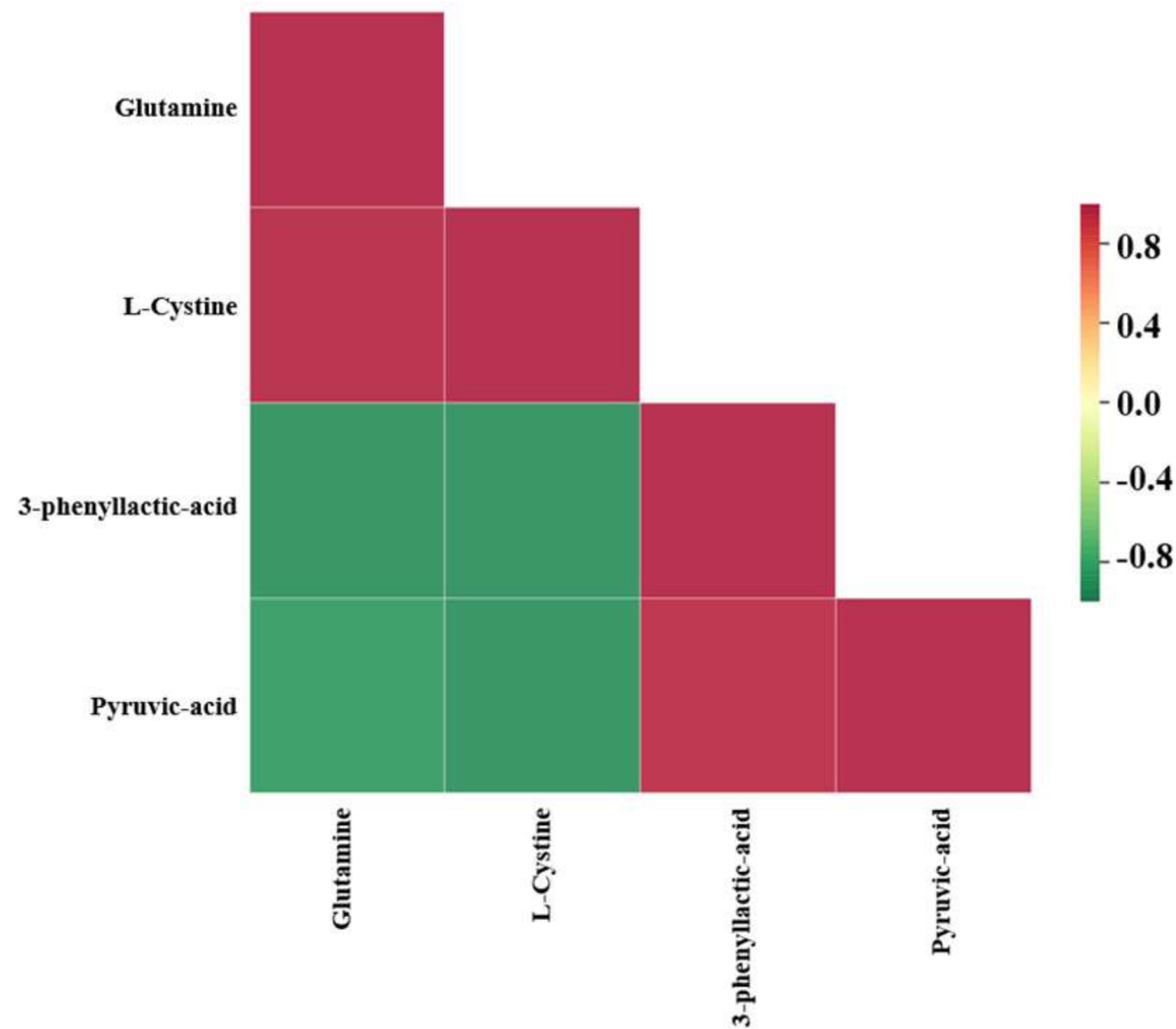**B**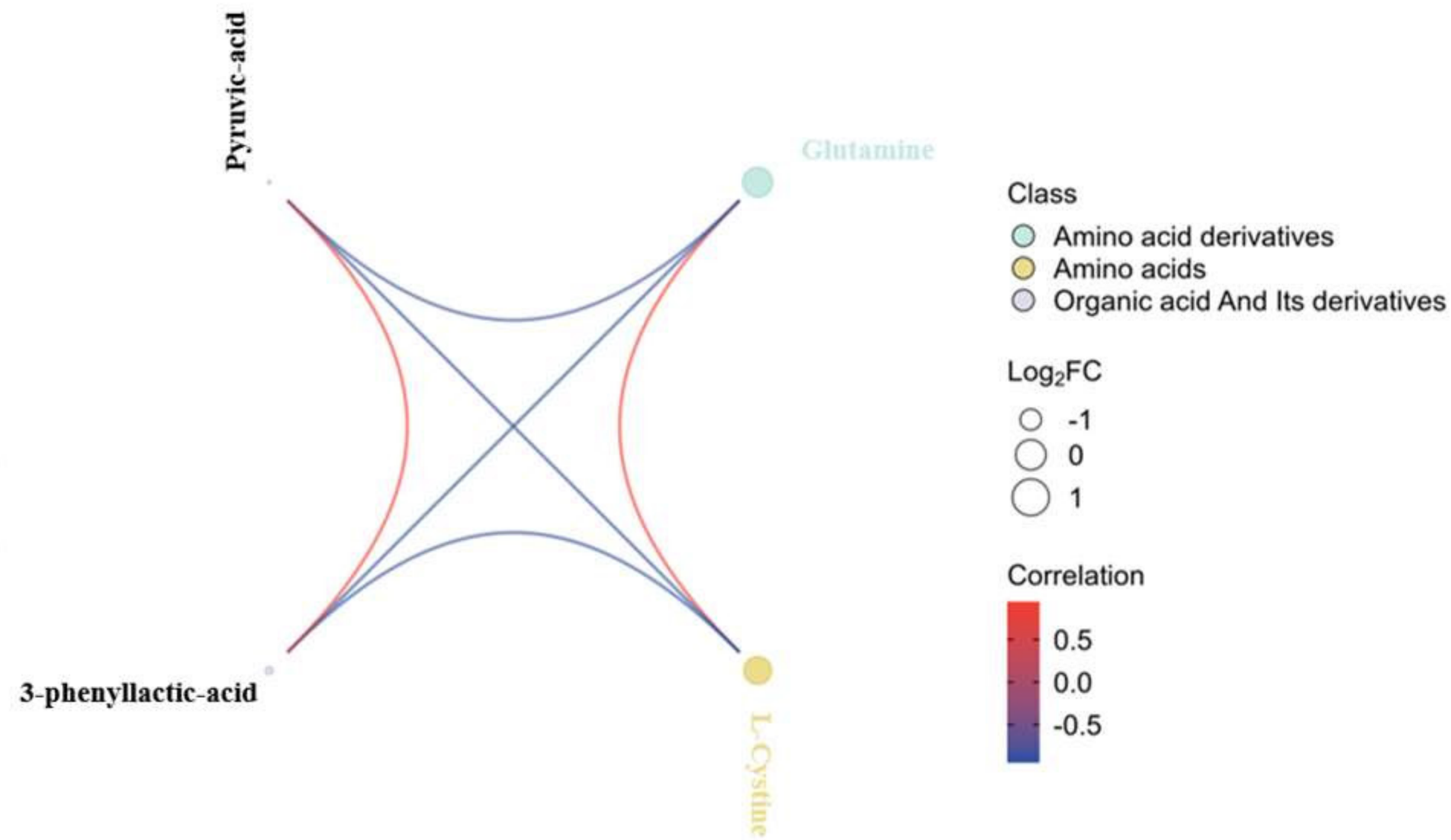

Supplement: Supplementary file 6 [file Data_Sheet_6.PDF]

# GLYCOLYSIS / GLUCONEOGENESIS

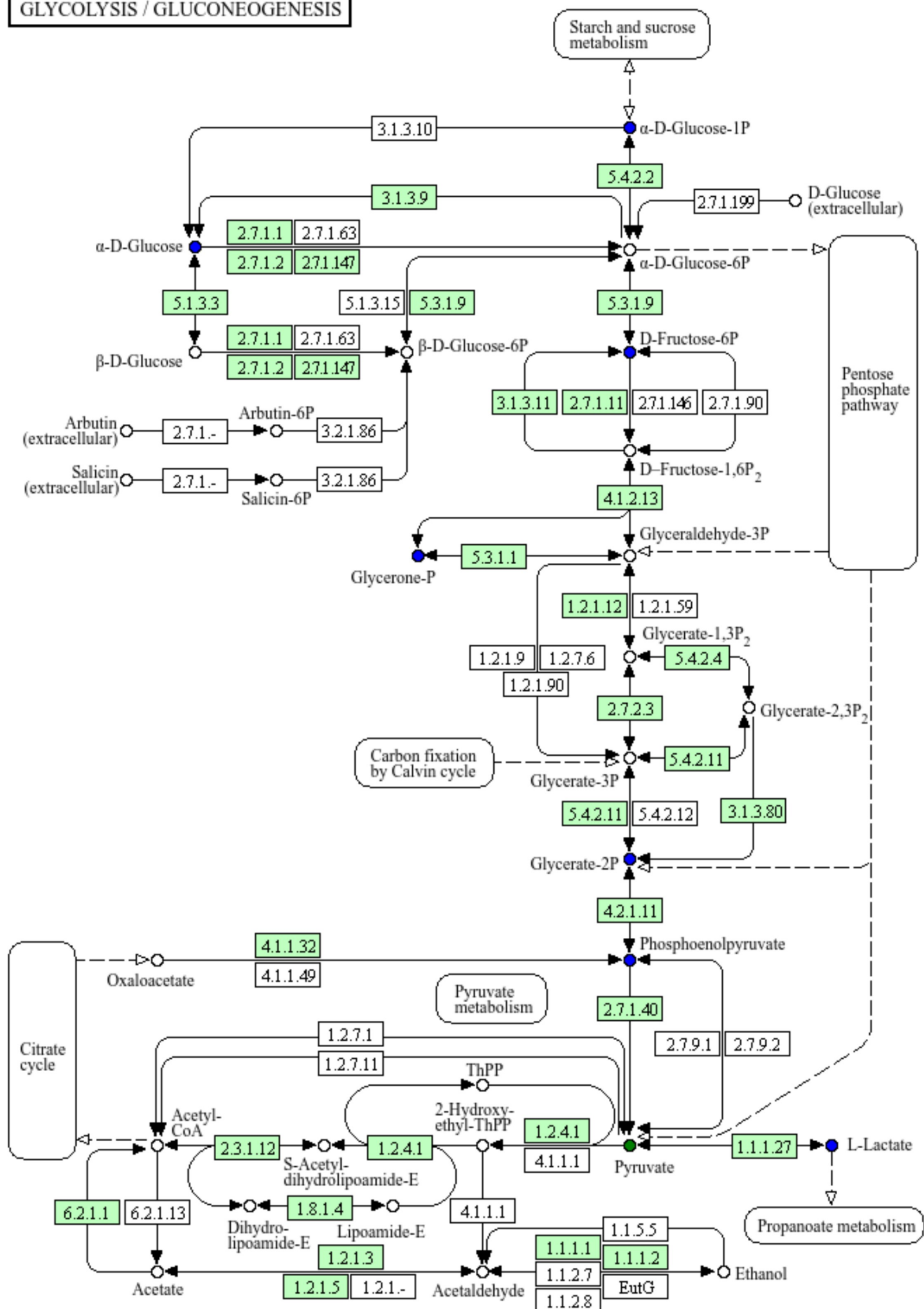

Supplement: Supplementary file 7 [file Data_Sheet_7.PDF]

# CITRATE CYCLE (TCA CYCLE)

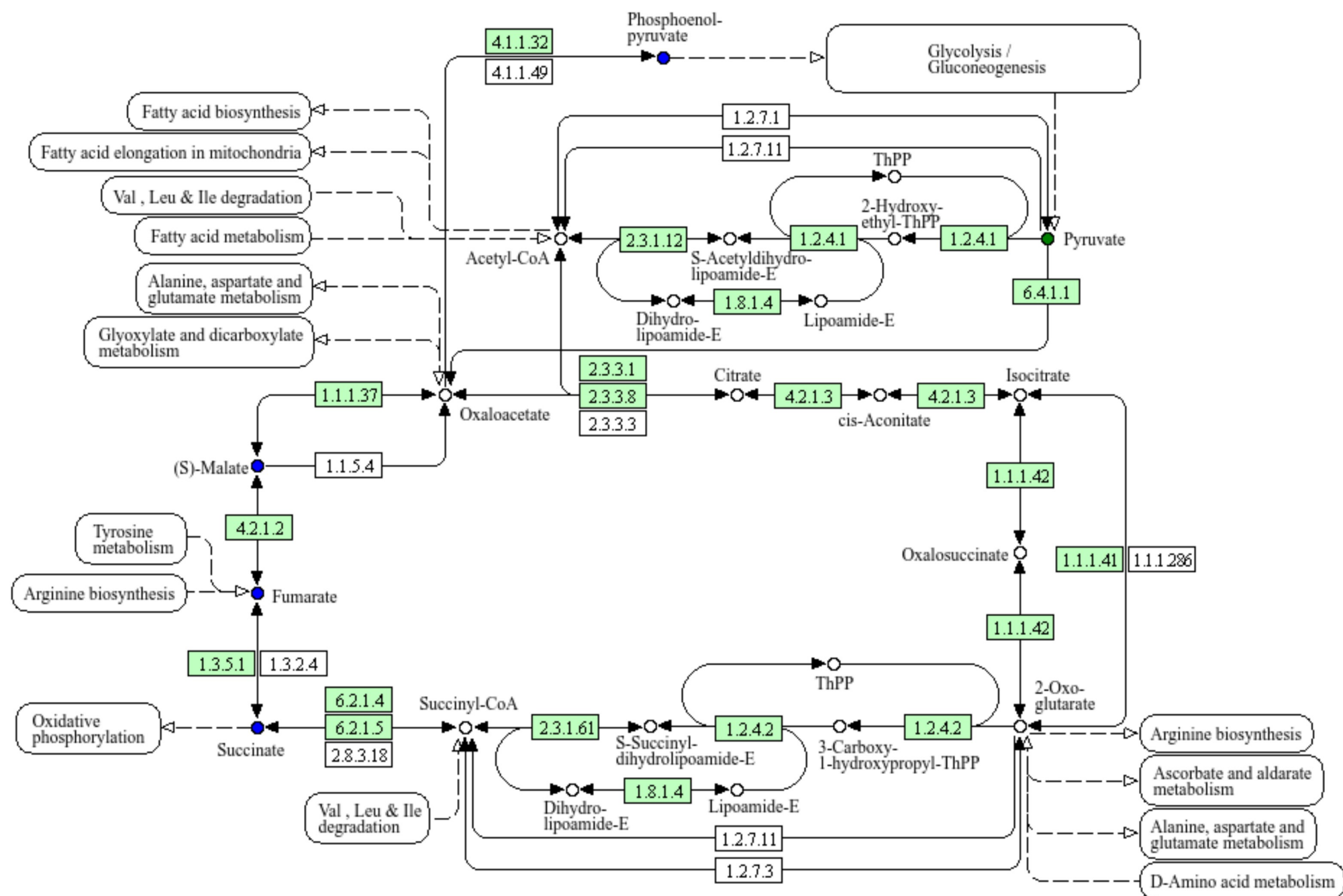

Supplement: Supplementary file 8 [file Data_Sheet_8.PDF]

**A**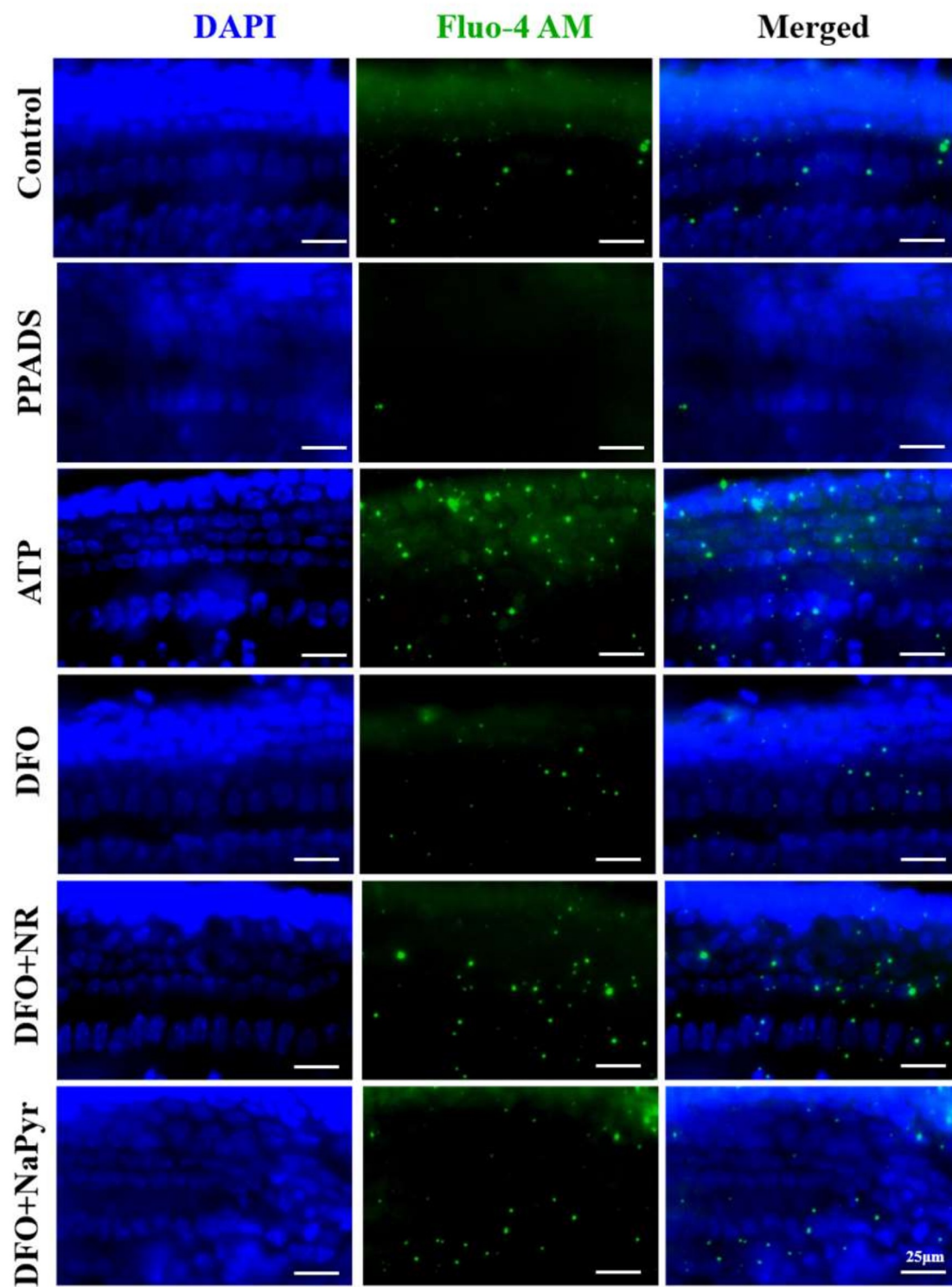**B**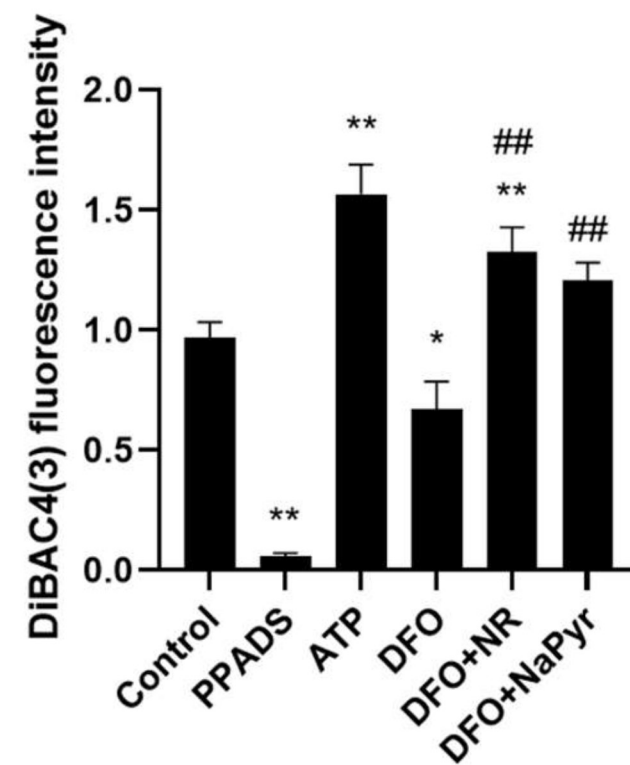

Supplement: Supplementary file 10 [file Data_Sheet_10.PDF]

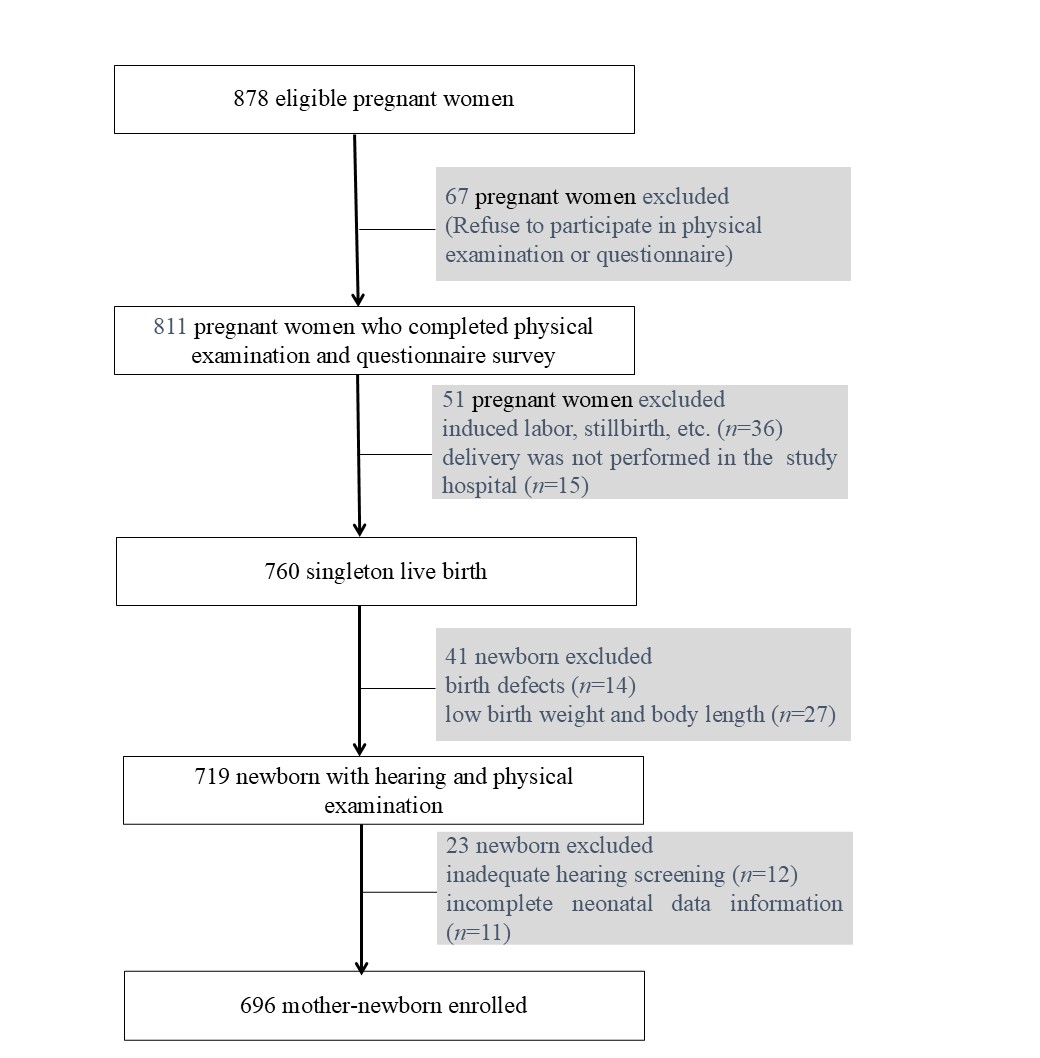

Supplement: Supplementary file 11 [file Image_1.JPEG]
