# Supplementary material for: Association between maternal iron deficiency and delayed neonatal auditory maturation and altered cochlear synaptic energy metabolism: analysis from a mother–infant observational study, mouse models, and cochlear explants
Source: Front Nutr. 2026 Jun 19;13:1842147. doi: 10.3389/fnut.2026.1842147 (PMC13328176; doi:10.3389/fnut.2026.1842147)
Supplement: Supplementary file 9 [file Data_Sheet_9.PDF]

OXIDATIVE PHOSPHORYLATION

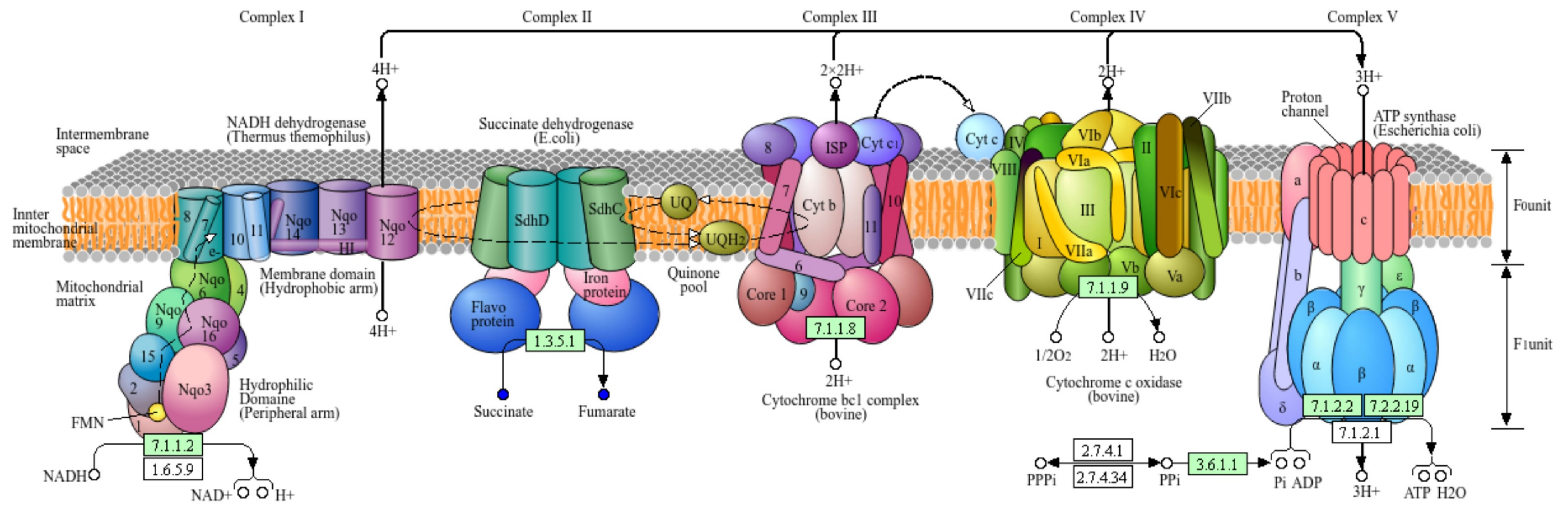

NADH dehydrogenase

E	ND1	ND2	ND3	ND4	ND4L	ND5	ND6											
E	Ndufs1	Ndufs2	Ndufs3	Ndufs4	Ndufs5	Ndufs6	Ndufs7	Ndufs8	Ndufv1	Ndufv2	Ndufv3							
B/A	NuoA	NuoB	NuoC	NuoD	NuoE	NuoF	NuoG	NuoH	NuoI	NuoJ	NuoK	NuoL	NuoM	NuoN				
E/B	NdhC	NdhK	NdhJ	NdhH	NdhA	NdhI	NdhG	NdhE	NdhF	NdhD	NdhB	NdhL	NdhM	NdhN	HoxE	HoxF	HoxU	
E	Ndufa1	Ndufa2	Ndufa3	Ndufa4	Ndufa5	Ndufa6	Ndufa7	Ndufa8	Ndufa9	Ndufa10	Ndufab1	Ndufa11	Ndufa12	Ndufa13				
E	Ndufb1	Ndufb2	Ndufb3	Ndufb4	Ndufb5	Ndufb6	Ndufb7	Ndufb8	Ndufb9	Ndufb10	Ndufb11	Ndufc1	Ndufc2					

Succinate dehydrogenase / Fumarate reductase

E	SDHC	SDHD	SDHA	SDHB
B/A	SdhC	SdhD	SdhA	SdhB
		FrdA	FrdB	FrdC	FrdD

Cytochrome c reductase

E/B/A	ISP	Cyt b	Cyt 1						
E				COR1	QCR2	QCR6	QCR7	QCR8	QCR9	QCR10

Cytochrome c oxidase

E	COX10	COX3	COX1	COX2	COX4	COX5A	COX5B	COX6A	COX6B	COX6C	COX7A	COX7B	COX7C	COX8	COX11 (E/B/A)	COX15 (E/B/A)	COX17
B/A	CyoE	CyoD	CyoC	CyoB	CyoA												
		CoxD	CoxC	CoxA	CoxB												
		QoxD	QoxC	QoxB	QoxA												
		SoxD	SoxC	SoxB	SoxA												

Cytochrome c oxidase , cbb3-type

B	I	II	IV	III

Cytochrome bd complex

B/A	CydA	CydB	CydX

Cytochrome c

CYC

F-type ATPase (Bacteria)

alpha	beta	gamma	delta	epsilon
a	b	c		

F-type ATPase (Eukaryotes)

alpha	beta	gamma	delta	epsilon	
OSCP	a	b	c	d	e
f	g	f6/h	j	k	8

V/A-type ATPase (Bacteria, Archaea)

A	B	C	D	E	F	G/H
I	K					

V-type ATPase (Eukaryotes)

A	B	C	D	E	F	G	H
a	c	d	e	S1			

00190 12/15/23

(c) Kanehisa Laboratories
