# Supplementary material for: Association between maternal iron deficiency and delayed neonatal auditory maturation and altered cochlear synaptic energy metabolism: analysis from a mother–infant observational study, mouse models, and cochlear explants
Source: Front Nutr. 2026 Jun 19;13:1842147. doi: 10.3389/fnut.2026.1842147 (PMC13328176; doi:10.3389/fnut.2026.1842147)
Supplement: Supplementary file 12 [file Supplementary_file_1.docx]

**Supplemental Table 1** Composition of control and iron deﬁcient diets

| Ingredients (g/kg Diet) | Control | Iron deﬁcient |
| --- | --- | --- |
| Cornstarch | 529.5 | 529.92 |
| Casein (>85% protein) | 200.0 | 200.0 |
| Sucrose | 100.0 | 100.0 |
| Soybean oil | 70.0 | 70.0 |
| Fiber | 50.0 | 50.0 |
| Mineral mix (Ferric citrate) | 35.0 (0.735) | 34.58 (0.315) |
| Vitamin mix | 10.0 | 10.0 |
| L-Cysteine | 3.0 | 3.0 |
| Choline bitartrate | 2.5 | 2.5 |

**Supplemental Table 2** Growth and iron outcomes of pups

| Indicators | Control | Iron deﬁcient | |
| --- | --- | --- | --- |
| Number of successful delivery | 10 | 10 | |
| PND 4 sex ratio (male:female) | 52:47 | 56:44 | |
| PND 7 body weights (g)^a^ | 4.60 ± 0.21 | | 4.63 ± 0.23 |
| PND 14 body weights (g)^a^ | 9.24 ± 0.18 | | 9.15 ± 0.14 |
| PND 21 Dams |  | |  |
| Hb (g/L) | 120.60 ± 23.25 | | 116.09 ± 25.07 |
| SI (μmol/L) | 41.38 ± 12.05 | | 39.60 ± 8.15 |
| SF (ng/mL) ^a^ | 6.12 ± 2.35 | | 4.05 ± 1.66 ^*^ |
| HCT (%) | 42.20 ± 15.02 | | 37.50 ± 14.46 |
| RDW (%) | 18.10± 2.35 | | 24.70 ± 1.95^*^ |
| PND 21 Pups |  | |  |
| Hb (g/L)^a^ | 116.17 ±24.07 | | 115.29 ± 25.53 |
| SF (ng/mL) ^a^ | 5.27 ± 1.26 | | 3.20 ± 1.03^*^ |
| Amplitudes (μV) of wave I in 90 dB SPL^a^ | 6.63 ± 1.59 | | 4.21 ± 1.08^*^ |
| Wave I latency (ms) | 1.34 ± 0.19 | 1.38 ± 0.22 | |

Hb, hemoglobin; Hct, hematocrit; PND, postnatal day; RDW, red cell distribution width; SF, serum ferritin; SI, serum level of iron; SPL, sound pressure level SPL. a Mean ± SD;

* *p* < 0.05, compared to control.

**Supplemental Table 3** Differential metabolite screening

| Index | Compounds | *P* | Type |
| --- | --- | --- | --- |
| Pyruvic-acid | Pyruvic acid | 0.045 | down |
| 3-phenyllactic-acid | 3-phenyllactic acid | 0.020 | down |
| L-Cystine | L-Cystine | 0.009 | up |
| Glutamine | Glutamine | <0.001 | up |
